# Multiple Abiotic Stresses Applied Simultaneously Elicit Distinct Responses in Two Contrasting Rice Cultivars

**DOI:** 10.3390/ijms23031739

**Published:** 2022-02-03

**Authors:** Fatemeh Habibpourmehraban, Yunqi Wu, Jemma X. Wu, Sara Hamzelou, Farhad Masoomi-Aladizgeh, Karthik Shantharam Kamath, Ardeshir Amirkhani, Brian J. Atwell, Paul A. Haynes

**Affiliations:** 1 Department of Molecular Sciences, Faculty of Science and Engineering, Macquarie University, North Ryde, NSW 2109, Australia; Fatemeh.habibpour-mehraban@hdr.mq.edu.au (F.H.); Yunqi.wu@mq.edu.au (Y.W.); Jemma.wu@mq.edu.au (J.X.W.); Sara.hamzelou@mq.edu.au (S.H.); Karthik.kamath@hdr.mq.edu.au (K.S.K.); Ardeshir.amirkhani@mq.edu.au (A.A.); 2 Biomolecular Discovery Research Centre, Macquarie University, North Ryde, NSW 2109, Australia; 3 Australian Proteome Analysis Facility (APAF), Macquarie University, North Ryde, NSW 2109, Australia; 4 Department of Biological Sciences, Faculty of Science and Engineering, Macquarie University, North Ryde, NSW 2109, Australia; Farhad.masoomi-aladizgeh@hdr.mq.edu.au (F.M.-A.); Brian.atwell@mq.edu.au (B.J.A.)

**Keywords:** rice, multiple abiotic stress, proteomics, physiology, TMT labeling, heat shock proteins

## Abstract

Rice crops are often subject to multiple abiotic stresses simultaneously in both natural and cultivated environments, resulting in yield reductions beyond those expected from single stress. We report physiological changes after a 4 day exposure to combined drought, salt and extreme temperature treatments, following a 2 day salinity pre-treatment in two rice genotypes—Nipponbare (a paddy rice) and IAC1131 (an upland landrace). Stomata closed after two days of combined stresses, causing intercellular CO2 concentrations and assimilation rates to diminish rapidly. Abscisic acid (ABA) levels increased at least five-fold but did not differ significantly between the genotypes. Tandem Mass Tag isotopic labelling quantitative proteomics revealed 6215 reproducibly identified proteins in mature leaves across the two genotypes and three time points (0, 2 and 4 days of stress). Of these, 987 were differentially expressed due to stress (*cf.* control plants), including 41 proteins that changed significantly in abundance in all stressed plants. Heat shock proteins, late embryogenesis abundant proteins and photosynthesis-related proteins were consistently responsive to stress in both Nipponbare and IAC1131. Remarkably, even after 2 days of stress there were almost six times fewer proteins differentially expressed in IAC1131 than Nipponbare. This contrast in the translational response to multiple stresses is consistent with the known tolerance of IAC1131 to dryland conditions.

## 1. Introduction

Rice is a critical staple food crop feeding nearly half of the people in the world, and with world population expected to increase to more than 9.5 billion by 2050, producing new varieties of rice with high yield and stress tolerance is essential for sustainable rice productivity [1]. The sensitivity of rice plants to soil and atmospheric abiotic stress factors—including salt, drought and temperature stresses—is a major threat to rice yields worldwide [2]. However, natural vegetation and even highly managed crops often encounter multiple stresses simultaneously, commonly as combinations of drought, salinity and non-optimal temperatures [3,4].

We propose that the pattern of translational responses to combined abiotic stresses could differ qualitatively from the responses to individual stresses reported across disparate experimental protocols. A recent review explored the various physiological and molecular acclimation events in stressed plants, distinguishing responses to individual stresses from those ‘shared’ responses that are the subject of the current study [5]. Therefore, we tested the hypothesis that multiple stresses elicit an immediate translational response, which is more than the simple sum of responses to individual stresses [6]. 

Plant stress signaling caused by a combination of multiple abiotic stress includes both primary and secondary signals. Primary signals are activated immediately after stress (within seconds or minutes) and commonly trigger molecular responses to a variety of stresses. These primary signaling events are the initial elements of stress tolerance at the cell level and provide vital clues to acclimation mechanisms in stress-tolerant genotypes. For example, abscisic acid (ABA) plays a central role in response to drought [7], salinity [8] and extreme temperatures [9] and triggers many acclimation responses in plants [10]. However, downstream secondary molecular and metabolic events are vastly diverse and can be peculiar to one or more stress factors [11]. Quantifying these responses can involve a number of steadily improving technologies, including proteomics, transcriptomics and genomics [12,13]. Proteomics is an ideal technology to explore comparative responses to stress when applied to contrasting genotypes and various experimental treatments, revealing new proteins and genes that contribute to abiotic stress tolerance [14,15].

IAC1131 and Nipponbare are two such contrasting rice genotypes known to display differences in their behavior over a 7-day drought treatment [16]. IAC1131 was chosen because it is an upland rice land race from Brazil with a reputation for tolerance to erratic water supply [17,18], while Nipponbare is a low land rice that tolerates flooding but not drought [18,19] or extreme temperature fluctuations. The present study was designed to investigate how the proteomes of these two genotypes behave in the initial stages after multiple abiotic stresses were imposed, thereby testing short-term translational responses to multiple stresses before severe impacts of cell division and reduced leaf expansion were evident [18]. Protein abundances were investigated using Tandem Mass Tags (TMT) isotopically labelled quantitative shotgun proteomics. Targeting protein abundances during a brief but aggressive imposition of drought, salinity, heat and cold allows us to reveal vital clues to the shared stress responses in rice and those that characterize these two highly contrasting genotypes.

## 2. Results

### 2.1. Physiological Responses to Multiple Abiotic Stress

#### 2.1.1. Gas Exchange

**Figure 1:**
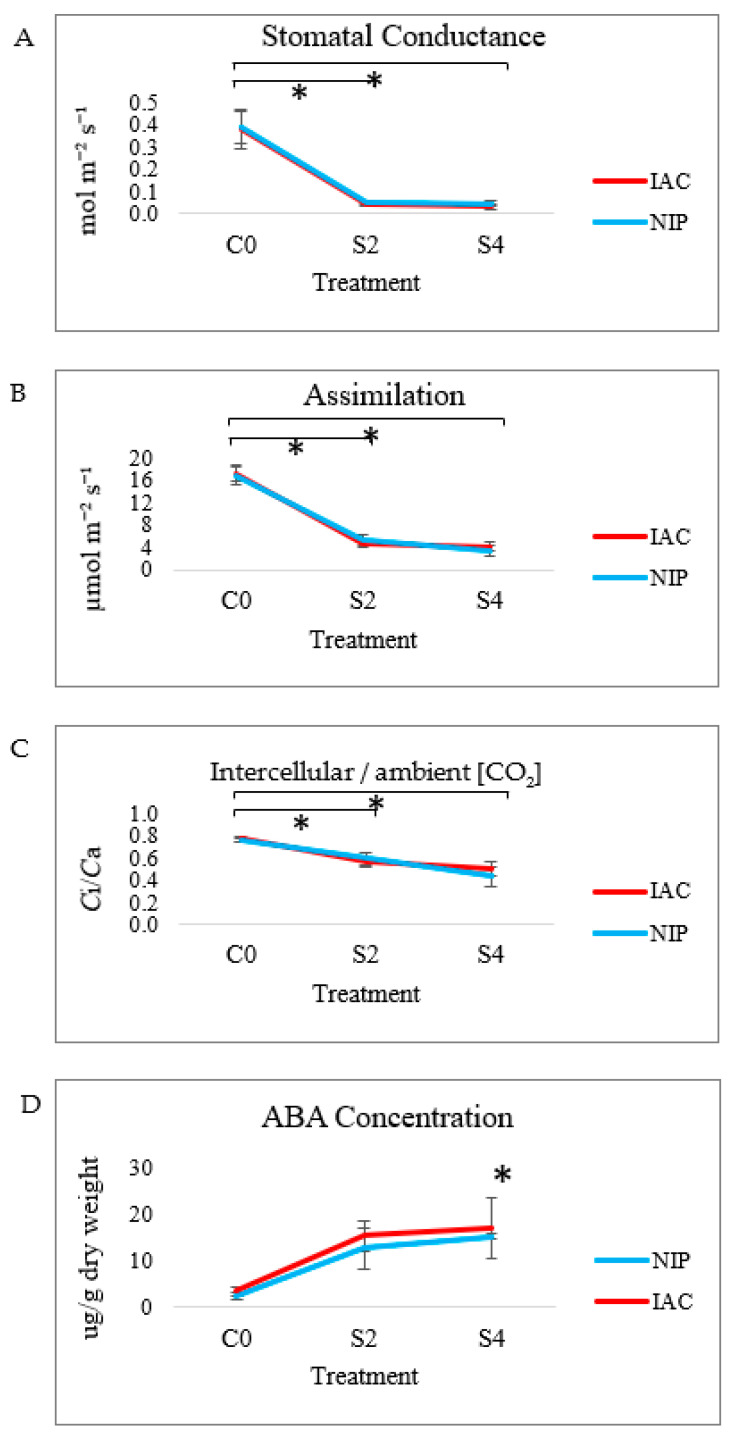
Photosynthetic responses and ABA content of Nipponbare and IAC1131 in response to multiple abiotic stress at three time points (0d = C0, 2d stress = S2 and 4d stress = S4). (**A**) Assimilation (Ar), (**B**) stomatal conductance (gs) (**C**) ratio of intracellular to ambient CO_2_ concentration (Ca/Ci) and (**D**) ABA concentration. Standard errors were obtained from three biological replicate measurements. An asterisk (*) indicates statistically significant difference between the control and stress conditions, according to Student’s *t*-test (*p*-value < 0.05).

Two days after stresses were applied, stomates were almost entirely closed (Figure 1A). This suppressed leaf photosynthetic function, with a 4-fold decrease in Ar and substantial reductions in Ci/Ca (Figure 1B,C). In the first 4d of exposure to multiple stresses, both genotypes behaved similarly, with almost all the negative effects of stress on photosynthesis evident in 2d stress plants.

#### 2.1.2. Abscisic Acid Concentrations

ABA concentration was measured in both Nipponbare and IAC1131 after 2d and 4d exposure to multiple stress treatments. Levels increased rapidly in stressed leaves, rising more than 4-fold in comparison with controls (Figure 1D). Due to variability between biological replicates, only the 4d stress sample in comparison with control condition in IAC1131 was statistically significantly different, but increases in means were consistently recorded once stresses were applied. 

### 2.2. Quantitative Proteomic Analysis of Rice Leaves under Multiple Abiotic Stress

A total of 6215 non-redundant proteins were reproducibly identified and quantified across both IAC1131 and Nipponbare treated with multiple abiotic stress at three time points (0, 2 and 4d stress) at a peptide and protein FDR of less than 1%. Approximately 16% of these (987) were differentially expressed proteins (DEPs) (fold change greater than 1.5 or less than 0.67 and *t*-test *p*-value < 0.05); these will be the focus of further analysis in this study. Details of all DEPs, including identifier, description and fold-change values are presented in Supplementary Table S1.

#### 2.2.1. Differential Response to Multiple Abiotic Stress

**Figure 2:**
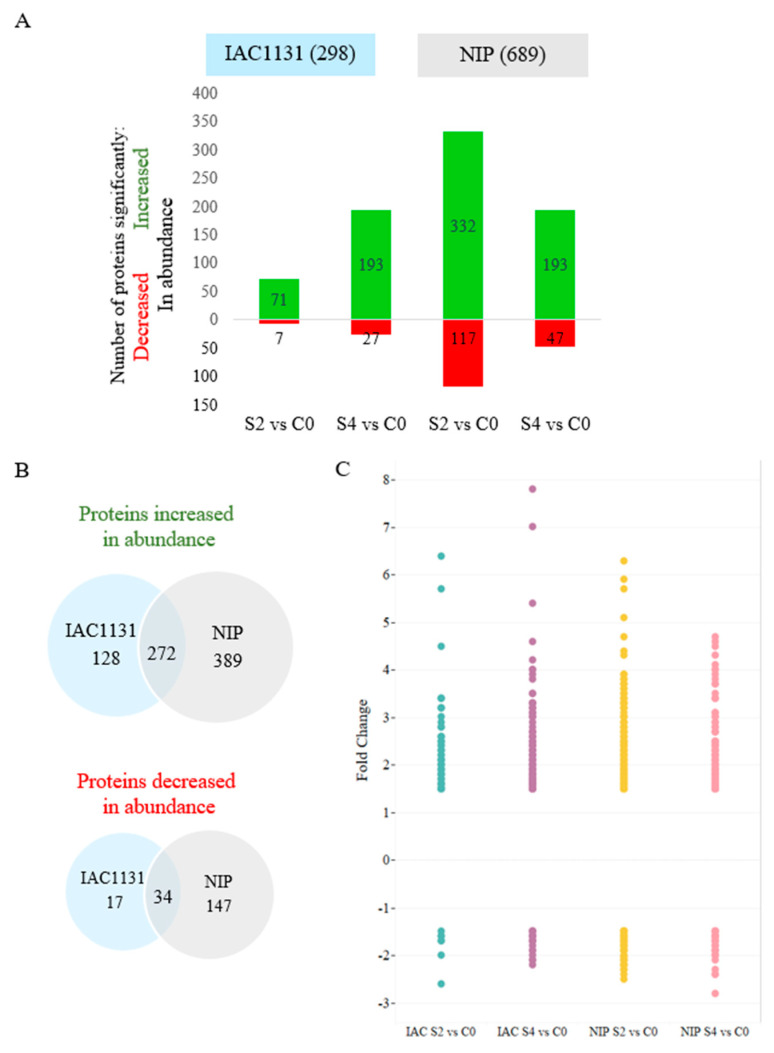
Differential protein expression of two rice genotypes after multiple abiotic stress treatment. (**A**) Number of significantly differentially expressed proteins seen at each time point for each genotype. (**B**) Unique and common proteins significantly increased and decreased in abundance across IAC1131 and Nipponbare. (**C**) Range of fold-change of DEPs in both genotypes after stress treatments.

Quantitative comparisons were undertaken between control (C0) and both time points of stress conditions (S2, S4). A numerical summary of the differentially expressed proteins is presented in Figure 2. Analysis of the proteome response to multiple abiotic stress revealed that approximately double the number of proteins were significantly changed in abundance in Nipponbare (689) in comparison with IAC1131 (298) (Figure 2A). A greater number of DEPs in Nipponbare were identified after 2d stress (449), with 332 proteins increased in abundance and 117 decreased, whilst at 4d stress, there were 193 proteins increased in abundance and 47 decreased. In contrast, IAC1131 stress exposure resulted in a greater number of DEPs after 4d stress (220), with 193 proteins increased in abundance and only 27 decreased. At 2d of stress, only 71 proteins increased in abundance in IAC1131 and seven decreased (Figure 2A). This indicates that changes at the molecular level in response to stress occurred more rapidly in Nipponbare. 

A total of 272 out of 789 proteins (34%) significantly increased in abundance in response to stress were found in both genotypes (Figure 2B). Similarly, 34 of the 198 proteins (17%) that were significantly decreased in abundance in response to stress were also found in both genotypes. The total number of proteins increased in abundance was approximately 4-fold greater than the total number of proteins decreased in abundance, but with respect to differentially abundant proteins common to both genotypes, this ratio almost doubled to nearly 8-fold. Analysis of the range of fold changes for the differentially expressed proteins showed that the highest levels of fold change in individual proteins that were increased in abundance were seen for IAC1131, up to almost 8-fold for late embryogenesis abundant protein 19 (P0C5A4) (Figure 2C). In contrast, the greatest fold-change for a protein decreased in abundance was observed in Nipponbare, with the bi-directional sugar transporter SWEET1A (Q8RZQ8) reduced almost 3-fold. 

#### 2.2.2. Proteome Response across Genotypes and Stress Treatments

**Figure 3:**
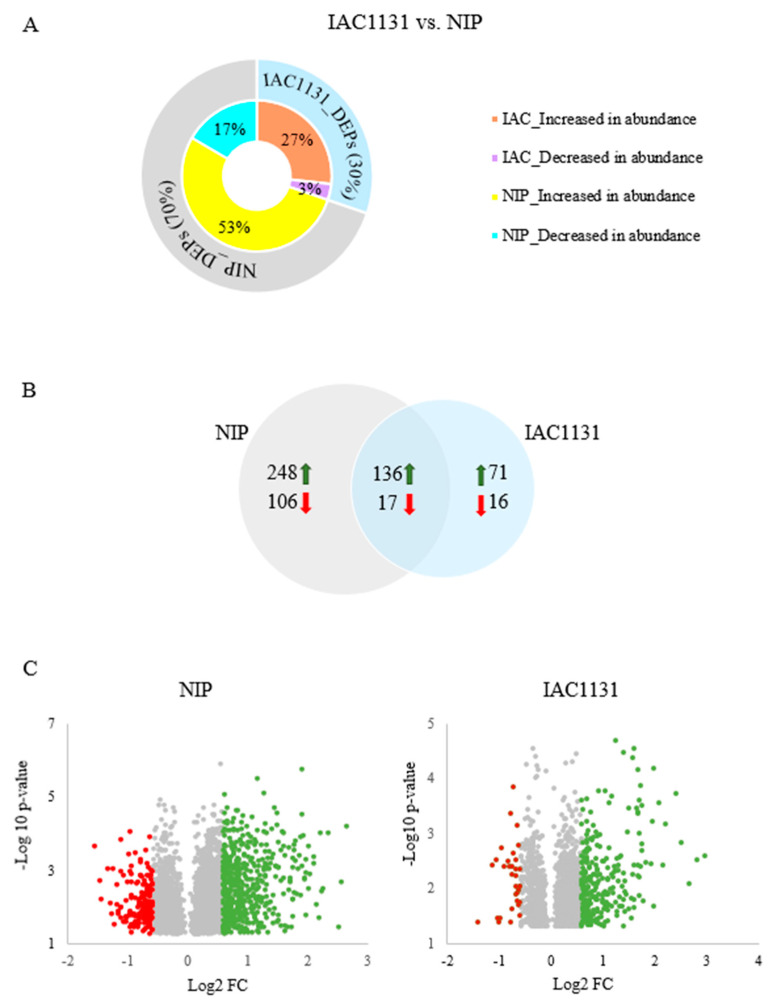
Differential protein expression of two rice genotypes after multiple abiotic stress treatment. (**A**) Polar plot of number of significantly differentially expressed proteins in each variety. (**B**) Unique and common differentially abundant proteins between IAC1131 and Nipponbare. (**C**) Fold change of DEPs in both genotypes after stress treatment.

As shown in Figure 3A, both genotypes dynamically responded to multiple abiotic stresses, with 70% of the redundant total count of 987 DEPs found in Nipponbare and 30% found in IAC1131. A redundant total count allows for the same DEP to be counted more than once if it is found in multiple genotypes or time points. Proteins increased in abundance represent 80% of the total, while those that are decreased in abundance account for only 20% of the total. Figure 3B presents the distribution of the nonredundant total of 594 proteins found to be differentially expressed, with 354 found only in Nipponbare, 87 found only in IAC1131 and 153 found in both genotypes. In this instance, a non-redundant total allows for DEP to be only counted once in each genotype. The preponderance of proteins that increased in abundance in response to stress in both genotypes is also clearly illustrated in the volcano plots of fold change versus *p*-value *t*-test for each variety shown in Figure 3C, which both contain far more data points on the positive side of the log2 fold-change axis. 

**Figure 4:**
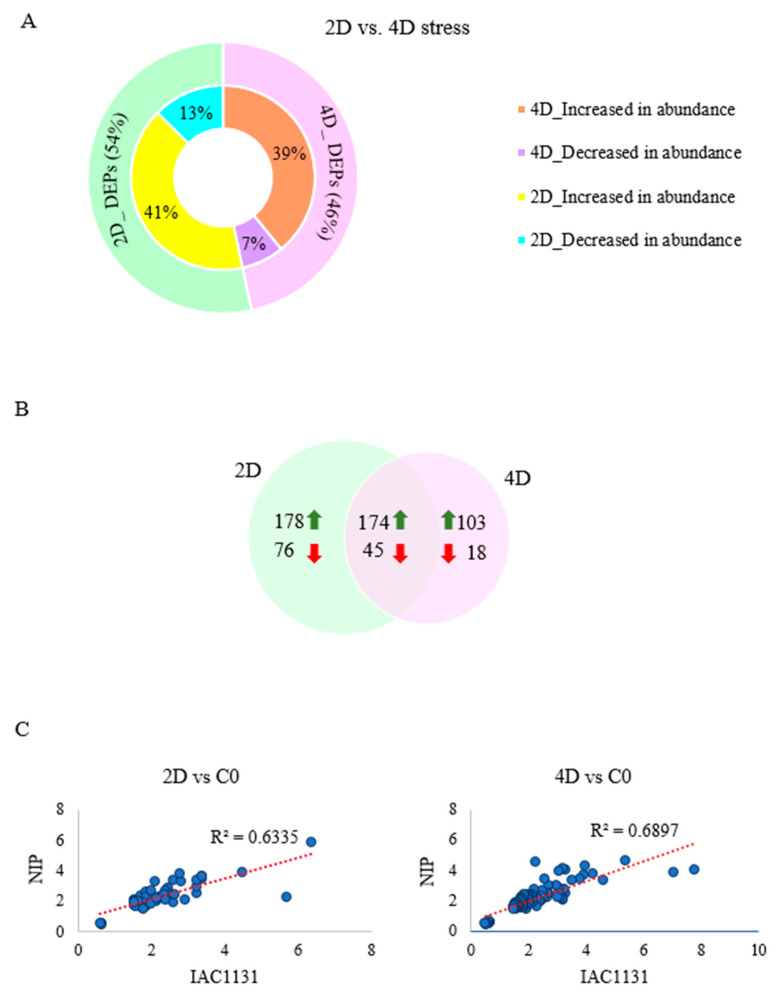
Comparison of differentially expressed proteins at 2d and 4d of multiple abiotic stress. (**A**) Polar plot of number and percentage of significantly differentially expressed proteins at stress each time point. (**B**) Unique and common differentially abundant proteins between the two time points. (**C**) Scatter plots of fold-change values of the differentially expressed proteins found in both varieties at 2d and 4d of stress, measured against control and plotted against each other.

The distribution of the redundant total count of DEPs (987) between the 2d and 4d stress time points as shown in Figure 4A is close to equal, with 54% of DEPs occurring at the 2d stress timepoint and 46% found at 4d stress. It was also observed in the Venn diagram of 594 nonredundant DEPs identified that 219 of the DEPs were found at both the 2d and 4d stress time points (Figure 4B). In this instance, a nonredundant total allows for DEP to be counted only once at each stress time point. There were 254 proteins that changed in abundance only at the two-day time point, while 121 proteins changed in abundance only at the 4d time point. The observed fold-change values of the differentially expressed proteins found in both varieties at 2d and 4d, measured against control, were plotted against each other (Figure 4C). The scatter plots show good linear correlation, with R-squared values of 0.63 and 0.69, indicating that most of the DEPS at both time points were changed in abundance to a similar degree in both varieties. This reflects the fact that the proteomes of both genotypes responded to stress in a qualitatively similar manner, although Nipponbare had a quantitatively distinct translational response to stress, as observed from the large number of differentially abundant proteins reported after only 2d of the three stresses had been applied.

#### 2.2.3. Common Differentially Abundant Proteins

**Figure 5:**
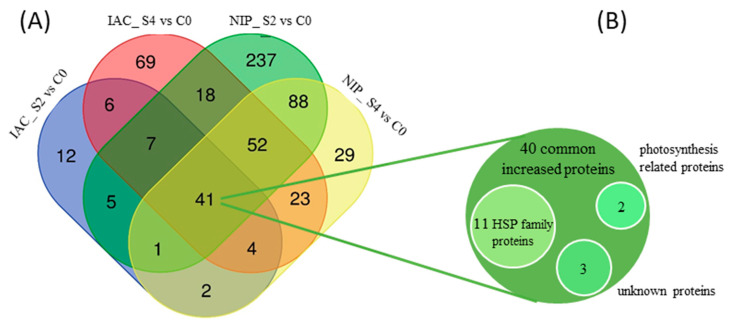
Visualization of differentially abundant proteins identified and quantified under two stress time points in IAC1131 and Nipponbare. (**A**) Venn diagram indicating the overlap in proteins uniquely and commonly identified and quantified in IAC1131 and Nipponbare after 2d and 4d of stress. (**B**) Expanded view of some important protein families represented in the commonly increased in abundance protein category.

A visual representation of the detailed distribution of the nonredundant total of 594 DEPs is shown across two stress treatments (2d and 4d stress) compared with controls for both rice genotypes (Figure 5). The number of uniquely identified DEPs increased greatly in IAC1131 with increasing stress time, while the opposite trend was observed for Nipponbare with fewer unique proteins observed at the longer stress time. 

A total of 41 non-redundant proteins were identified as significantly changed in abundance in all samples, with 40 increasing in abundance and only one protein decreasing. This included 11 heat shock protein (HSP) family members, which are known to be involved in initial response to stress in many cellular systems. The differentially expressed proteins observed in all samples varied in fold change from 7.79-fold (Late embryogenesis abundant protein (LEA19); highest fold change) and 5.36 (Photosystem II 10 kDa polypeptide) to −1.94 (Putative PAP-specific phosphatase; lowest fold change). Putative PAP-specific phosphatase, the only ‘shared’ protein that decreased in abundance in stress, became less abundant between 2d and 4d in both IAC1131 and Nipponbare despite most proteins becoming more abundant over this interval in IAC1131. On the other hand, proteins generally increased in abundance in Nipponbare in the first 2d of stress and changed little in the following 2d. 

**Figure 6:**
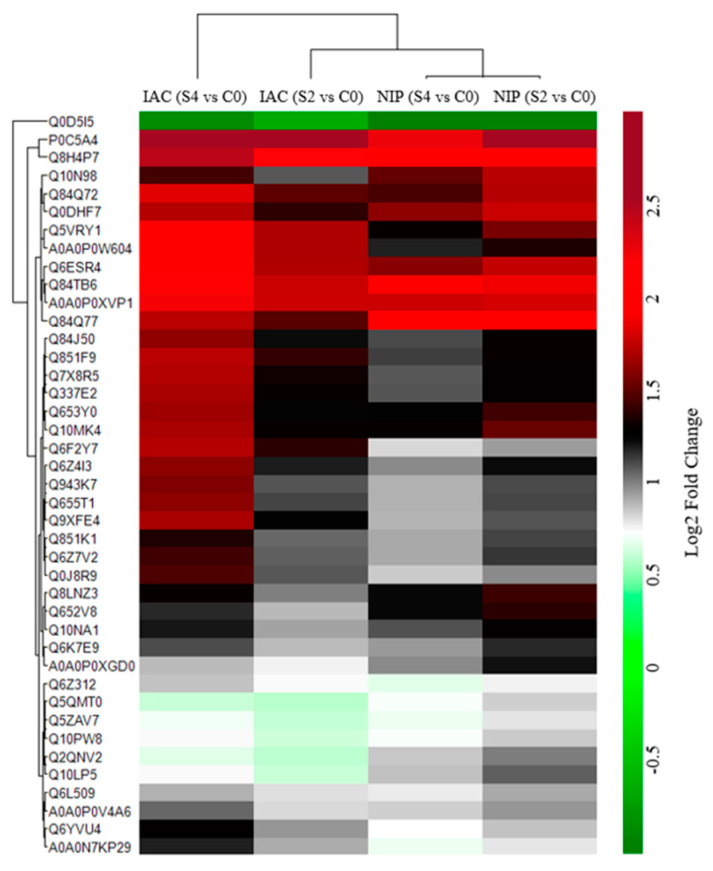
Expression patterns of common DEPs in IAC1131 and Nipponbare after exposure to 2d and 4d multiple abiotic stress. The fold changes of 41 significantly changed proteins were log 2-transformed. and hierarchical clustering was performed using Euclidean as the distance metric and average as the linkage criterion.

A heatmap of the 41 common differentially abundant proteins in all conditions was prepared to investigate the relation between relative fold change of these proteins and genotypic responses to multiple abiotic stress (Figure 6). The data from Nipponbare 2d and 4d stress cluster tightly together, and there was little change after 2d. For IAC1131, the 2d stress treatment revealed a unique group of proteins decreased in abundance, while 4d stress treatment is distinctly different from the other three stress sampling points (NipS2 and S4; IACS2).

**Figure 7:**
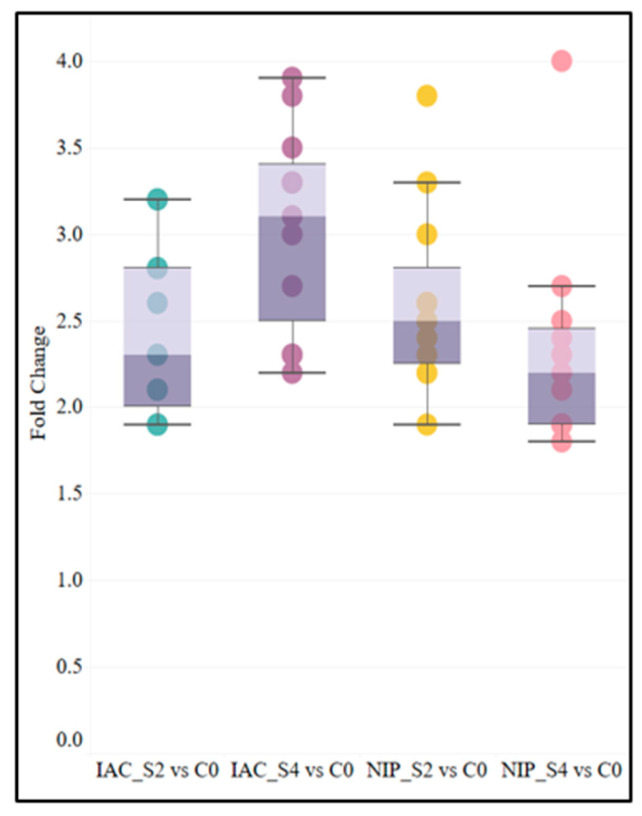
Box and whisker plots showing the spread of relative fold change of 11 HSP family proteins overexpressed in all conditions in both IAC1131 and Nipponbare. The shaded box indicates the interquartile range, while the color boundary within the shaded box indicates the median. The horizontal bars indicating the range of the data show the lesser value of either the minima or maxima or 1.5 times the interquartile range.

More than 25% of the proteins (11/40) that increased in abundance in all samples belonged to the HSP family. These proteins are associated with mechanisms closely related to plants tolerance to stress. These included heat shock protein 82, 18.0 kDa class II heat shock protein (HSP 18.0), 18.1 kDa class I heat shock protein (HSP 18.1), 17.9 kDa class I heat shock protein (HSP 17.9A), Chaperone protein ClpB1, 17.7 kDa class I heat shock protein (HSP 17.7), 70 kDa heat shock protein, 24.1 kDa heat shock protein (HSP 24.1), Heat shock cognate 70 kDa protein, 16.0 kDa heat shock protein (HSP 16.0) and 18.6 kDa class III heat shock protein. The fold change values of the eleven common differentially expressed HSPs are shown in Figure 7. The minimum FC number is similar for both IAC1131 and Nipponbare under 2d of stress, while the median and maximum for IAC1131 increased to a higher level after 4d of stress during which the median for Nipponbare decreased noticeably. 

#### 2.2.4. Proteins of Unknown Function Found to Be Differentially Abundant in All Conditions

Interestingly, of the 41 common differentially expressed proteins identified in both Nipponbare and IAC1131 in response to multiple abiotic stress treatments after 2d and 4d, three proteins were observed with no reported specific function. Q0DHF7 was 77.5% homologous with PEBP (Phosphatidylethanolamine-binding protein) family protein (*Zea mays*), A0A0P0V4A6 was 63.6% homologous with ZmGR2c protein (*Zea mays*) and A0A0P0XGD0 was 51.4% homologous with voltage-dependent L-type calcium channel subunit (*Parasponia andersonii*). 

Q0DHF7 was most strongly induced, increasing in abundance over time in IAC1131, with fold change increasing from 2.5 at 2d stress to 3.3 at 4d stress and decreasing in abundance over time in Nipponbare with fold-change values of 3.3 at 2d stress and 3.0 at 4d stress. A0A0P0V4A6 and A0A0P0XGD0 were both induced by 1.5–2.2 fold and showed a similar pattern in that they were increased in abundance over time in IAC1131 and decreased in abundance over time in Nipponbare. Our results indicate that all three of these proteins may potentially play important roles in tolerance to multiple abiotic stress in rice, and further studies are needed to determine their functions.

### 2.3. Functional Enrichment Analysis of Proteins Significantly Changed in Response to Stress

Functional annotation and enrichment analysis of differentially expressed proteins were performed for both genotypes under both treatments. The results of this analysis are presented graphically in Supplementary Figure S1. Using REVIGO analysis of the top 10 enriched gene ontologies (GO) generated from plants response to acute multiple abiotic stress, bubble plots were created, representing proteins increased and decreased in abundance in Nipponbare and IAC1131, at 2d and 4d stress. No significant enrichment was found for the small number of proteins decreased in abundance in IAC1131 under 2d stress.

Gene Ontology enrichment analysis showed that the proteins commonly increased in abundance that were enriched after stress exposure were involved in the ‘unfolded protein binding,’ ‘misfolded protein binding,’ ‘identical protein binding’ and ‘protein folding chaperone’ molecular function categories. The increased abundance of these gene categories in multiple comparisons suggests that these functional categories may be important in the plant response to environmental stresses. Each combination of treatment and cultivar also showed GO categories specifically enriched, such as ‘heat shock protein binding,’ which was exclusively observed in IAC1131 after 4d stress. 

Among proteins commonly decreased in abundance, those involved in ‘transferase activity’ categories were significantly enriched in both IAC1131 and Nipponbare in response to 4d stress. In contrast, 2d stress in Nipponbare caused a decrease in the abundance of the oxidoreductase and protochlorophyllide reductase categories. The ‘mRNA binding protein’ category was also enriched in proteins that were decreased in abundance in Nipponbare after the 2d stress treatment, suggesting that stress response may also impact upon post-transcriptional processing and modification of RNA in the multiple abiotic stress response.

## 3. Discussion

The proteomes of mature leaf tissues revealed highly distinctive changes over the four days following imposition of multiple abiotic stresses in two rice genotypes chosen for their contrasting responses to stress. Importantly, no visible symptoms of the combined stress such as wilting or chlorosis were observed over the 4d treatment. Stress settings (50 mM NaCl; 50% FC; 33 °C/18 °C) were chosen following an earlier calibration experiment in which the impact of each stress had been assessed separately to broadly equate their individual impacts on the seedlings. The precision achieved by using this protocol enabled insights into the molecular events triggered in the first stages of complex stresses when critical acclimation events would be expected to occur [20,21]. The short-term plant responses to stress that characterize the earliest stages of acclimation are a priority for developing resilience in all major cereal crops, particularly as more challenging environmental conditions are amplified [3,5,22]. Our hypothesis is that the proteins expressed in response to combined stress—the so-called ‘shared’ stress proteins—could lead researchers to valuable stress markers that have co-evolved as common responses to multiple stresses [5].

Ultimately, abiotic stresses affect physiological, morphological and molecular processes according to the severity and duration of the stress events. However, the rapidity of impairment depends upon acclimation and tolerance mechanisms, prompting us to use a rice genotype (IAC1131) that is reputedly well adapted to dry conditions and, thus, would be expected to have protein expression patterns that reflect its capacity to acclimate. The molecular basis of acclimation in IAC1131 should become apparent in the first days after stress is imposed, as part of a suite of primary stress responses [20]. According to this rationale, we examined the response of two contrasting rice genotypes to four days of combined drought/salinity/thermal stress, enabling the quantification of the abundance of thousands of differentially expressed proteins.

Comparative responses of Nipponbare and IAC1131 to multiple abiotic stress were assessed on two levels. They were first characterized physiologically to establish the scale of stress response, then at the proteomic level to identify differentially expressed proteins. Two days of abiotic stresses caused a rapid rise in leaf ABA concentrations, accompanied by rapid stomatal closure, reduced intracellular CO2 concentrations and almost total cessation of CO2 assimilation, all of which are consistent with previous studies [23,24]. The initial physiological response to stress was statistically identical in Nipponbare and IAC1131 after four days and caused no symptoms of damage to the plants, thus enabling the identification of acclimation events at the translational (proteomic) level that give rise to the superior phenotype of IAC1131 we have previously observed after longer periods of gradually imposed drought stress [16,18,25].

At the proteome level, significantly more differentially expressed proteins under stress conditions in Nipponbare indicate a more sensitive response to stress, corresponding to previous observations of greater disturbance in cellular homeostasis in stress-sensitive plants [26]. Other reports have suggested that the proteome response of plants can be characterized into a series of dynamic proteome response phases including alarm, acclimation and long-term resistance under unfavorable conditions [27]. The initial alarm phase causes cellular homeostasis to enter a phase of disequilibrium, including stress-responsive signaling and disruption to steady-state gene expression patterns. Inherent differences in stress tolerance become evident in the alarm phase, with sensitive genotypes characterized by severe disruption to homeostasis, in contrast with resistant genotypes that acclimate to adverse environments by the expression of both well-known and novel stress-responsive proteins [28]. That is, proteins are expressed differentially in response to stress, even within phenotypically contrasting genotypes of a single species. However, some proteins are altered in abundance in response to abiotic stress in all plants, dependent on the level and duration of the environmental triggers [29,30].

Proteome profile quantitation in this study resulted in the identification of 689 DEPs in Nipponbare and 298 DEPs in IAC1131, reflecting their previously established reputations for tolerance to abiotic stresses; Nipponbare is moderately salt-tolerant and sensitive to drought, while IAC1131 is a stress-tolerant genotype. This suggests that Nipponbare may respond to multiple abiotic stress more dynamically than IAC1131, observed as a more expansive set of differentially expressed proteins across more functionally relevant pathways. This is similar to a related study of cold stress in two potato species, which reported a greater number of significantly differentially expressed proteins under cold treatment in common potato plants in comparison with a frost-tolerant species [31]. 

In addition to genotypic differentiation, patterns of changes in protein abundance between two and four days of stress were instructive. For example, a surge of differentially expressed proteins in Nipponbare within just two days of application of multiple stresses contrasted with the trend in IAC1131, where more proteins responded four days after multiple stress treatment than after two days. Notably, a large number of proteins expressed under stress conditions were cultivar-specific, while others were specific to each time point (Figure 4 and Figure 5). Again, this result indicates the higher tolerance of IAC1131 to multiple abiotic stress in the alarm phase. Typically, the number of DEPs can be considered to represent the level of sensitivity or tolerance to induced stress, with stress-sensitive genotypes likely to display more differentially abundant proteins, with some more abundant but others less abundant under stress. 

Accumulated evidence shows that ABA interacts with receptors to trigger a diversity of phenotypic responses under abiotic stress [32,33]. One protein that can potentially be activated indirectly by changes in ABA concentration is bZIP transcription factor 23. We showed that this protein was more abundant under all stress treatments in Nipponbare and IAC1131, with the fold change level similar for both genotypes. It has been found previously that bZIP proteins play an active role in ABA signaling in plants [34], and their increasing abundance is directly correlated with higher tolerance to abiotic stresses in plants [35,36,37,38,39].

Two subunits of the photosystem II protein family, photosystem II 10 kDa polypeptide (PsbS) and photosystem II 22 kDa protein 2, were commonly increased in abundance under all stress treatments. Recent studies in transgenic tobacco showed an association between overexpression of PsbS and reduced stomatal opening, reducing transpiration [40]. In the current study, we also report ABA accumulation, stomatal closure and PsbS becoming more abundant. Other studies have shown that Photosystem II proteins play a potential role in abiotic stress tolerance in plants [41]. In our results, the abundance of both of these proteins increased from 2d to 4d in both Nipponbare and IAC1131, although the increase was greater in IAC1131. Another related photosynthesis protein is ATP-dependent zinc metalloprotease, a molecular chaperone that has been previously identified in salt-tolerance studies in chickpea [41,42]. Our results show that this protein increased in IAC1131 under both treatment conditions, while it only increased after 4d of stress in Nipponbare. These time courses provide clues to protein expression events that might play a core role in abiotic stress tolerance. 

Aside from increasing the expression of photosystem II protein family members, multiple abiotic stress resulted in an increase in abundance of Late Embryogenesis Abundant 19 protein (LEA); this protein showed the largest change in abundance of those DEPs common to two stress time points and genotypes. LEA proteins induced under stress conditions play a role in stress tolerance by producing essential metabolic proteins and also function as stress signaling factors involved in further signal transduction and gene expression [43]. Overexpression of LEA genes directly relates to abiotic stress tolerance in many plants [38,44]. Interestingly, recent studies have shown that LEA proteins decrease lipid oxidation by enhancing photosynthetic activity in plants [45]. Hence, we postulate that higher LEA protein activity could have a functional link with the upregulation of PS II 10 kDa polypeptide protein in terms of enhancing the stress tolerance of the plants.

Among the molecular functions related to proteins that were decreased in abundance in response to stress, oxidoreductase and protochlorophyllide reductase, which are known to play a very important role in stress response in plants [46], were enriched in Nipponbare after two days of stress exposure. This function is also related to photosynthesis and correlates with previous findings that, by overexpression of defense-related proteins in plants, photosynthetic proteins decline [47]. 

Additionally, ATPase was a vital function that was enriched only in NIP in response to multiple abiotic stress, which may be related to the significant role of ATPase in plants in responding to oxidative stress response [48]. ATPases are constitutively expressed and have been shown to participate in ion homeostasis in plant cells, and various types of ATPase have been found to be differentially expressed in response to salt stress at different levels [47,49]. 

The widely documented role played by HSPs in the response of plants to diverse stresses was reinforced in our study in that, among the proteins common to both stress terms and genotypes, HSPs constituted a significant number of the DEPs. Aside from constitutively increased levels of several stress-protective proteins in tolerant genotypes, stress-induced increases in some common stress-responsive proteins such as HSP70 and thioredoxin h have been found in genotypes with contrasting levels of tolerance to stress [50]. Heat shock proteins (HSPs), which are a large family of critically important molecular chaperones, are central in stress-responsive signal transduction that enables plants to mitigate the adverse effects of environmental stressors [51,52]. Heat shock transcription factors (HSFs) are the main factor resulting in the overexpression of HSPs in several abiotic stress responses [53,54]. HSP family proteins play a key role in plants adaptation and acclimation to abiotic stress and their higher accumulation in IAC1131 at 4d of stress highlights again the enhanced tolerance to stress of this genotype. 

Interestingly, 11 out of 40 (>25%) of the common proteins increased in abundance in both stress terms and genotypes belonging to the HSP family, in addition to one chaperone protein, ClpB1. All of these are known to function as molecular chaperones involved in protein folding in response to negative effects of plant stress [55,56]. Chaperones actively participate in protein quality control to maintain cellular homeostasis in induced stress conditions [57]. Based on the measured protein fold-change level, all of the previously mentioned proteins in HSP and chaperonin groups showed opposite trends in the two genotypes under two stress time points. All of these HSPs and chaperone proteins a higher accumulated more after 4d of stress treatment in comparison with 2d stress in IAC1131, while, in contrast, in Nipponbare, almost all of these proteins were less expressed after 4d compared with 2d of multiple abiotic stresses. The explanation of these differences may also be that stress susceptible genotypes such as Nipponbare are able to induce expression of stress responsive proteins faster in the initial alarm phase of response to stress but did not sustain it in the subsequent acclimation phase.

Notably, the only protein in response to all treatments that commonly decreased in abundance was phosphoadenosine 5′-phosphate-specific phosphatase (PAP). Plant PAPs also display non-specific acid phosphatase (ATPase) activity, hydrolyzing different organic phosphate compounds in plants such as rice [58]. Reversible protein phosphorylation mediated by protein phosphatases is one adaptive cellular response used to maintain a critical balance in phospho-regulation during normal and adverse growth plant conditions. Moreover, protein phosphatases are known to mediate abiotic stress-triggered signaling pathways [59]. ABA negatively correlates with the regulation of a group of major phosphatases [60,61]. Therefore, decreased abundance of PAP-specific phosphatase in both Nipponbare and IAC1131 could be interpreted as a consequence of increasing ABA production occurring in response to multiple abiotic stress.

Functional studies have complemented proteome analysis, particularly under stress treatment, in developing our understanding of protein metabolic pathways in plants [62]. Metabolic network alteration reflects the diversity and versatility of plant species response when subjected to unfavorable conditions [63,64]. Our results indicated that molecular functions associated with protein unfolding, protein misfolding and protein folding chaperones were the main mechanisms enriched in common with two stress time points and genotypes. 

Heat shock protein binding, which is usually associated with reducing oxidative damage [65], was one of the enriched molecular functions in both IAC1131 and Nipponbare after 4d stress, while chaperone binding was exclusively enhanced in Nipponbare exposed to 2d stress. Generally, chaperones are known as stress proteins responsible for avoiding protein aggregation under stress conditions. However, chaperones can also play a constitutive role in protein folding, assembly, stabilization, translocation and degradation [66,67]. It has been shown previously that protein misfolding or protein unfolding can be a consequence of environmental cues [68], which results in the overexpression of chaperonins and a group of proteins equilibrating between protein-folding demands and capacity. There have also been reports that heat stress results in the binding of misfolded proteins associated with releasing heat-stress transcription factors from the chaperones and, thus, activates heat stress responses [69,70].

Nipponbare is considered to be a cultivar that is sensitive to a range of abiotic stresses. Notably, its rapid translational response to multiple abiotic stresses, compared with the more tolerant IAC1131, is consistent with previous observations on stress-sensitive plants. Many redundant DEPs were common to both genotypes but most non-redundant DEPs were characteristic of Nipponbare. In that, we have reported molecular responses to stress within two days of multiple stress, and we claim that many proteins represent primary responses that constitute part of a complex of acclimation events. 

## 4. Materials and Methods

### 4.1. Plant Material and Stress Treatment

After soaking for 5 min in water, seeds were sterilized in four steps: rinsing in 70% ethanol for 1 min followed by water for 1 min, 50% bleach for 20 min and finally in water for 5 min. Sterilized seeds of rice (*Oryza sativa*) genotypes, IAC1131 and Nipponbare were sown in similar pots containing one plant per pot (30 cm deep, 10 cm in diameter, filled with 700 g soil). There were 30 pots in total, with 15 for each genotype and five for each treatment. Plants were grown in a greenhouse with the temperature set to 28/22 °C (day/night) and a 12 h photoperiod. Natural light was supplemented with red/blue LED lights (Philips GreenPower LED top lighting module DR/B HB 400V, Philips, Epping, NSW, Australia) to maintain a minimum of 600 µmol m^−2^ s^−1^ throughout. 

After four weeks of growth under control conditions, plants at the 5-leaf stage were subjected to a multiple stress regime. Ultimately, stressed plants were exposed to 50% Field Capacity (FC) (drought stress), 50 mM NaCl (salt stress) and 33/18 °C (temperature stress). To achieve these stresses simultaneously, two days before starting the stress treatment, pots were watered to 100% FC with 25 mM NaCl. Water was withheld until FC fell to 50% FC (50 mM NaCl), largely through evaporation. Daily water loss was recorded daily by weighing pots. Once soil water and salt levels reached their target values, the multiple abiotic stress treatment was started by altering glasshouse temperatures to 33/18 °C (day/night) and maintaining watering at 50% FC with fresh water in order to not allow salt levels to rise above 50 mM. After 0 days (control) and 2 and 4 days (stress) (hereafter referred to as 0d, 2d and 4d), freshly harvested mature leaves were used for measuring physiological parameters and then collected from three biological replicate groups of five plants each and frozen immediately in liquid nitrogen. An additional five plants of each genotype were maintained at control conditions for the duration of the four-day stress period and included in the subsequent proteomic analysis as additional controls. The 40 pots initially sown, comprising 20 of each genotype, were thus divided into eight sampling groups of five pots each, labelled as Nip C0 (control 0d), IAC C0 (control 0d), Nip S2 (stress 2d), IAC S2 (stress 2d), Nip S4 (stress 4d), IAC S4 (stress 4d), Nip C4 (control 4d) and IAC C4 (control 4d). 

### 4.2. Physiological Measurements

#### 4.2.1. Gas Exchange Parameters

At three time points of 0d, and 2d and 4d of multiple abiotic stress, CO2 exchange variables were measured with a LICOR photosynthesis system (LI-6400, LI-COR, Inc., Lincoln, NE, USA). Variables assessed were assimilation rate (Ar), transpiration rate (Er), stomatal conductance (gs) and the ratio of intercellular to ambient CO2 concentration (Ci/Ca) measured on the youngest fully expanded leaves, with at least three biological replicates measured at midday. Parameters included CO2 concentration of 400 µmol m^−1^, photosynthesis active radiation (PAR) at 1800 µmol m^−2^ s^−1^, relative humidity (RH) at 50% and temperature of leaf chamber adjusted to 30 °C and 33 °C for control and stressed plants, respectively. The results presented are the means ± standard error of three replications. 

#### 4.2.2. Abscisic Acid (ABA) Assay

An ELISA (enzyme-linked immunosorbent assay) assay kit was used to measure assessed ABA levels according to the manufacturer’s instructions (Biomatik, Berkeley, CA, USA). Finely ground freeze-dried leaf material measuring 0.1 g was weighed out into a centrifuge tube containing 0.9 mL of extraction buffer. The samples were shaken overnight at 4 °C in the dark. The solids were centrifuged, the supernatant was diluted 1:1 with H_2_O and ELISA was performed in a 96-well microplate and measured using a microplate reader (Thermo Fisher Scientific, San Jose, CA, USA) set to 450 nm. The results are the means ± SE of three replications. 

### 4.3. Proteome Quantification and Analysis

#### 4.3.1. Protein Extraction and Assay

Frozen leaf samples were ground finely in liquid nitrogen using a Qiagen ((Germantown, MD, USA) Retsch 12090 TissueLyser II with Zironox beads (2.8–3.3 mm). Leaf powder measuring 50 mg was suspended in 1.5 mL of 10% trichloroacetic acid in acetone, 0.07% β-mercaptoethanol and incubated at −20 °C for 45 min. The extract was centrifuged for 15 min at 16,000× *g* at 4 °C, and the pellet was collected and washed with 1.5 mL of 100% acetone followed by centrifugation for 15 min at 16,000× *g* at 4 °C. The acetone washing step was repeated three times to remove pigments, lipids and other lipophilic molecules. The resulting colorless pellet was lyophilized in a vacuum centrifuge for 5 min, and 400 µL of 2% SDS in 50 mM Tris-HCl (pH 8.8) was used to resuspend the pellet. After shaking for 2 h, the pellet was centrifuged and the supernatant reduced by adding 1 M dithiothreitol to reach a final concentration of 10 mM and incubated for 1 h at 37 °C, followed by alkylation with 20 mM iodoacetamide for 45 min in the dark at room temperature. Samples were then methanol-chloroform precipitated. The amount of 300 µL of protein solution was mixed with 800 µL of methanol and 200 µL of chloroform. The amount of 500 µL of water was added to the mixture, vortexed and centrifuged at 6000× *g* for 2 min. After removing the upper phase, 600 µL of methanol was added to the mixture and centrifuged at 6000× *g* for 2 min, and the supernatant was removed. The pellet was air-dried and solubilized in 80 µL of 8 M urea in 100 mM Tris-HCl buffer (pH 8.8). The concentration of protein in the solution was measured by bicinchoninic acid (BCA) protein assay kit (Pierce TM, Thermo Fisher Scientific, San Jose, CA, USA). 

#### 4.3.2. Trypsin In-Solution Digestion and Peptide Extraction

The amount of 200 µg of protein was used for digestion and peptide extraction. Samples were diluted five times with Tris-HCl 100 mM buffer (pH 8.0) to reduce urea concentration to less than 2 M. For peptide digestion, trypsin (1:50 enzyme to protein) was added to samples and incubated overnight at 37 °C. The reaction was stopped by adding trifluoroacetic acid (TFA) to reach a final concentration of 1% and samples were desalted using an SDB-RPS (3M-Empore) stage-tip (SDB-RPS, 3M, Saint Paul, MN, USA) consisting of 4 punches of SDB membrane in 200 µL pipette tips, centrifuged at 2500 rpm. The tips were washed two times with 200 µL of 0.2% TFA and eluted by the addition of 200 µL each of 80% acetonitrile (ACN) and 5% NH4OH followed by centrifugation at 2500 rpm for 10 min. Digested samples were dried in a vacuum centrifuge and resuspended in 60 µL of 20 mM HEPES buffer (pH 8.0). The peptide concentration was measured using a microBCA kit (Pierce TM, Thermo Fisher Scientific, San Jose, CA, USA). 

#### 4.3.3. Tandem Mass Tag (TMT) Labelling and Fractionation

The amount of 50 µg peptide samples was aliquoted for labeling with 10-plex TMT label reagents (Thermo, San Jose, CA, USA). The amount of 85 µL of ACN was added to each 0.8 mg label vial and mixed well. Ten TMT labels, with respective reporter ions at m/z = 126, 127N, 127C, 128N, 128C, 129N, 129C, 130N, 130C and 131, were added to ten samples matching with Nip C0 (control 0d), IAC C0 (control 0d), Nip S2 (stress 2d), IAC S2 (stress 2d), Nip S4 (stress 4d), IAC S4 (stress 4d), Nip C4 (control 4d), IAC C4 (control 4d), repeat of Nip S4 (stress 4d) and a pooled internal standard. Samples were incubated at room temperature for 1 h to perform labeling, and 8 µL of 5% hydroxylamine was added to each for incubation at room temperature for 15 min. The labeled aliquots were combined and evaporated to dryness in a vacuum centrifuge. Samples were pooled and reconstituted in 1 mL of 0.1% formic acid, desalted by solid-phase extraction and dried again in a vacuum centrifuge followed by reconstitution in 0.1% formic acid. 

The TMT labeled peptides were fractionated using high-pressure strong cation exchange chromatography on a PolyLC PolySulfoethyl A column (200 mm × 2.1 mm, 5 µm, 200 Å) with 210 nm UV detection. Samples were loaded using buffer A (5 mM KH2PO4, pH 2.7, dissolved in Milli-Q water) and fractionated with a linear gradient of 10–45% buffer B (5 mM KH2PO4, pH 2.7, 350 mM KCl and 90% ACN) over 110 min, then 45–100% buffer B over 10 min at a flow rate of 300 µL/min. A total of 96 fractions was collected and combined to produce 17 fractions based on UV absorbance. Fractions were desalted using SDB-RPS (3M-Empore) stage-tips, evaporated in a vacuum centrifuge and reconstituted in 0.1% formic acid in preparation for nano-flow liquid chromatography—tandem mass spectrometry (nanoLC-MS/MS).

#### 4.3.4. Nano LC-MS/MS

Samples were analyzed on a Q Exactive Orbitrap mass spectrometer (Thermo Fisher Scientific, San Jose, CA, USA) coupled to an Easy-nLC 1000 nano-flow HPLC system (Thermo Fisher Scientific, San Jose, CA, USA). Reversed-phase chromatographic separation was carried out on a column of 75 µm internal diameter packed in-house to 10 cm with ES-C18 Halo, 2.7 µm bead size, 160 Å pore size (Advanced Materials Technology, Wilmington, DE, USA). Peptides were fractionated using a gradient starting with solvent A (0.1% formic acid (FA)), then 0–30% solvent B (99.9% acetonitrile/0.1% FA) over 100 min and then 30% to 85% over 10 min. The mass spectrometer was operated in data-dependent mode to automatically switch between Orbitrap MS and ion trap MS/MS. One full MS scan over the scan range of 350 to 1850 m/z for fragmentation was acquired in the Orbitrap at a resolution of 70,000 after accumulation to an automated gain control (AGC) target value of 1 × 10^6^ ions. The ten most abundant ions were selected for higher-energy collisional dissociation (HCD) fragmentation at a normalized collision energy of 35%. The maximum injection time for target ions selected for MS/MS was set to 90 s. The lock mass option was enabled using the polydimethylcyclosiloxane ion (m/z 445.12003) as an internal calibrant for accurate mass measurement. The mass spectrometry proteomics data have been deposited to the ProteomeXchange Consortium [71] via the PRIDE partner repository [72] with the dataset identifier PXD030428.

#### 4.3.5. Peptide to Spectrum Matching

Proteome Discoverer v2.1 (Thermo Fisher Scientific, San Jose, CA, USA) with the Mascot algorithm was used for the peptide to spectrum matching. FASTA files of protein sequences from *Oryza sativa* were downloaded from NCBI as of June 2020, containing 32,367 sequences, and MS/MS spectra were searched against this database, supplemented with common laboratory contaminants. The MS tolerance was set to ± 10 ppm and MS/MS tolerance to 0.1 Da, and trypsin digestion was specified with one missed cleavage allowed. Carbamidomethylations of cysteine and 10-plex TMT tags on lysine residues and peptide N-termini were set as static modifications. Oxidation of methionine and deamidation of asparagine and glutamine residues were set as variable modifications. Search result filters were selected as follows: Only peptides with a Mascot score > 15 and below the significance threshold filter of *p* = 0.05 were included, and single peptide identifications required a score equal to or above the Mascot identity threshold. The false discovery rate was set to 0.01 or less in Proteome Discoverer, and protein grouping was enabled such that when a set of peptides in one protein were equal to or wholly contained within the set of peptides of another protein, the two proteins were contained together in one protein group. Relative quantitation of peptides and proteins was achieved by pairwise comparison of TMT reporter ion intensities after normalizing to the pooled internal standard.

#### 4.3.6. Analysis of Quantitative Proteomics TMT Data

TMTPrepPro [73] was used for further analysis of identified proteins. All protein ratios relative to the reference (label-131) were extracted. The differentially expressed proteins (DEPs) were identified using two-way analysis of variance (ANOVA) of all proteins identified and quantified reproducibly across different treatments, including control, with a *p*-value less than 0.05. For pairwise comparisons of interest, differentially expressed proteins were identified based on *t*-tests of log-transformed ratios. The overall fold changes were calculated as geometric means of the respective ratios. Two criteria were applied to determine significantly differentially abundant proteins: fold change over 1.5 or less than 0.67 and *p*-value less than 0.05. Gene Ontology (GO) information was used to categorize the biological processes of differentially expressed proteins using PloGo [74]. GO IDs were extracted from the UniProt database and matched with the Oryza sativa protein sequence database from NCBI.

#### 4.3.7. Gene Ontology (GO) Functional Enrichment Analysis

In order to elucidate the putative biological functions or molecular mechanisms of the differentially expressed proteins (DEPs), OmicsBox (ver. 2.0.10) was used to perform functional analysis. Fisher’s Exact Test was used to compare the overrepresented cellular processes GO terms in the group of increased and decreased proteins against the Oryza sativa annotated database. GO terms with an uncorrected *p*-value < 0.05 were considered significantly enriched. The lists of the ten highest enriched GO were summarized with REVIGO (http://revigo.irg.hr, accessed on 14 January 2022) by SimRel clustering based on molecular function categories [75].

## 5. Conclusions

Rice is an essential plant that plays a crucial role in food security, and it is under threat from increasing global environmental stresses. However, few studies have been performed to study the contrasting responses of rice genotypes to multiple abiotic stresses and how they might instruct breeders in finding new selection targets. Multiple abiotic stresses significantly impaired photosynthesis in both IAC1131 and Nipponbare within 2d, causing rapid ABA accumulation and stomatal closure. Proteomic expression patterns reflected physiological observations. 

A greater number of stress-induced proteins were identified in Nipponbare when compared with IAC1131. Additionally, patterns of DEPs differed substantially between Nipponbare and IAC1131, with the former appearing to mount the strongest translational response immediately after stresses were applied. In contrast, we propose that in IAC1131 the increasing number of DEPs between 2 and 4d after stresses commenced reflects its inherently higher levels of tolerance. Stress-responsive proteins such as LEA, HSP and PS-II proteins were found commonly altered in abundance in both genotypes, suggesting that they are part of a core stress response that occurs in addition to responses specific to each cultivar. In addition, we identified three uncharacterized proteins with no annotated function, which showed increased accumulation in all plants exposed to stress. These represent potentially novel stress response proteins and deserve further detailed investigation.

## Data Availability

Mass spectrometry proteomics data have been deposited to the ProteomeXchange Consortium [71] via the PRIDE partner repository [72] with dataset identifier PXD030428.

## Supplementary Materials

The following supporting information can be downloaded at https://www.mdpi.com/article/10.3390/ijms23031739/s1.
