# Supplementary material for: Multiple Abiotic Stresses Applied Simultaneously Elicit Distinct Responses in Two Contrasting Rice Cultivars
Source: Int J Mol Sci. 2022 Feb 3;23(3):1739. doi: 10.3390/ijms23031739 (PMC8836074; doi:10.3390/ijms23031739)
Supplement: Supplementary file 1 [file ijms-23-01739-s001.zip › ijms-1576585.pdf]

Habibpourmehraban et al Supplementary Information

- Supplementary Figure S1
- Supplementary Table S1

Habibpourmehraban et al Supplementary Figure S1

IAC1131_ S2 vs C0
Proteins increased in abundance

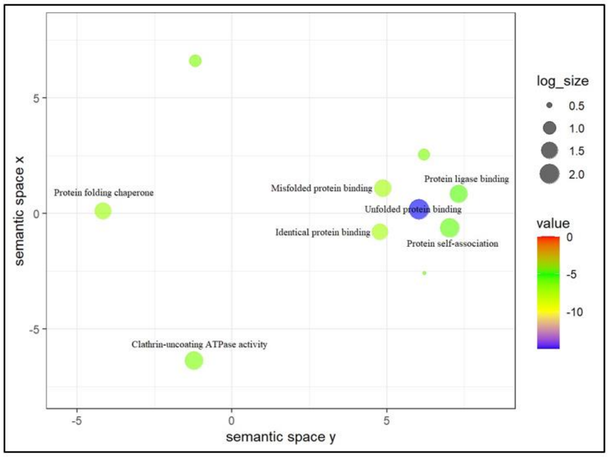

IAC1131_ S4 vs C0
Proteins increased in abundance

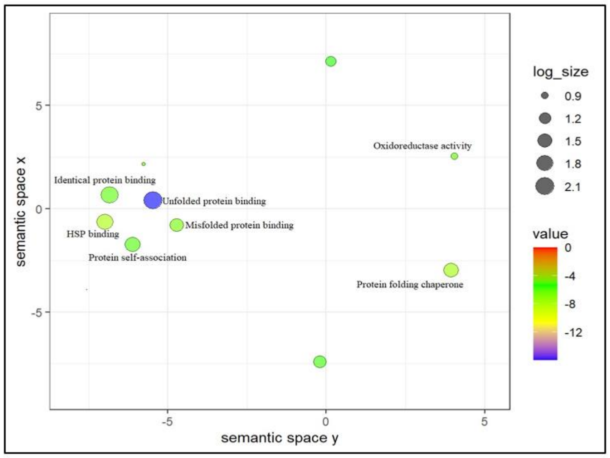

NIP_ S2 vs C0
Proteins increased in abundance

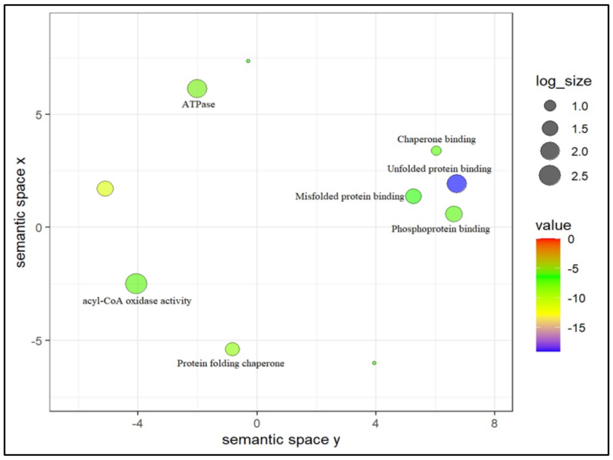

NIP_ S4 vs C0
Proteins increased in abundance

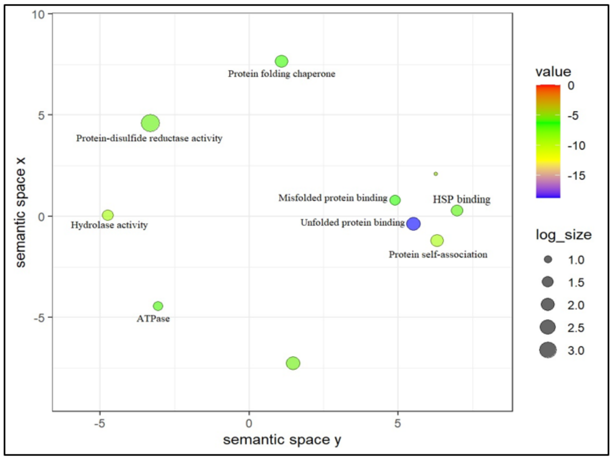

IAC1131_ S4 vs C0
Proteins decreased in abundance

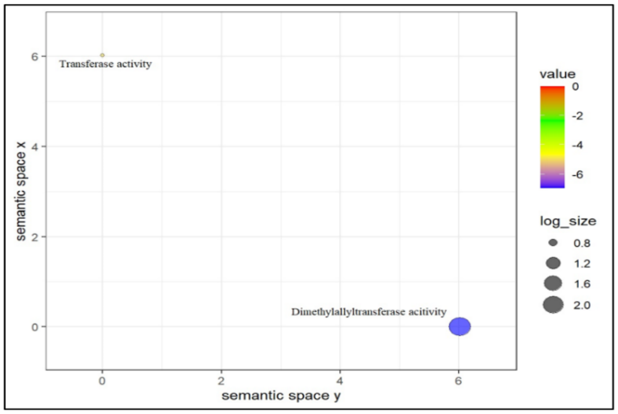

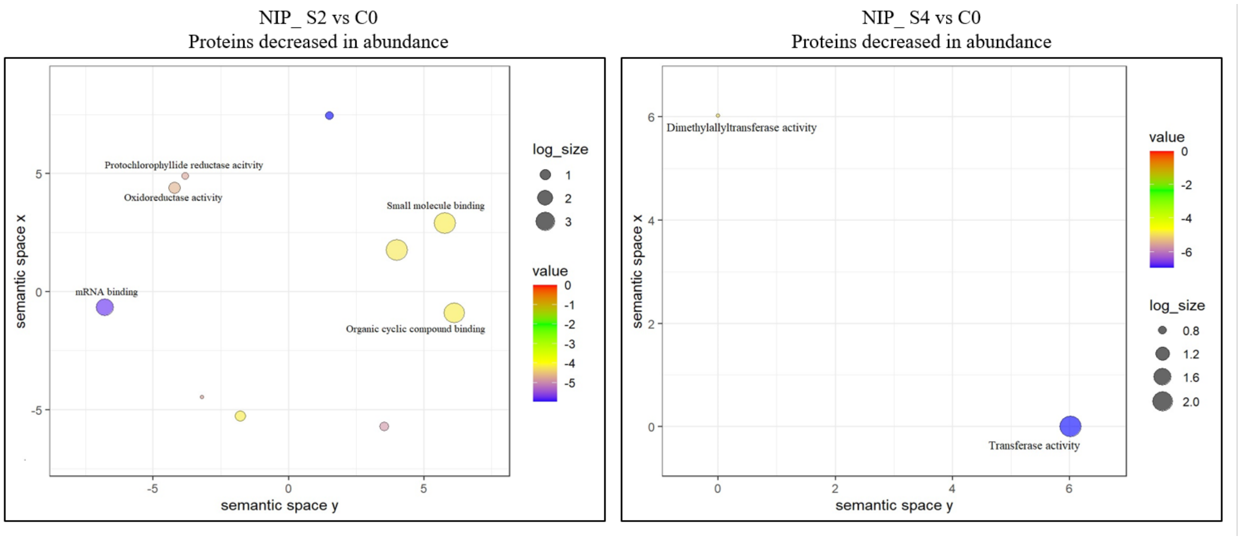

Figure S1. Molecular functions enriched (*p*-value < 0.05) in proteins increased or decreased in abundance (DEPs) in IAC1131 and Nipponbare after 2 day and 4 day multiple abiotic stress treatment. Each bubble indicates a significantly enriched term in a two-dimensional space derived by applying multidimensional scaling to a matrix of the GO terms' semantic similarities [1]. Bubble size is proportional to the frequency of the GO term in the *Oryza sativa* Uniprot database (bubbles of more general GO terms are larger), whereas color indicates the log10 *p*-value, with blue and yellow representing higher and lower *p*-value, respectively. The top 10 statistically most significant GO terms are plotted, of which only the key functions are labelled.

1. Supek, F.; Bošnjak, M.; Škunca, N.; Šmuc, T., Revigo summarizes and visualizes long lists of gene ontology terms. *PLOS ONE* **2011,** 6, (7), e21800.

Habibpourmehraban et al Supplementary Table S1

Details of all 987 Differentially Expressed Proteins

Genotypes	Treatments	Increased in Abundance	Decreased in Abundance
NIP	Control 0 day vs Stress 2 days	332	117
	Control 0 day vs. Stress 4 days	193	47
IAC	Control 0 day vs Stress 2 days	71	7
	Control 0 day vs. Stress 4 days	193	27

Total 987

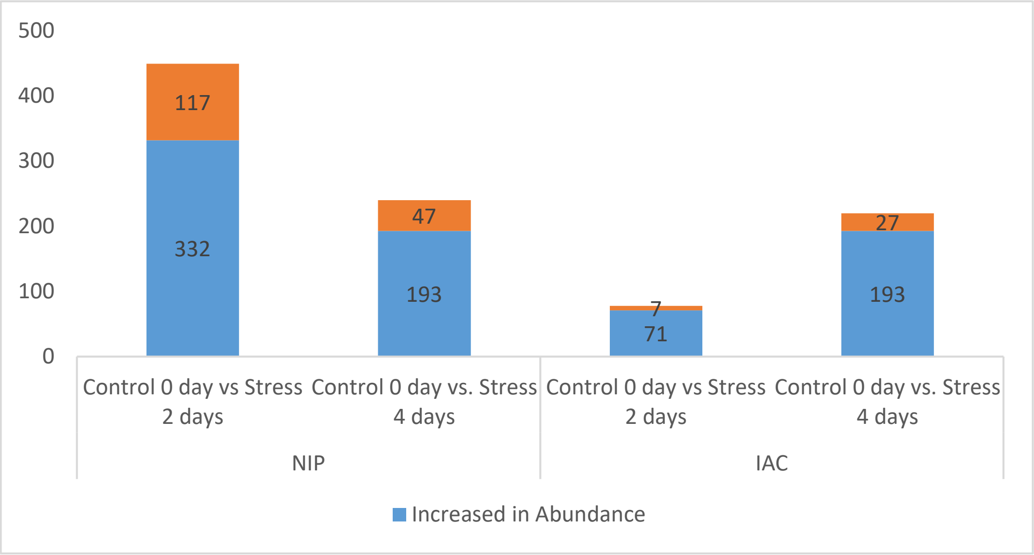

IAC_ S2 vs C0_ Proteins increased in abundance

Accession	Description	FC
P0C5A4	Late embryogenesis abundant protein 19 OS=Oryza sativa subsp. japonica GN=LEA19 PE=2 SV=1	6.38
Q94JF2	Late embryogenesis abundant protein 14 OS=Oryza sativa subsp. japonica GN=LEA14 PE=2 SV=1	5.71
Q8H4P7	Os07g0147500 protein OS=Oryza sativa subsp. japonica GN=OJ1470_H06.117 PE=2 SV=1	4.48
A0A0P0XVP1	Alpha-galactosidase OS=Oryza sativa subsp. japonica GN=Os10g0492900 PE=3 SV=1	3.39
Q84TB6	Actin-depolymerizing factor 3 OS=Oryza sativa subsp. japonica GN=ADF3 PE=1 SV=1	3.38
A0A0P0WA35	Os04g0415800 protein OS=Oryza sativa subsp. japonica GN=Os04g0415800 PE=4 SV=1	3.37
Q6ESR4	Dehydrin DHN1 OS=Oryza sativa subsp. japonica GN=DHN1 PE=2 SV=1	3.24
A0A0P0W604	Os04g0107900 protein (Fragment) OS=Oryza sativa subsp. japonica GN=Os04g0107900 PE=4 SV=1	3.23
Q5VRY1	18.0 kDa class II heat shock protein OS=Oryza sativa subsp. japonica GN=HSP18.0 PE=2 SV=1	3.23
A0A0P0WEK5	Os04g0608500 protein (Fragment) OS=Oryza sativa subsp. japonica GN=Os04g0608500 PE=4 SV=1	3.03
Q6ATC1	Os05g0160300 protein OS=Oryza sativa subsp. japonica GN=Os05g0160300 PE=2 SV=1	2.94
Q84Q72	18.1 kDa class I heat shock protein OS=Oryza sativa subsp. japonica GN=HSP18.1 PE=2 SV=1	2.82
Q84Q77	17.9 kDa class I heat shock protein OS=Oryza sativa subsp. japonica GN=HSP17.9A PE=1 SV=1	2.78
Q851F9	Probable zinc metalloprotease EGY3, chloroplastic OS=Oryza sativa subsp. japonica GN=EGY3 PE=2 SV=1	2.64
Q0DHF7	Os05g0468800 protein OS=Oryza sativa subsp. japonica GN=Os05g0468800 PE=4 SV=1	2.61
Q6F2Y7	Chaperone protein ClpB1 OS=Oryza sativa subsp. japonica GN=CLPB1 PE=2 SV=1	2.60
Q7X8R5	Thioredoxin M2, chloroplastic OS=Oryza sativa subsp. japonica GN=Os04g0530600 PE=2 SV=2	2.50
Q337E2	Expressed protein OS=Oryza sativa subsp. japonica GN=Os10g0505900 PE=4 SV=1	2.46
Q10MK4	Mitochondrial import inner membrane translocase subunit Tim17/Tim22/Tim23 family protein, putative, expressed OS=Oryza sativa subsp. japonica GN=Os03g0305600 PE=2 SV=1	2.45
Q9XFE4	Peptidylprolyl isomerase OS=Oryza sativa subsp. japonica GN=Os04g0352400 PE=2 SV=2	2.39
Q653Y0	Os06g0681200 protein OS=Oryza sativa subsp. japonica GN=Os06g0681200 PE=2 SV=1	2.37
Q0D3V0	Os07g0664400 protein OS=Oryza sativa subsp. japonica GN=Os07g0664400 PE=4 SV=1	2.34

Q84J50	17.7 kDa class I heat shock protein OS=Oryza sativa subsp. japonica GN=HSP17.7 PE=2 SV=1	2.34
Q6Z4I3	Thioredoxin H2-1 OS=Oryza sativa subsp. japonica GN=Os07g0190800 PE=2 SV=1	2.29
Q655T1	Phosphoglycerate kinase OS=Oryza sativa subsp. japonica GN=Os06g0668200 PE=2 SV=1	2.18
Q7XJY1	OSJNBb0088C09.10 protein OS=Oryza sativa subsp. japonica GN=Os04g0423400 PE=4 SV=1	2.15
Q943K7	70 kDa heat shock protein OS=Oryza sativa subsp. japonica GN=Os01g0840100 PE=2 SV=1	2.13
Q0J8R9	Os04g0690800 protein OS=Oryza sativa subsp. japonica GN=Os04g0690800 PE=4 SV=1	2.12
Q7XCS3	Cys/Met metabolism PLP-dependent enzyme family protein, expressed OS=Oryza sativa subsp. japonica GN=Os10g0517500 PE=2 SV=1	2.12
Q10N98	33 kDa secretory protein, putative, expressed OS=Oryza sativa subsp. japonica GN=Os03g0277600 PE=2 SV=1	2.12
Q6Z7V2	24.1 kDa heat shock protein, mitochondrial OS=Oryza sativa subsp. japonica GN=HSP24.1 PE=2 SV=1	2.10
Q851K1	Germin-like protein 3-6 OS=Oryza sativa subsp. japonica GN=Os03g0694000 PE=2 SV=1	2.08
Q8LNZ3	UDP-glucose 4-epimerase 1 OS=Oryza sativa subsp. japonica GN=UGE-1 PE=2 SV=1	2.01
Q8LHS0	Neurofilament triplet M protein-like protein OS=Oryza sativa subsp. japonica GN=P0039H02.109 PE=2 SV=1	1.98
A0A0P0WFD3	DNA-directed RNA polymerase subunit beta OS=Oryza sativa subsp. japonica GN=Os04g0641000 PE=3 SV=1	1.98
Q6YVU4	Os07g0539300 protein OS=Oryza sativa subsp. japonica GN=P0696F12.36-1 PE=2 SV=1	1.95
Q10NA1	Heat shock cognate 70 kDa protein, putative, expressed OS=Oryza sativa subsp. japonica GN=Os03g0277300 PE=3 SV=1	1.91
A0A0N7KP29	Os07g0683600 protein OS=Oryza sativa subsp. japonica GN=Os07g0683600 PE=4 SV=1	1.89
Q652V8	16.0 kDa heat shock protein, peroxisomal OS=Oryza sativa subsp. japonica GN=HSP16.0 PE=2 SV=1	1.86
Q7FAS1	Peroxisomal (S)-2-hydroxy-acid oxidase GLO3 OS=Oryza sativa subsp. japonica GN=GLO3 PE=2 SV=1	1.85
Q6K7E9	18.6 kDa class III heat shock protein OS=Oryza sativa subsp. japonica GN=HSP18.6 PE=2 SV=1	1.85
Q60DX1	Thiamine pyrophosphokinase 3 OS=Oryza sativa subsp. japonica GN=TPK3 PE=2 SV=1	1.84
Q0JE83	Os04g0311400 protein (Fragment) OS=Oryza sativa subsp. japonica GN=Os04g0311400 PE=3 SV=1	1.82
Q75IC7	Secretory carrier-associated membrane protein 4 OS=Oryza sativa subsp. japonica GN=SCAMP4 PE=2 SV=1	1.78
A0A0P0V4A6	Os01g0571166 protein OS=Oryza sativa subsp. japonica GN=Os01g0571166 PE=4 SV=1	1.77
Q6L509	Os05g0460000 protein OS=Oryza sativa subsp. japonica GN=Os05g0460000 PE=2 SV=1	1.76

Q7G649	Expressed protein OS=Oryza sativa subsp. japonica GN=LOC_Os10g18340 PE=2 SV=1	1.76
Q6L4Z4	Os05g0462400 protein OS=Oryza sativa subsp. japonica GN=Os05g0462400 PE=4 SV=1	1.75
A0A0P0XGD0	Os08g0425800 protein OS=Oryza sativa subsp. japonica GN=Os08g0425800 PE=4 SV=1	1.71
Q6Z312	bZIP transcription factor 23 OS=Oryza sativa subsp. japonica GN=BZIP23 PE=2 SV=1	1.69
Q7FAX1	Peroxygenase OS=Oryza sativa subsp. japonica GN=PXG PE=2 SV=1	1.66
A0A0P0VAH1	Os01g0849000 protein (Fragment) OS=Oryza sativa subsp. japonica GN=Os01g0849000 PE=4 SV=1	1.64
Q94E65	Os01g0172800 protein OS=Oryza sativa subsp. japonica GN=Os01g0172800 PE=2 SV=1	1.63
A0A0P0VFD3	Os02g0177600 protein (Fragment) OS=Oryza sativa subsp. japonica GN=Os02g0177600 PE=4 SV=1	1.62
Q7XUW5	23.2 kDa heat shock protein OS=Oryza sativa subsp. japonica GN=HSP23.2 PE=2 SV=2	1.61
Q67WJ2	ATP-dependent zinc metalloprotease FTSH 6, chloroplastic OS=Oryza sativa subsp. japonica GN=FTSH6 PE=3 SV=1	1.59
Q6Z6A7	Annexin OS=Oryza sativa subsp. japonica GN=Os02g0753800 PE=3 SV=1	1.58
Q6ATF8	Os05g0272900 protein OS=Oryza sativa subsp. japonica GN=Os05g0272900 PE=2 SV=1	1.56
Q10PW8	DnaK protein, expressed OS=Oryza sativa subsp. japonica GN=Os03g0218500 PE=2 SV=1	1.56
Q10LP5	Sucrose synthase 4 OS=Oryza sativa subsp. japonica GN=SUS4 PE=2 SV=1	1.55
Q5N770	Os01g0813900 protein OS=Oryza sativa subsp. japonica GN=Os01g0813900 PE=4 SV=1	1.54
Q40711	Dehydrin family protein, expressed OS=Oryza sativa subsp. japonica GN=Os03g0655400 PE=2 SV=1	1.54
Q6YVX5	Chloride channel protein OS=Oryza sativa subsp. japonica GN=Os02g0558100 PE=3 SV=1	1.54
Q2QY04	PP2A regulatory subunit TAP46 OS=Oryza sativa subsp. japonica GN=TAP46 PE=2 SV=1	1.54
Q5ZAV7	Os01g0783500 protein OS=Oryza sativa subsp. japonica GN=Os01g0783500 PE=2 SV=1	1.54
Q2QNV2	Fiber protein Fb19, putative, expressed OS=Oryza sativa subsp. japonica GN=LOC_Os12g36640 PE=2 SV=1	1.53
Q65XA0	Probable glutathione S-transferase DHAR1, cytosolic OS=Oryza sativa subsp. japonica GN=DHAR1 PE=1 SV=1	1.53
Q6YSB2	Glutamate decarboxylase OS=Oryza sativa subsp. japonica GN=P0104B02.16-2 PE=2 SV=1	1.53
Q69JX7	Drought-induced S-like ribonuclease OS=Oryza sativa subsp. japonica GN=Os09g0537700 PE=2 SV=1	1.53
Q5QMT0	Beta-glucosidase 1 OS=Oryza sativa subsp. japonica GN=BGLU1 PE=2 SV=1	1.52

Q7XKI7	Mitochondrial intermembrane space import and assembly protein 40 homolog OS=Oryza sativa subsp. japonica GN=MIA40 PE=2 SV=2	1.52

IAC_ S2 vs C0_ Proteins decreased in abundance

Accession	Description	FC	
Q0DKB2	Glycosyltransferase OS=Oryza sativa subsp. japonica GN=Os05g0177500 PE=2 SV=2	0.38	-2.64
Q6Z401	Sugar transport protein MST6 OS=Oryza sativa subsp. japonica GN=MST6 PE=1 SV=1	0.50	-2.00
Q5ZC82	Cytokinin riboside 5'-monophosphate phosphoribohydrolase LOG OS=Oryza sativa subsp. japonica GN=LOG PE=1 SV=1	0.60	-1.67
Q67VQ4	BolA-like OS=Oryza sativa subsp. japonica GN=Os06g0484600 PE=2 SV=1	0.60	-1.66
Q10M50	Magnesium-chelatase subunit ChlH, chloroplastic OS=Oryza sativa subsp. japonica GN=CHLH PE=1 SV=1	0.63	-1.58
Q6ZJ18	Os08g0556900 protein OS=Oryza sativa subsp. japonica GN=Os08g0556900 PE=2 SV=1	0.65	-1.55
Q0D5I5	Os07g0558300 protein OS=Oryza sativa subsp. japonica GN=Os07g0558300 PE=2 SV=1	0.66	-1.52

IAC_ S4 vs C0_ Proteins increased in abundance

Accession	Description	FC
P0C5A4	Late embryogenesis abundant protein 19 OS=Oryza sativa subsp. japonica GN=LEA19 PE=2 SV=1	7.79
Q8H4P7	Os07g0147500 protein OS=Oryza sativa subsp. japonica GN=OJ1470_H06.117 PE=2 SV=1	7.03
Q5VRY1	18.0 kDa class II heat shock protein OS=Oryza sativa subsp. japonica GN=HSP18.0 PE=2 SV=1	5.36
Q84TB6	Actin-depolymerizing factor 3 OS=Oryza sativa subsp. japonica GN=ADF3 PE=1 SV=1	4.60
Q0J8R9	Os04g0690800 protein OS=Oryza sativa subsp. japonica GN=Os04g0690800 PE=4 SV=1	4.23
Q94JF2	Late embryogenesis abundant protein 14 OS=Oryza sativa subsp. japonica GN=LEA14 PE=2 SV=1	3.98
Q84Q77	17.9 kDa class I heat shock protein OS=Oryza sativa subsp. japonica GN=HSP17.9A PE=1 SV=1	3.96
Q7X8R5	Thioredoxin M2, chloroplastic OS=Oryza sativa subsp. japonica GN=Os04g0530600 PE=2 SV=2	3.91
Q10MK4	Mitochondrial import inner membrane translocase subunit Tim17/Tim22/Tim23 family protein, putative, expressed OS=Oryza sativa subsp. japonica GN=Os03g0305600 PE=2 SV=1	3.80
Q10NA1	Heat shock cognate 70 kDa protein, putative, expressed OS=Oryza sativa subsp. japonica GN=Os03g0277300 PE=3 SV=1	3.54
O04138	Chitinase 4 OS=Oryza sativa subsp. japonica GN=Cht4 PE=2 SV=2	3.45
A0A0P0XVP1	Alpha-galactosidase OS=Oryza sativa subsp. japonica GN=Os10g0492900 PE=3 SV=1	3.30
Q84Q72	18.1 kDa class I heat shock protein OS=Oryza sativa subsp. japonica GN=HSP18.1 PE=2 SV=1	3.30
Q655T1	Phosphoglycerate kinase OS=Oryza sativa subsp. japonica GN=Os06g0668200 PE=2 SV=1	3.26
A0A0P0W604	Os04g0107900 protein (Fragment) OS=Oryza sativa subsp. japonica GN=Os04g0107900 PE=4 SV=1	3.26
A0A0P0WA35	Os04g0415800 protein OS=Oryza sativa subsp. japonica GN=Os04g0415800 PE=4 SV=1	3.25
Q6Z4I3	Thioredoxin H2-1 OS=Oryza sativa subsp. japonica GN=Os07g0190800 PE=2 SV=1	3.20
Q653Y0	Os06g0681200 protein OS=Oryza sativa subsp. japonica GN=Os06g0681200 PE=2 SV=1	3.20
Q6K7E9	18.6 kDa class III heat shock protein OS=Oryza sativa subsp. japonica GN=HSP18.6 PE=2 SV=1	3.20
Q851K1	Germin-like protein 3-6 OS=Oryza sativa subsp. japonica GN=Os03g0694000 PE=2 SV=1	3.13
Q851F9	Probable zinc metalloprotease EGY3, chloroplastic OS=Oryza sativa subsp. japonica GN=EGY3 PE=2 SV=1	3.07

Q652V8	16.0 kDa heat shock protein, peroxisomal OS=Oryza sativa subsp. japonica GN=HSP16.0 PE=2 SV=1	3.06
Q0DHF7	Os05g0468800 protein OS=Oryza sativa subsp. japonica GN=Os05g0468800 PE=4 SV=1	3.04
Q337E2	Expressed protein OS=Oryza sativa subsp. japonica GN=Os10g0505900 PE=4 SV=1	3.01
Q9XFE4	Peptidylprolyl isomerase OS=Oryza sativa subsp. japonica GN=Os04g0352400 PE=2 SV=2	2.98
Q2QLS7	Os12g0630200 protein OS=Oryza sativa subsp. japonica GN=Os12g0630200 PE=4 SV=1	2.94
Q10QC5	Os03g0201600 protein OS=Oryza sativa subsp. japonica GN=Os03g0201600 PE=2 SV=1	2.83
Q7XJY1	OSJNBb0088C09.10 protein OS=Oryza sativa subsp. japonica GN=Os04g0423400 PE=4 SV=1	2.74
Q9FWU4	Os10g0491000 protein OS=Oryza sativa subsp. japonica GN=LOC_Os10g34930 PE=2 SV=1	2.71
Q6ESR4	Dehydrin DHN1 OS=Oryza sativa subsp. japonica GN=DHN1 PE=2 SV=1	2.70
Q8LHS0	Neurofilament triplet M protein-like protein OS=Oryza sativa subsp. japonica GN=P0039H02.109 PE=2 SV=1	2.68
Q5VP66	Os01g0644000 protein OS=Oryza sativa subsp. japonica GN=Os01g0644000 PE=2 SV=1	2.64
Q94CS9	Probable aquaporin TIP1-2 OS=Oryza sativa subsp. japonica GN=TIP1-2 PE=2 SV=1	2.64
Q94CR1	Beta 1,3-glucanase OS=Oryza sativa subsp. japonica GN=P0660F12.19 PE=2 SV=1	2.63
Q84J50	17.7 kDa class I heat shock protein OS=Oryza sativa subsp. japonica GN=HSP17.7 PE=2 SV=1	2.55
A0A0P0WAD7	Os04g0423600 protein OS=Oryza sativa subsp. japonica GN=Os04g0423600 PE=4 SV=1	2.53
Q6ZCR3	Germin-like protein 8-12 OS=Oryza sativa subsp. japonica GN=Os08g0231400 PE=2 SV=1	2.50
Q943K7	70 kDa heat shock protein OS=Oryza sativa subsp. japonica GN=Os01g0840100 PE=2 SV=1	2.47
Q9FYR9	Class III chitinase OS=Oryza sativa subsp. japonica GN=chib1 PE=2 SV=1	2.44
Q10N98	33 kDa secretory protein, putative, expressed OS=Oryza sativa subsp. japonica GN=Os03g0277600 PE=2 SV=1	2.42
A0A0P0XGD0	Os08g0425800 protein OS=Oryza sativa subsp. japonica GN=Os08g0425800 PE=4 SV=1	2.42
Q2R5M2	Carboxypeptidase OS=Oryza sativa subsp. japonica GN=LOC_Os11g24510 PE=3 SV=1	2.40
Q6YVU4	Os07g0539300 protein OS=Oryza sativa subsp. japonica GN=P0696F12.36-1 PE=2 SV=1	2.31
A0A0P0W1B6	Os03g0661600 protein (Fragment) OS=Oryza sativa subsp. japonica GN=Os03g0661600 PE=4 SV=1	2.31
A0A0N7KP29	Os07g0683600 protein OS=Oryza sativa subsp. japonica GN=Os07g0683600 PE=4 SV=1	2.28

Q7FAX1	Peroxygenase OS=Oryza sativa subsp. japonica GN=PXG PE=2 SV=1	2.26
P27777	16.9 kDa class I heat shock protein 1 OS=Oryza sativa subsp. japonica GN=HSP16.9A PE=1 SV=1	2.26
Q10MP7	Os03g0300400 protein OS=Oryza sativa subsp. japonica GN=Os03g0300400 PE=2 SV=1	2.22
Q7G649	Expressed protein OS=Oryza sativa subsp. japonica GN=LOC_Os10g18340 PE=2 SV=1	2.18
Q8S1C7	Cytochrome P450 (CYP72C)-like OS=Oryza sativa subsp. japonica GN=CYP72A32 PE=2 SV=1	2.16
Q0JNL7	Calmodulin-3 OS=Oryza sativa subsp. japonica GN=CAM3 PE=2 SV=1	2.16
Q94E74	Os01g0511100 protein OS=Oryza sativa subsp. japonica GN=Os01g0511100 PE=2 SV=1	2.16
Q6Z7V2	24.1 kDa heat shock protein, mitochondrial OS=Oryza sativa subsp. japonica GN=HSP24.1 PE=2 SV=1	2.15
Q42993	Chitinase 1 OS=Oryza sativa subsp. japonica GN=Cht1 PE=2 SV=1	2.14
Q6L509	Os05g0460000 protein OS=Oryza sativa subsp. japonica GN=Os05g0460000 PE=2 SV=1	2.13
Q9XHY5	Os01g0246400 protein OS=Oryza sativa subsp. japonica GN=OSJNBa0049B20.7 PE=2 SV=1	2.12
B9FYM4	Os08g0101400 protein OS=Oryza sativa subsp. japonica GN=Os08g0101400 PE=4 SV=1	2.12
Q6F2Y7	Chaperone protein ClpB1 OS=Oryza sativa subsp. japonica GN=CLPB1 PE=2 SV=1	2.08
Q5N7Y1	Os01g0910900 protein OS=Oryza sativa subsp. japonica GN=Os01g0910900 PE=2 SV=1	2.08
Q0D840	Thioredoxin H1 OS=Oryza sativa subsp. japonica GN=TRXH PE=1 SV=1	2.05
Q0IZF1	Os09g0572700 protein OS=Oryza sativa subsp. japonica GN=Os09g0572700 PE=2 SV=1	2.04
Q8RU06	Cellulase containing protein, expressed OS=Oryza sativa subsp. japonica GN=OJ1208D02.5 PE=2 SV=1	2.04
B9FCZ7	Os04g0663700 protein OS=Oryza sativa subsp. japonica GN=Os04g0663700 PE=4 SV=1	2.03
Q2QQS1	KE2 family protein, expressed OS=Oryza sativa subsp. japonica GN=Os12g0485800 PE=2 SV=1	2.02
Q0IQK9	Non-specific lipid-transfer protein 1 OS=Oryza sativa subsp. japonica GN=LTP PE=1 SV=1	2.00
Q7XJ39	Non-specific lipid-transfer protein 2A OS=Oryza sativa subsp. japonica GN=LTP2-A PE=3 SV=2	2.00
Q6AUF2	Os05g0565400 protein OS=Oryza sativa subsp. japonica GN=Os05g0565400 PE=2 SV=1	1.99
A0A0P0V486	Os01g0571100 protein (Fragment) OS=Oryza sativa subsp. japonica GN=Os01g0571100 PE=4 SV=1	1.98
A0A0P0W3Z6	Os03g0750100 protein OS=Oryza sativa subsp. japonica GN=Os03g0750100 PE=4 SV=1	1.94
Q6ETD9	Calmodulin-binding protein-like OS=Oryza sativa subsp. japonica GN=Os02g0105500 PE=2 SV=1	1.93

Q6Z493	Protein DETOXIFICATION OS=Oryza sativa subsp. japonica GN=Os07g0502200 PE=3 SV=1	1.92
Q7XXQ8	Os06g0232000 protein OS=Oryza sativa subsp. japonica GN=Os06g0232000 PE=2 SV=1	1.91
Q6YW53	CASP-like protein 2D1 OS=Oryza sativa subsp. japonica GN=Os02g0219900 PE=2 SV=1	1.89
Q7XEL9	Chitinase 1, putative, expressed OS=Oryza sativa subsp. japonica GN=Os10g0416800 PE=2 SV=1	1.88
Q656J2	Reticulon-like protein OS=Oryza sativa subsp. japonica GN=Os06g0503400 PE=2 SV=1	1.88
Q10PW8	DnaK protein, expressed OS=Oryza sativa subsp. japonica GN=Os03g0218500 PE=2 SV=1	1.88
Q6Z563	Os08g0412800 protein OS=Oryza sativa subsp. japonica GN=Os08g0412800 PE=2 SV=1	1.87
Q2QNV2	Fiber protein Fb19, putative, expressed OS=Oryza sativa subsp. japonica GN=LOC_Os12g36640 PE=2 SV=1	1.86
Q0JIL1	Probable nucleoredoxin 2 OS=Oryza sativa subsp. japonica GN=Os01g0794400 PE=2 SV=1	1.85
Q67WJ2	ATP-dependent zinc metalloprotease FTSH 6, chloroplastic OS=Oryza sativa subsp. japonica GN=FTSH6 PE=3 SV=1	1.85
Q0DI48	Thioredoxin-like fold domain-containing protein MRL7L homolog, chloroplastic OS=Oryza sativa subsp. japonica GN=MRL7L PE=2 SV=1	1.84
A0A0P0VVF7	Os03g0248200 protein (Fragment) OS=Oryza sativa subsp. japonica GN=Os03g0248200 PE=3 SV=1	1.84
Q10LP5	Sucrose synthase 4 OS=Oryza sativa subsp. japonica GN=SUS4 PE=2 SV=1	1.83
A0A0P0V4A6	Os01g0571166 protein OS=Oryza sativa subsp. japonica GN=Os01g0571166 PE=4 SV=1	1.83
Q7XXD3	OSJNBa0039G19.10 protein OS=Oryza sativa subsp. japonica GN=Os04g0175600 PE=2 SV=2	1.83
Q5VRJ8	Peroxisomal membrane protein 11-5 OS=Oryza sativa subsp. japonica GN=PEX11-5 PE=2 SV=1	1.82
A0A0P0WLH4	Os05g0364600 protein (Fragment) OS=Oryza sativa subsp. japonica GN=Os05g0364600 PE=4 SV=1	1.82
Q5ZBR8	Os01g0795000 protein OS=Oryza sativa subsp. japonica GN=Os01g0795000 PE=4 SV=1	1.80
Q53K52	Protein PEP-RELATED DEVELOPMENT ARRESTED 1 homolog, chloroplastic OS=Oryza sativa subsp. japonica GN=Os11g0425300 PE=2 SV=1	1.80
Q6F391	Expressed protein OS=Oryza sativa subsp. japonica GN=OSJNBb0021G19.8 PE=2 SV=1	1.79
Q6ZFJ9	60 kDa chaperonin beta subunit OS=Oryza sativa subsp. japonica GN=Os02g0102900 PE=2 SV=1	1.78
Q5QMT0	Beta-glucosidase 1 OS=Oryza sativa subsp. japonica GN=BGLU1 PE=2 SV=1	1.77
Q10PB0	Expressed protein OS=Oryza sativa subsp. japonica GN=LOC_Os03g13750 PE=2 SV=1	1.77
Q7XUW5	23.2 kDa heat shock protein OS=Oryza sativa subsp. japonica GN=HSP23.2 PE=2 SV=2	1.76

Q338P6	Os10g0389200 protein OS=Oryza sativa subsp. japonica GN=Os10g0389200 PE=2 SV=1	1.75
Q60ER3	Os05g0393400 protein OS=Oryza sativa subsp. japonica GN=Os05g0393400 PE=2 SV=1	1.74
A0A0P0W7K1	Glycerol-3-phosphate dehydrogenase OS=Oryza sativa subsp. japonica GN=Os04g0225001 PE=3 SV=1	1.73
Q6Z7K5	Metal tolerance protein 3 OS=Oryza sativa subsp. japonica GN=MTP3 PE=2 SV=1	1.73
Q2QNQ6	Expressed protein OS=Oryza sativa subsp. japonica GN=Os12g0557400 PE=2 SV=1	1.72
Q75IC7	Secretory carrier-associated membrane protein 4 OS=Oryza sativa subsp. japonica GN=SCAMP4 PE=2 SV=1	1.72
Q656E2	Endoribonuclease E-like protein OS=Oryza sativa subsp. japonica GN=P0552C05.41-1 PE=4 SV=1	1.71
Q8LNZ3	UDP-glucose 4-epimerase 1 OS=Oryza sativa subsp. japonica GN=UGE-1 PE=2 SV=1	1.71
Q10SR3	70 kDa heat shock protein OS=Oryza sativa subsp. japonica GN=Os03g0113700 PE=2 SV=1	1.71
Q852G4	EF hand family protein, expressed OS=Oryza sativa subsp. japonica GN=Os03g0411300 PE=2 SV=1	1.70
Q5QNJ0	Os01g0214600 protein OS=Oryza sativa subsp. japonica GN=Os01g0214600 PE=4 SV=1	1.70
Q53JF7	ABA/WDS induced protein, expressed OS=Oryza sativa subsp. japonica GN=Os11g0167800 PE=2 SV=1	1.70
Q7FAS1	Peroxisomal (S)-2-hydroxy-acid oxidase GLO3 OS=Oryza sativa subsp. japonica GN=GLO3 PE=2 SV=1	1.70
Q67VZ1	Annexin OS=Oryza sativa subsp. japonica GN=Os06g0221200 PE=2 SV=1	1.70
Q7XIV4	Alpha-galactosidase OS=Oryza sativa subsp. japonica GN=Os07g0679300 PE=2 SV=1	1.70
Q6YSB2	Glutamate decarboxylase OS=Oryza sativa subsp. japonica GN=P0104B02.16-2 PE=2 SV=1	1.69
Q6ATF8	Os05g0272900 protein OS=Oryza sativa subsp. japonica GN=Os05g0272900 PE=2 SV=1	1.69
Q8H367	Os07g0413800 protein OS=Oryza sativa subsp. japonica GN=Os07g0413800 PE=2 SV=1	1.69
Q0DCI1	Pyrophosphate--fructose 6-phosphate 1-phosphotransferase subunit alpha OS=Oryza sativa subsp. japonica GN=Os06g0326400 PE=2 SV=1	1.68
Q75L18	Os05g0112800 protein OS=Oryza sativa subsp. japonica GN=Os05g0112800 PE=2 SV=1	1.68
Q7XW88	OSJNBb0043H09.9 protein OS=Oryza sativa subsp. japonica GN=Os04g0244400 PE=2 SV=2	1.68
Q75LD9	Os03g0843300 protein OS=Oryza sativa subsp. japonica GN=OSJNBa0032G11.5 PE=2 SV=1	1.68
Q5ZDL5	Os01g0147700 protein OS=Oryza sativa subsp. japonica GN=Os01g0147700 PE=4 SV=1	1.68
Q7XVC0	OSJNBa0072D21.10 protein OS=Oryza sativa subsp. japonica GN=Os04g0382300 PE=2 SV=2	1.67

Q10PD0	Purple acid phosphatase OS=Oryza sativa subsp. japonica GN=LOC_Os03g13540 PE=2 SV=1	1.66
Q6H611	Succinate dehydrogenase subunit 7, mitochondrial OS=Oryza sativa subsp. japonica GN=SDH7 PE=1 SV=1	1.66
Q5N770	Os01g0813900 protein OS=Oryza sativa subsp. japonica GN=Os01g0813900 PE=4 SV=1	1.66
A0A0N7KFB0	Os02g0485000 protein (Fragment) OS=Oryza sativa subsp. japonica GN=Os02g0485000 PE=4 SV=1	1.65
Q5ZAV7	Os01g0783500 protein OS=Oryza sativa subsp. japonica GN=Os01g0783500 PE=2 SV=1	1.65
Q6YVX5	Chloride channel protein OS=Oryza sativa subsp. japonica GN=Os02g0558100 PE=3 SV=1	1.65
Q5JJV9	Os01g0965400 protein OS=Oryza sativa subsp. japonica GN=Os01g0965400 PE=2 SV=1	1.64
Q5QL78	Os01g0524700 protein OS=Oryza sativa subsp. japonica GN=Os01g0524700 PE=2 SV=1	1.64
Q2QN15	Early-responsive to dehydration protein, putative, expressed OS=Oryza sativa subsp. japonica GN=Os12g0582800 PE=4 SV=1	1.64
Q6AUV3	Os03g0430000 protein OS=Oryza sativa subsp. japonica GN=OSJNBa0091B22.9 PE=2 SV=1	1.64
A0A0P0VQF9	Os02g0783625 protein OS=Oryza sativa subsp. japonica GN=Os02g0783625 PE=4 SV=1	1.63
Q0JD56	Os04g0430200 protein OS=Oryza sativa subsp. japonica GN=Os04g0430200 PE=2 SV=1	1.63
P0C5D6	Serine/threonine-protein kinase SAPK3 OS=Oryza sativa subsp. japonica GN=SAPK3 PE=1 SV=1	1.63
A0A0P0XAA0	Os07g0671800 protein (Fragment) OS=Oryza sativa subsp. japonica GN=Os07g0671800 PE=4 SV=1	1.63
O04226	Delta-1-pyrroline-5-carboxylate synthase 1 OS=Oryza sativa subsp. japonica GN=P5CS1 PE=2 SV=2	1.63
Q94DD0	Os01g0859200 protein OS=Oryza sativa subsp. japonica GN=Os01g0859200 PE=2 SV=1	1.63
Q7XR60	Methylthioribose kinase 2 OS=Oryza sativa subsp. japonica GN=MTK2 PE=2 SV=1	1.62
C7J056	Os03g0859600 protein (Fragment) OS=Oryza sativa subsp. japonica GN=Os03g0859600 PE=3 SV=1	1.62
Q8S2H5	Purple acid phosphatase OS=Oryza sativa subsp. japonica GN=P0003D09.8-1 PE=2 SV=1	1.62
Q6Z3Y9	Phosphoinositide phospholipase C OS=Oryza sativa subsp. japonica GN=Os07g0694000 PE=2 SV=1	1.62
Q337M4	Os10g0463800 protein OS=Oryza sativa subsp. japonica GN=Os10g0463800 PE=2 SV=1	1.61
B9F3B6	Succinate-semialdehyde dehydrogenase, mitochondrial OS=Oryza sativa subsp. japonica GN=ALDH5F1 PE=3 SV=1	1.61
Q69XR3	Alpha/beta hydrolase-fold family protein OS=Oryza sativa subsp. japonica GN=P0526E12.28-1 PE=2 SV=1	1.61
Q10PW2	Tubulin alpha chain OS=Oryza sativa subsp. japonica GN=TubA PE=2 SV=1	1.61

Q6Z312	bZIP transcription factor 23 OS=Oryza sativa subsp. japonica GN=BZIP23 PE=2 SV=1	1.61
Q5Z7I5	Os06g0548200 protein OS=Oryza sativa subsp. japonica GN=Os06g0548200 PE=3 SV=1	1.61
Q8RU95	4-coumarate--CoA ligase-like 6 OS=Oryza sativa subsp. japonica GN=4CLL6 PE=2 SV=2	1.60
Q5ZAV6	Os01g0783600 protein OS=Oryza sativa subsp. japonica GN=Os01g0783600 PE=2 SV=1	1.60
Q5VRH4	Homogentisate 1,2-dioxygenase OS=Oryza sativa subsp. japonica GN=HGO PE=2 SV=1	1.59
Q6ZHJ5	Pentatricopeptide repeat-containing protein OTP51, chloroplastic OS=Oryza sativa subsp. japonica GN=OTP51 PE=3 SV=1	1.59
Q5NA77	C2 domain-containing protein-like OS=Oryza sativa subsp. japonica GN=Os01g0242600 PE=2 SV=1	1.59
Q69LD9	Os07g0169600 protein OS=Oryza sativa subsp. japonica GN=Os07g0169600 PE=2 SV=1	1.59
Q6Z244	Os08g0480200 protein OS=Oryza sativa subsp. japonica GN=Os08g0480200 PE=2 SV=1	1.58
Q762A5	BRI1-KD interacting protein 109 OS=Oryza sativa subsp. japonica GN=bip109 PE=2 SV=1	1.58
Q8S7M7	Plant intracellular Ras-group-related LRR protein 5 OS=Oryza sativa subsp. japonica GN=IRL5 PE=2 SV=1	1.58
Q2QXG8	Os12g0157200 protein OS=Oryza sativa subsp. japonica GN=LOC_Os12g06100 PE=4 SV=1	1.58
Q0JEP5	Os04g0224900 protein (Fragment) OS=Oryza sativa subsp. japonica GN=Os04g0224900 PE=4 SV=1	1.57
Q6Z6A7	Annexin OS=Oryza sativa subsp. japonica GN=Os02g0753800 PE=3 SV=1	1.57
Q6H7M1	Fumarylacetoacetase OS=Oryza sativa subsp. japonica GN=FAH PE=2 SV=1	1.57
Q6ZBK6	Os08g0519400 protein OS=Oryza sativa subsp. japonica GN=Os08g0519400 PE=4 SV=1	1.56
Q5QLQ5	Os01g0667200 protein OS=Oryza sativa subsp. japonica GN=Os01g0667200 PE=2 SV=1	1.56
A0A0P0WMW0	Os05g0432700 protein (Fragment) OS=Oryza sativa subsp. japonica GN=Os05g0432700 PE=4 SV=1	1.56
Q65XA0	Probable glutathione S-transferase DHAR1, cytosolic OS=Oryza sativa subsp. japonica GN=DHAR1 PE=1 SV=1	1.56
B9FRA2	Os06g0127500 protein OS=Oryza sativa subsp. japonica GN=Os06g0127500 PE=4 SV=1	1.56
Q7F7I7	GTP-binding nuclear protein Ran-1 OS=Oryza sativa subsp. japonica GN=RAN1 PE=2 SV=1	1.56
Q75IM9	Isovaleryl-CoA dehydrogenase, mitochondrial OS=Oryza sativa subsp. japonica GN=Os05g0125500 PE=2 SV=2	1.56
Q69P84	Aldehyde dehydrogenase OS=Oryza sativa subsp. japonica GN=OJ1344_B01.27-1 PE=2 SV=1	1.55
A0A0P0UYD8	Os01g0155400 protein (Fragment) OS=Oryza sativa subsp. japonica GN=Os01g0155400 PE=4 SV=1	1.55

A0A0P0UYG1	Os01g0155500 protein (Fragment) OS=Oryza sativa subsp. japonica GN=Os01g0155500 PE=3 SV=1	1.55
Q5W6X4	Os05g0316200 protein OS=Oryza sativa subsp. japonica GN=Os05g0316200 PE=2 SV=1	1.55
Q2R1J1	ABC transporter family protein, expressed OS=Oryza sativa subsp. japonica GN=Os11g0603200 PE=2 SV=1	1.55
Q2QXL3	ACT domain-containing protein, putative, expressed OS=Oryza sativa subsp. japonica GN=Os12g0152700 PE=2 SV=1	1.55
Q94E65	Os01g0172800 protein OS=Oryza sativa subsp. japonica GN=Os01g0172800 PE=2 SV=1	1.55
Q5Z4C9	Os06g0306600 protein OS=Oryza sativa subsp. japonica GN=Os06g0306600 PE=2 SV=1	1.55
Q2QTY6	Lysosomal Cystine Transporter family protein, expressed OS=Oryza sativa subsp. japonica GN=Os12g0278700 PE=2 SV=2	1.55
Q10MJ3	Os03g0306900 protein OS=Oryza sativa subsp. japonica GN=Os03g0306900 PE=2 SV=1	1.54
Q6L4X5	Thioredoxin OS=Oryza sativa subsp. japonica GN=Os05g0508500 PE=3 SV=1	1.54
Q6ZCF3	Os08g0205400 protein OS=Oryza sativa subsp. japonica GN=Os08g0205400 PE=2 SV=1	1.53
B9FCW0	Os04g0652700 protein OS=Oryza sativa subsp. japonica GN=Os04g0652700 PE=4 SV=1	1.53
Q75M67	Expressed protein OS=Oryza sativa subsp. japonica GN=Os03g0381300 PE=4 SV=1	1.53
Q7XRE7	OSJNBa0006M15.20 protein OS=Oryza sativa subsp. japonica GN=OSJNBa0036B21.4 PE=2 SV=2	1.53
Q5Z9P6	Os06g0715500 protein OS=Oryza sativa subsp. japonica GN=Os06g0715500 PE=2 SV=1	1.52
Q2QME6	Homocysteine S-methyltransferase 3 OS=Oryza sativa subsp. japonica GN=Os12g0607000 PE=2 SV=2	1.52
Q948T6	Lactoylglutathione lyase OS=Oryza sativa subsp. japonica GN=GLYI-11 PE=1 SV=2	1.52
Q0JNR2	Cysteine proteinase inhibitor 12 OS=Oryza sativa subsp. japonica GN=Os01g0270100 PE=2 SV=1	1.51
Q69LD2	Os07g0170100 protein OS=Oryza sativa subsp. japonica GN=Os07g0170100 PE=2 SV=1	1.51
Q6H7E4	Thioredoxin M1, chloroplastic OS=Oryza sativa subsp. japonica GN=Os02g0639900 PE=2 SV=1	1.51
Q6L4H1	Os05g0550300 protein OS=Oryza sativa subsp. japonica GN=P0560C03.5 PE=2 SV=1	1.51
A3AWZ0	Os04g0593400 protein OS=Oryza sativa subsp. japonica GN=Os04g0593400 PE=4 SV=1	1.51
Q6H7R1	Os02g0643000 protein OS=Oryza sativa subsp. japonica GN=Os02g0643000 PE=2 SV=1	1.51
Q6K3E9	F-box family protein-like OS=Oryza sativa subsp. japonica GN=Os02g0813350 PE=2 SV=1	1.51
Q75LJ3	Electron transfer flavoprotein subunit alpha, mitochondrial OS=Oryza sativa subsp. japonica GN=ETFA PE=2 SV=1	1.50

Q9FRA7	REF/SRPP-like protein Os05g0151300/LOC_Os05g05940 OS=Oryza sativa subsp. japonica GN=Os05g0151300 PE=2 SV=2	1.50
Q6H475	Os02g0631000 protein OS=Oryza sativa subsp. japonica GN=Os02g0631000 PE=2 SV=1	1.50
A0A0P0UZ39	Os01g0190000 protein OS=Oryza sativa subsp. japonica GN=Os01g0190000 PE=4 SV=1	1.50

IAC_ S4 vs C0_ Proteins decreased in abundance

Accession	Description	FC	
Q6K209	Os02g0629800 protein OS=Oryza sativa subsp. japonica GN=Os02g0629800 PE=3 SV=1	0.46	-2.18
Q7XUY5	OSJNBb0048E02.12 protein OS=Oryza sativa subsp. japonica GN=Os04g0465600 PE=2 SV=1	0.49	-2.06
Q5NAM3	Branched-chain amino acid aminotransferase-like OS=Oryza sativa subsp. japonica GN=Os01g0238500 PE=4 SV=1	0.49	-2.05
P31924	Sucrose synthase 1 OS=Oryza sativa subsp. japonica GN=SUS1 PE=1 SV=1	0.51	-1.97
Q6K826	Os02g0781400 protein OS=Oryza sativa subsp. japonica GN=OJ1369_G08.10-1 PE=2 SV=1	0.52	-1.93
B7EYZ0	Os05g0456300 protein OS=Oryza sativa subsp. japonica GN=Os05g0456300 PE=2 SV=1	0.54	-1.85
Q0D5I5	Os07g0558300 protein OS=Oryza sativa subsp. japonica GN=Os07g0558300 PE=2 SV=1	0.57	-1.75
Q8L562	Hydrolase-like OS=Oryza sativa subsp. japonica GN=Os01g0595600 PE=2 SV=1	0.58	-1.73
Q6H6D2	Porphobilinogen deaminase, chloroplastic OS=Oryza sativa subsp. japonica GN=HEMC PE=2 SV=1	0.59	-1.71
Q6ZLK8	Os07g0134000 protein OS=Oryza sativa subsp. japonica GN=OJ1118_D07.26-1 PE=2 SV=1	0.59	-1.70
Q7XI92	Os07g0580900 protein OS=Oryza sativa subsp. japonica GN=Os07g0580900 PE=1 SV=1	0.59	-1.68
Q9LRE9	Cytosolic aldehyde dehydrogenase OS=Oryza sativa subsp. japonica GN=ALDH1a PE=2 SV=1	0.60	-1.67
Q7F9Y6	OSJNBa0086O06.22 protein OS=Oryza sativa subsp. japonica GN=Os04g0591000 PE=2 SV=1	0.61	-1.64
Q0DTF5	Os03g0251000 protein OS=Oryza sativa subsp. japonica GN=Os03g0251000 PE=4 SV=1	0.63	-1.59
Q7XS58	Cysteine synthase OS=Oryza sativa subsp. japonica GN=Os04g0165700 PE=2 SV=2	0.63	-1.59
Q6ATB2	Probable GTP diphosphokinase CRSH2, chloroplastic OS=Oryza sativa subsp. japonica GN=CRSH2 PE=2 SV=1	0.63	-1.58
Q6ET88	Os02g0668100 protein OS=Oryza sativa subsp. japonica GN=Os02g0668100 PE=1 SV=1	0.63	-1.58
Q6ZBZ8	Os08g0459300 protein OS=Oryza sativa subsp. japonica GN=Os08g0459300 PE=2 SV=1	0.64	-1.57
Q945W2	Glutathione S-transferase GSTU6, putative, expressed OS=Oryza sativa subsp. japonica GN=Os10g0529300 PE=2 SV=1	0.64	-1.55
B7FA34	Os05g0548900 protein OS=Oryza sativa subsp. japonica GN=Os05g0548900 PE=2 SV=1	0.64	-1.55
Q0JGA3	Os01g0934400 protein (Fragment) OS=Oryza sativa subsp. japonica GN=Os01g0934400 PE=4 SV=1	0.65	-1.55

Q10LR9	Uroporphyrinogen decarboxylase 2, chloroplastic OS=Oryza sativa subsp. japonica GN=Os03g0337600 PE=3 SV=1	0.65	-1.53
P12139	50S ribosomal protein L20, chloroplastic OS=Oryza sativa subsp. japonica GN=rpl20 PE=3 SV=3	0.66	-1.53
Q6ZGU9	Expansin-A5 OS=Oryza sativa subsp. japonica GN=EXPA5 PE=2 SV=1	0.66	-1.52
Q6Z6Y1	Os02g0130100 protein OS=Oryza sativa subsp. japonica GN=Os02g0130100 PE=2 SV=1	0.66	-1.51
A0A0P0Y8(	Os12g0263000 protein (Fragment) OS=Oryza sativa subsp. japonica GN=Os12g0263000 PE=4 SV=1	0.66	-1.51
Q6ASX7	Glycine-rich RNA binding protein OS=Oryza sativa subsp. japonica GN=Os03g0670700 PE=2 SV=1	0.66	-1.51

NIP_ S2 vs C0_ Proteins increased in abundance

Accession	Description	FC
Q6Z6M4	Isocitrate lyase OS=Oryza sativa subsp. japonica GN=ICL PE=1 SV=1	6.27
P0C5A4	Late embryogenesis abundant protein 19 OS=Oryza sativa subsp. japonica GN=LEA19 PE=2 SV=1	5.87
Q8H5Y7	Os07g0611400 protein OS=Oryza sativa subsp. japonica GN=OJ1003_H02.109 PE=4 SV=1	5.74
Q8S702	Glutathione S-transferase GSTU6, putative, expressed OS=Oryza sativa subsp. japonica GN=LOC_Os10g38470 PE=2 SV=1	5.08
Q7XUG1	Malate synthase OS=Oryza sativa subsp. japonica GN=MS PE=2 SV=1	4.73
Q2R1V2	Barwin, putative, expressed OS=Oryza sativa subsp. japonica GN=LOC_Os11g37950 PE=2 SV=1	4.41
Q2QLS9	Os12g0629700 protein OS=Oryza sativa subsp. japonica GN=LOC_Os12g43430 PE=2 SV=1	4.31
Q7XBA6	Non-specific lipid-transfer protein OS=Oryza sativa subsp. japonica GN=Os11g0115400 PE=2 SV=1	3.92
Q8H4P7	Os07g0147500 protein OS=Oryza sativa subsp. japonica GN=OJ1470_H06.117 PE=2 SV=1	3.90
Q84Q77	17.9 kDa class I heat shock protein OS=Oryza sativa subsp. japonica GN=HSP17.9A PE=1 SV=1	3.83
Q5VP66	Os01g0644000 protein OS=Oryza sativa subsp. japonica GN=Os01g0644000 PE=2 SV=1	3.82
Q306J3	Dirigent protein OS=Oryza sativa subsp. japonica GN=JAC1 PE=2 SV=1	3.72
A0A0P0WA:	Os04g0415800 protein OS=Oryza sativa subsp. japonica GN=Os04g0415800 PE=4 SV=1	3.67
A0A0P0VQF	Os02g0783625 protein OS=Oryza sativa subsp. japonica GN=Os02g0783625 PE=4 SV=1	3.66
Q84TB6	Actin-depolymerizing factor 3 OS=Oryza sativa subsp. japonica GN=ADF3 PE=1 SV=1	3.62
Q9FWU4	Os10g0491000 protein OS=Oryza sativa subsp. japonica GN=LOC_Os10g34930 PE=2 SV=1	3.55
Q42993	Chitinase 1 OS=Oryza sativa subsp. japonica GN=Cht1 PE=2 SV=1	3.50
A0A0P0XVP	Alpha-galactosidase OS=Oryza sativa subsp. japonica GN=Os10g0492900 PE=3 SV=1	3.46
Q0DPU1	Os03g0663500 protein (Fragment) OS=Oryza sativa subsp. japonica GN=Os03g0663500 PE=4 SV=1	3.39
Q94LP4	(RAP Annotation release2) 2OG-Fe(II) oxygenase domain containing protein OS=Oryza sativa subsp. japonica GN=OSJNBa0042H09.28 PE=2 SV=1	3.38
Q0DHF7	Os05g0468800 protein OS=Oryza sativa subsp. japonica GN=Os05g0468800 PE=4 SV=1	3.38

Q6ESR4	Dehydrin DHN1 OS=Oryza sativa subsp. japonica GN=DHN1 PE=2 SV=1	3.34
A0A0P0W1I	Os03g0661600 protein (Fragment) OS=Oryza sativa subsp. japonica GN=Os03g0661600 PE=4 SV=1	3.34
O22080	Acidic class III chitinase OsChib3a OS=Oryza sativa subsp. japonica GN=Os01g0660200 PE=2 SV=2	3.30
Q10N98	33 kDa secretory protein, putative, expressed OS=Oryza sativa subsp. japonica GN=Os03g0277600 PE=2 SV=1	3.28
Q6Z563	Os08g0412800 protein OS=Oryza sativa subsp. japonica GN=Os08g0412800 PE=2 SV=1	3.27
Q84Q72	18.1 kDa class I heat shock protein OS=Oryza sativa subsp. japonica GN=HSP18.1 PE=2 SV=1	3.26
Q7XIX0	Peroxidase OS=Oryza sativa subsp. japonica GN=OJ1167_G06.129 PE=3 SV=1	3.23
Q93WY5	Os09g0467200 protein OS=Oryza sativa subsp. japonica GN=Os09g0467200 PE=2 SV=1	3.19
B7EHD5	Os07g0523400 protein OS=Oryza sativa subsp. japonica GN=Os07g0523400 PE=2 SV=1	3.16
P27777	16.9 kDa class I heat shock protein 1 OS=Oryza sativa subsp. japonica GN=HSP16.9A PE=1 SV=1	3.06
Q0D5S1	Os07g0539900 protein OS=Oryza sativa subsp. japonica GN=Os07g0539900 PE=2 SV=1	3.04
A0A0P0WW	Os06g0323100 protein (Fragment) OS=Oryza sativa subsp. japonica GN=Os06g0323100 PE=4 SV=1	3.03
Q8GSD8	Os07g0290200 protein OS=Oryza sativa subsp. japonica GN=P0438G07.127 PE=2 SV=1	2.97
Q5VRY1	18.0 kDa class II heat shock protein OS=Oryza sativa subsp. japonica GN=HSP18.0 PE=2 SV=1	2.95
Q75GX3	Os03g0663400 protein OS=Oryza sativa subsp. japonica GN=Os03g0663400 PE=2 SV=1	2.94
Q10MK4	Mitochondrial import inner membrane translocase subunit Tim17/Tim22/Tim23 family protein, putative, expressed OS=Oryza sativa subsp. japonica GN=Os03g0305600 PE=2 SV=1	2.86
Q6YVU7	Os07g0539100 protein OS=Oryza sativa subsp. japonica GN=Os07g0539100 PE=2 SV=1	2.85
Q84YK8	Probable lipoxygenase 8, chloroplastic OS=Oryza sativa subsp. japonica GN=CM-LOX2 PE=2 SV=1	2.82
A5HEI2	Bowman-Birk type proteinase inhibitor A OS=Oryza sativa subsp. japonica GN=pinA PE=2 SV=1	2.82
A3BG40	Os07g0120600 protein OS=Oryza sativa subsp. japonica GN=Os07g0120650 PE=4 SV=1	2.80
P31110	Thaumatin-like protein OS=Oryza sativa subsp. japonica GN=Os12g0628600 PE=1 SV=1	2.78
Q8S9R1	Os01g0940800 protein OS=Oryza sativa subsp. japonica GN=Os01g0940800 PE=2 SV=1	2.74

Q8L4V6	Glutathione S-transferase GSTU6, putative, expressed OS=Oryza sativa subsp. japonica GN=LOC_Os10g38780 PE=2 SV=1	2.70
Q653Y0	Os06g0681200 protein OS=Oryza sativa subsp. japonica GN=Os06g0681200 PE=2 SV=1	2.68
Q8LNZ3	UDP-glucose 4-epimerase 1 OS=Oryza sativa subsp. japonica GN=UGE-1 PE=2 SV=1	2.67
Q7XDQ8	NAD dependent epimerase/dehydratase family protein, expressed OS=Oryza sativa subsp. japonica GN=LOC_Os10g31780 PE=4 SV=1	2.67
Q0JIK5	Os01g0795200 protein OS=Oryza sativa subsp. japonica GN=Os01g0795200 PE=3 SV=1	2.67
Q7F1U0	Peroxidase OS=Oryza sativa subsp. japonica GN=OJ1167_G06.125 PE=2 SV=1	2.65
Q652V8	16.0 kDa heat shock protein, peroxisomal OS=Oryza sativa subsp. japonica GN=HSP16.0 PE=2 SV=1	2.60
Q7F164	Os01g0940700 protein OS=Oryza sativa subsp. japonica GN=P0432C03.10-1 PE=2 SV=1	2.60
A0A0P0V8H	Os01g0757900 protein OS=Oryza sativa subsp. japonica GN=Os01g0757900 PE=4 SV=1	2.55
A0A0P0W6(	Os04g0107900 protein (Fragment) OS=Oryza sativa subsp. japonica GN=Os04g0107900 PE=4 SV=1	2.55
Q6ZLJ9	Os07g0209100 protein OS=Oryza sativa subsp. japonica GN=Os07g0209100 PE=4 SV=1	2.54
B7E9D7	Os01g0124650 protein OS=Oryza sativa subsp. japonica GN=Os01g0124650 PE=2 SV=1	2.53
Q0IZF1	Os09g0572700 protein OS=Oryza sativa subsp. japonica GN=Os09g0572700 PE=2 SV=1	2.48
Q2QNN5	Lipoxygenase OS=Oryza sativa subsp. japonica GN=Os12g0559200 PE=3 SV=2	2.48
Q9LGB2	Os01g0132000 protein OS=Oryza sativa subsp. japonica GN=Os01g0132000 PE=2 SV=1	2.48
Q75T45	Os12g0555000 protein OS=Oryza sativa subsp. japonica GN=RSOsPR10 PE=2 SV=1	2.47
Q9FP25	Os01g0303000 protein OS=Oryza sativa subsp. japonica GN=P0035H10.18 PE=2 SV=1	2.46
Q851F9	Probable zinc metalloprotease EGY3, chloroplastic OS=Oryza sativa subsp. japonica GN=EGY3 PE=2 SV=1	2.45
Q338P6	Os10g0389200 protein OS=Oryza sativa subsp. japonica GN=Os10g0389200 PE=2 SV=1	2.45
Q84J50	17.7 kDa class I heat shock protein OS=Oryza sativa subsp. japonica GN=HSP17.7 PE=2 SV=1	2.45
Q0IQK9	Non-specific lipid-transfer protein 1 OS=Oryza sativa subsp. japonica GN=LTP PE=1 SV=1	2.44
Q10NA1	Heat shock cognate 70 kDa protein, putative, expressed OS=Oryza sativa subsp. japonica GN=Os03g0277300 PE=3 SV=1	2.43
Q337E2	Expressed protein OS=Oryza sativa subsp. japonica GN=Os10g0505900 PE=4 SV=1	2.42

Q7X8R5	Thioredoxin M2, chloroplastic OS=Oryza sativa subsp. japonica GN=Os04g0530600 PE=2 SV=2	2.42
Q94E74	Os01g0511100 protein OS=Oryza sativa subsp. japonica GN=Os01g0511100 PE=2 SV=1	2.40
B9FCZ7	Os04g0663700 protein OS=Oryza sativa subsp. japonica GN=Os04g0663700 PE=4 SV=1	2.40
Q0D840	Thioredoxin H1 OS=Oryza sativa subsp. japonica GN=TRXH PE=1 SV=1	2.37
Q7F2P0	Os01g0382000 protein OS=Oryza sativa subsp. japonica GN=Os01g0382000 PE=3 SV=1	2.35
Q5ZCB1	Os01g0124000 protein OS=Oryza sativa subsp. japonica GN=Os01g0124000 PE=2 SV=1	2.35
Q6Z4I3	Thioredoxin H2-1 OS=Oryza sativa subsp. japonica GN=Os07g0190800 PE=2 SV=1	2.34
Q2TN87	Os04g0208200 protein OS=Oryza sativa subsp. japonica GN=Os04g0208200 PE=2 SV=1	2.33
A0A0P0XGD	Os08g0425800 protein OS=Oryza sativa subsp. japonica GN=Os08g0425800 PE=4 SV=1	2.32
P38419	Lipoxygenase 7, chloroplastic OS=Oryza sativa subsp. japonica GN=CM-LOX1 PE=2 SV=2	2.32
Q8S1C7	Cytochrome P450 (CYP72C)-like OS=Oryza sativa subsp. japonica GN=CYP72A32 PE=2 SV=1	2.31
Q10MJ5	CP12, putative, expressed OS=Oryza sativa subsp. japonica GN=LOC_Os03g19380 PE=2 SV=1	2.30
Q7XEL9	Chitinase 1, putative, expressed OS=Oryza sativa subsp. japonica GN=Os10g0416800 PE=2 SV=1	2.29
Q10S66	Chitinase 11 OS=Oryza sativa subsp. japonica GN=Cht11 PE=2 SV=1	2.27
Q6K7E9	18.6 kDa class III heat shock protein OS=Oryza sativa subsp. japonica GN=HSP18.6 PE=2 SV=1	2.26
Q10K80	Carboxypeptidase OS=Oryza sativa subsp. japonica GN=Os03g0393700 PE=3 SV=1	2.25
Q94JF2	Late embryogenesis abundant protein 14 OS=Oryza sativa subsp. japonica GN=LEA14 PE=2 SV=1	2.25
Q5ZAV6	Os01g0783600 protein OS=Oryza sativa subsp. japonica GN=Os01g0783600 PE=2 SV=1	2.24
Q6YXZ3	Os02g0139100 protein OS=Oryza sativa subsp. japonica GN=Os02g0139100 PE=2 SV=1	2.23
Q7XSN6	Germin-like protein 4-1 OS=Oryza sativa subsp. japonica GN=Os04g0617900 PE=2 SV=2	2.23
Q8W3C9	Os10g0101000 protein OS=Oryza sativa subsp. japonica GN=Os10g0101000 PE=4 SV=1	2.23

Q5VRH4	Homogentisate 1,2-dioxygenase OS=Oryza sativa subsp. japonica GN=HGO PE=2 SV=1	2.23
Q5VRJ8	Peroxisomal membrane protein 11-5 OS=Oryza sativa subsp. japonica GN=PEX11-5 PE=2 SV=1	2.23
Q6Z7V2	24.1 kDa heat shock protein, mitochondrial OS=Oryza sativa subsp. japonica GN=HSP24.1 PE=2 SV=1	2.22
Q6ZFM7	Os07g0582400 protein OS=Oryza sativa subsp. japonica GN=Os07g0582400 PE=3 SV=1	2.19
Q851K1	Germin-like protein 3-6 OS=Oryza sativa subsp. japonica GN=Os03g0694000 PE=2 SV=1	2.18
Q0JK51	Os01g0695800 protein (Fragment) OS=Oryza sativa subsp. japonica GN=Os01g0695800 PE=4 SV=1	2.17
Q655T1	Phosphoglycerate kinase OS=Oryza sativa subsp. japonica GN=Os06g0668200 PE=2 SV=1	2.17
Q8W1L6	Peroxisomal fatty acid beta-oxidation multifunctional protein OS=Oryza sativa subsp. japonica GN=MFP PE=1 SV=2	2.16
Q943K7	70 kDa heat shock protein OS=Oryza sativa subsp. japonica GN=Os01g0840100 PE=2 SV=1	2.16
Q0JNL7	Calmodulin-3 OS=Oryza sativa subsp. japonica GN=CAM3 PE=2 SV=1	2.16
Q9XFE4	Peptidylprolyl isomerase OS=Oryza sativa subsp. japonica GN=Os04g0352400 PE=2 SV=2	2.13
A0A0P0WAI	Os04g0423600 protein OS=Oryza sativa subsp. japonica GN=Os04g0423600 PE=4 SV=1	2.12
A0A0P0VCV	Os01g0949750 protein (Fragment) OS=Oryza sativa subsp. japonica GN=Os01g0949750 PE=4 SV=1	2.11
Q5SMV5	Carboxypeptidase OS=Oryza sativa subsp. japonica GN=Os06g0186400 PE=2 SV=1	2.11
O82807	Ubiquinol oxidase 1a, mitochondrial OS=Oryza sativa subsp. japonica GN=AOX1A PE=2 SV=1	2.10
Q8S1G9	Os01g0796400 protein OS=Oryza sativa subsp. japonica GN=P0699H05.27-1 PE=2 SV=1	2.10
Q10LP5	Sucrose synthase 4 OS=Oryza sativa subsp. japonica GN=SUS4 PE=2 SV=1	2.10
Q5N7Y1	Os01g0910900 protein OS=Oryza sativa subsp. japonica GN=Os01g0910900 PE=2 SV=1	2.08
Q6K623	Os02g0612900 protein OS=Oryza sativa subsp. japonica GN=Os02g0612900 PE=2 SV=1	2.08
Q69P84	Aldehyde dehydrogenase OS=Oryza sativa subsp. japonica GN=OJ1344_B01.27-1 PE=2 SV=1	2.07
Q6ATC1	Os05g0160300 protein OS=Oryza sativa subsp. japonica GN=Os05g0160300 PE=2 SV=1	2.06
Q94CS9	Probable aquaporin TIP1-2 OS=Oryza sativa subsp. japonica GN=TIP1-2 PE=2 SV=1	2.05

Q10QP0	Os03g0189400 protein OS=Oryza sativa subsp. japonica GN=LOC_Os03g08999 PE=2 SV=1	2.04
Q6ZK52	Os08g0127100 protein OS=Oryza sativa subsp. japonica GN=Os08g0127100 PE=2 SV=1	2.03
Q2QNV2	Fiber protein Fb19, putative, expressed OS=Oryza sativa subsp. japonica GN=LOC_Os12g36640 PE=2 SV=1	2.02
Q0E3L4	Os02g0168100 protein (Fragment) OS=Oryza sativa subsp. japonica GN=Os02g0168100 PE=4 SV=1	2.02
Q94CU3	Uricase OS=Oryza sativa subsp. japonica GN=P0423B08.43-1 PE=2 SV=1	2.01
Q7FAX1	Peroxygenase OS=Oryza sativa subsp. japonica GN=PXG PE=2 SV=1	2.01
Q75LD9	Os03g0843300 protein OS=Oryza sativa subsp. japonica GN=OSJNBa0032G11.5 PE=2 SV=1	2.01
Q7XNV4	OSJNBb0015G09.12 protein OS=Oryza sativa subsp. japonica GN=Os04g0227500 PE=2 SV=2	1.98
Q8H620	Os06g0176700 protein OS=Oryza sativa subsp. japonica GN=Os06g0176700 PE=3 SV=1	1.98
Q8S1M1	Embryonic abundant protein-like OS=Oryza sativa subsp. japonica GN=Os01g0716500 PE=2 SV=1	1.98
Q0J8R9	Os04g0690800 protein OS=Oryza sativa subsp. japonica GN=Os04g0690800 PE=4 SV=1	1.98
Q337M4	Os10g0463800 protein OS=Oryza sativa subsp. japonica GN=Os10g0463800 PE=2 SV=1	1.98
Q2QVJ8	NADP-dependent oxidoreductase P2, putative, expressed OS=Oryza sativa subsp. japonica GN=Os12g0226700 PE=4 SV=1	1.97
A0A0P0V4A	Os01g0571166 protein OS=Oryza sativa subsp. japonica GN=Os01g0571166 PE=4 SV=1	1.95
Q0DAI4	Os06g0651000 protein OS=Oryza sativa subsp. japonica GN=Os06g0651000 PE=4 SV=1	1.95
Q2QXL3	ACT domain-containing protein, putative, expressed OS=Oryza sativa subsp. japonica GN=Os12g0152700 PE=2 SV=1	1.94
Q6Z2G8	Os02g0705400 protein OS=Oryza sativa subsp. japonica GN=P0680A05.8-1 PE=2 SV=1	1.93
Q53K63	Prefoldin subunit 4 OS=Oryza sativa subsp. japonica GN=LOC_Os03g43020 PE=3 SV=1	1.93
Q75IR2	Os05g0163700 protein OS=Oryza sativa subsp. japonica GN=Os05g0163700 PE=2 SV=1	1.93
Q6F2Y7	Chaperone protein ClpB1 OS=Oryza sativa subsp. japonica GN=CLPB1 PE=2 SV=1	1.93
Q7X8W6	OSJNBa0081C01.20 protein OS=Oryza sativa subsp. japonica GN=Os04g0531900 PE=2 SV=2	1.93
Q7XKI7	Mitochondrial intermembrane space import and assembly protein 40 homolog OS=Oryza sativa subsp. japonica GN=MIA40 PE=2 SV=2	1.92
Q6J657	Cell death associated protein OS=Oryza sativa subsp. japonica GN=Os05g0410200 PE=2 SV=1	1.92
Q5JJV6	Os01g0965900 protein OS=Oryza sativa subsp. japonica GN=Os01g0965900 PE=2 SV=1	1.92

O49827	Chitinase OS=Oryza sativa subsp. japonica GN=Os01g0860500 PE=2 SV=1	1.91
Q7XKV4	Beta-glucosidase 12 OS=Oryza sativa subsp. japonica GN=BGLU12 PE=1 SV=2	1.90
Q8W2X5	Flavanone 3-dioxygenase 2 OS=Oryza sativa subsp. japonica GN=F3H-2 PE=1 SV=1	1.90
Q6L509	Os05g0460000 protein OS=Oryza sativa subsp. japonica GN=Os05g0460000 PE=2 SV=1	1.90
Q7Y0E8	Probable nucleoredoxin 1-1 OS=Oryza sativa subsp. japonica GN=Os03g0405500 PE=2 SV=1	1.89
Q2R429	Expressed protein OS=Oryza sativa subsp. japonica GN=Os11g0491400 PE=2 SV=1	1.89
Q6ZJ08	Monodehydroascorbate reductase 4, cytosolic OS=Oryza sativa subsp. japonica GN=MDAR4 PE=1 SV=1	1.89
Q8RU06	Cellulase containing protein, expressed OS=Oryza sativa subsp. japonica GN=OJ1208D02.5 PE=2 SV=1	1.89
Q9SXF8	Aquaporin PIP 1-3 OS=Oryza sativa subsp. japonica GN=PIP1-3 PE=2 SV=2	1.87
Q0DV66	Os03g0146400 protein OS=Oryza sativa subsp. japonica GN=Os03g0146400 PE=4 SV=1	1.85
Q658G9	Cytochrome P450 OS=Oryza sativa subsp. japonica GN=Os06g0129900 PE=2 SV=1	1.85
Q6ZCF3	Os08g0205400 protein OS=Oryza sativa subsp. japonica GN=Os08g0205400 PE=2 SV=1	1.85
Q7XN11	Gamma-aminobutyrate transaminase 1, mitochondrial OS=Oryza sativa subsp. japonica GN=OSL2 PE=1 SV=2	1.85
Q7EZD7	Sugar transport protein MST3 OS=Oryza sativa subsp. japonica GN=MST3 PE=2 SV=1	1.85
Q10PW9	Sugar transport protein MST4 OS=Oryza sativa subsp. japonica GN=MST4 PE=1 SV=1	1.84
Q9ZQT3	NB-ARC domain containing protein, expressed OS=Oryza sativa subsp. japonica GN=RPR1 PE=2 SV=1	1.83
Q6K7A3	Os02g0469600 protein OS=Oryza sativa subsp. japonica GN=Os02g0469600 PE=2 SV=1	1.83
Q69LD9	Os07g0169600 protein OS=Oryza sativa subsp. japonica GN=Os07g0169600 PE=2 SV=1	1.83
Q6YVU4	Os07g0539300 protein OS=Oryza sativa subsp. japonica GN=P0696F12.36-1 PE=2 SV=1	1.83
Q2QNT0	Os12g0555200 protein OS=Oryza sativa subsp. japonica GN=LOC_Os12g36850 PE=2 SV=1	1.83
Q2R5M2	Carboxypeptidase OS=Oryza sativa subsp. japonica GN=LOC_Os11g24510 PE=3 SV=1	1.82
Q42972	Malate dehydrogenase, glyoxysomal OS=Oryza sativa subsp. japonica GN=Os12g0632700 PE=1 SV=3	1.82
Q7XQ93	OSJNBa0018M05.15 protein OS=Oryza sativa subsp. japonica GN=Os04g0674700 PE=2 SV=2	1.82
Q5ZBR8	Os01g0795000 protein OS=Oryza sativa subsp. japonica GN=Os01g0795000 PE=4 SV=1	1.82
Q10PW8	DnaK protein, expressed OS=Oryza sativa subsp. japonica GN=Os03g0218500 PE=2 SV=1	1.81

Q6H7M1	Fumarylacetoacetase OS=Oryza sativa subsp. japonica GN=FAH PE=2 SV=1	1.81
A0A0P0VVF	Os03g0248200 protein (Fragment) OS=Oryza sativa subsp. japonica GN=Os03g0248200 PE=3 SV=1	1.80
P42211	Aspartic proteinase OS=Oryza sativa subsp. japonica GN=RAP PE=2 SV=2	1.80
Q8LHS0	Neurofilament triplet M protein-like protein OS=Oryza sativa subsp. japonica GN=P0039H02.109 PE=2 SV=1	1.80
Q0DGW8	Os05g0507300 protein (Fragment) OS=Oryza sativa subsp. japonica GN=Os05g0507300 PE=3 SV=1	1.80
Q0D9C4	Catalase isozyme B OS=Oryza sativa subsp. japonica GN=CATB PE=2 SV=1	1.80
Q10LF1	Acyl carrier protein OS=Oryza sativa subsp. japonica GN=Os03g0352800 PE=2 SV=1	1.80
Q7XW32	OSJNBb0062H02.10 protein OS=Oryza sativa subsp. japonica GN=Os04g0322100 PE=2 SV=2	1.80
Q5QMT0	Beta-glucosidase 1 OS=Oryza sativa subsp. japonica GN=BGLU1 PE=2 SV=1	1.80
Q94DD0	Os01g0859200 protein OS=Oryza sativa subsp. japonica GN=Os01g0859200 PE=2 SV=1	1.79
Q6ESZ6	Cytochrome b-c1 complex subunit 6 OS=Oryza sativa subsp. japonica GN=Os02g0541700 PE=3 SV=1	1.79
Q84P96	3-ketoacyl-CoA thiolase-like protein OS=Oryza sativa subsp. japonica GN=Os02g0817700 PE=2 SV=1	1.79
Q6K8R2	Chitinase 6 OS=Oryza sativa subsp. japonica GN=Cht6 PE=2 SV=1	1.79
Q9LGL5	Os01g0160100 protein OS=Oryza sativa subsp. japonica GN=Os01g0160100 PE=3 SV=1	1.79
Q9FEG7	Os08g0434100 protein OS=Oryza sativa subsp. japonica GN=Os08g0434100 PE=2 SV=1	1.79
Q2QN11	Eukaryotic aspartyl protease family protein, expressed OS=Oryza sativa subsp. japonica GN=Os12g0583300 PE=3 SV=1	1.78
Q2RAZ2	Alpha-L-arabinofuranosidase C-terminus family protein, expressed OS=Oryza sativa subsp. japonica GN=LOC_Os11g03780 PE=4 SV=1	1.78
Q10Q92	CUE domain containing protein, expressed OS=Oryza sativa subsp. japonica GN=Os03g0205000 PE=2 SV=1	1.77
C7J745	Os09g0491852 protein OS=Oryza sativa subsp. japonica GN=Os09g0491852 PE=4 SV=1	1.77
A0A0P0XNI[illegible]	Os09g0442300 protein (Fragment) OS=Oryza sativa subsp. japonica GN=Os09g0442300 PE=3 SV=1	1.76
Q75PK7	Os01g0315800 protein OS=Oryza sativa subsp. japonica GN=UXS-2 PE=2 SV=1	1.76
Q6YVX5	Chloride channel protein OS=Oryza sativa subsp. japonica GN=Os02g0558100 PE=3 SV=1	1.75
Q5ZAV7	Os01g0783500 protein OS=Oryza sativa subsp. japonica GN=Os01g0783500 PE=2 SV=1	1.75
Q5U1S8	Peroxidase OS=Oryza sativa subsp. japonica GN=prx15 PE=2 SV=1	1.74
A0A0N7KP2	Os07g0683600 protein OS=Oryza sativa subsp. japonica GN=Os07g0683600 PE=4 SV=1	1.74
Q75M67	Expressed protein OS=Oryza sativa subsp. japonica GN=Os03g0381300 PE=4 SV=1	1.74

Q5Z9N8	90 kDa heat shock protein OS=Oryza sativa subsp. japonica GN=Os06g0716700 PE=2 SV=1	1.74
Q84VG2	Os09g0425900 protein OS=Oryza sativa subsp. japonica GN=Os09g0425900 PE=2 SV=1	1.73
Q6H5Y1	Os02g0228300 protein OS=Oryza sativa subsp. japonica GN=Os02g0228300 PE=4 SV=1	1.72
A0A0P0V7Z	Os01g0740650 protein OS=Oryza sativa subsp. japonica GN=Os01g0740650 PE=4 SV=1	1.72
Q10G56	Ornithine aminotransferase, mitochondrial OS=Oryza sativa subsp. japonica GN=OAT PE=2 SV=1	1.72
Q8LQD2	Os01g0516600 protein OS=Oryza sativa subsp. japonica GN=B1108H10.11-1 PE=4 SV=1	1.72
Q7X7N2	Arginase 1, mitochondrial OS=Oryza sativa subsp. japonica GN=ARG1 PE=2 SV=1	1.72
Q69IN9	Os09g0512900 protein OS=Oryza sativa subsp. japonica GN=P0450E05.3-1 PE=2 SV=1	1.71
Q10RH3	Os03g0159100 protein OS=Oryza sativa subsp. japonica GN=Os03g0159100 PE=2 SV=1	1.71
Q6Z312	bZIP transcription factor 23 OS=Oryza sativa subsp. japonica GN=BZIP23 PE=2 SV=1	1.71
A0A0P0XCD	Os08g0174300 protein OS=Oryza sativa subsp. japonica GN=Os08g0174300 PE=4 SV=1	1.71
Q0JB49	Glutathione peroxidase OS=Oryza sativa subsp. japonica GN=Os04g0556300 PE=2 SV=1	1.70
B9FCW0	Os04g0652700 protein OS=Oryza sativa subsp. japonica GN=Os04g0652700 PE=4 SV=1	1.70
Q53NE7	Eukaryotic aspartyl protease family protein, expressed OS=Oryza sativa subsp. japonica GN=Os11g0183900 PE=3 SV=1	1.70
Q2QNS7	Os12g0555500 protein OS=Oryza sativa subsp. japonica GN=Os12g0555500 PE=2 SV=1	1.70
Q5VRM0	Acyl-CoA-binding domain-containing protein 2 OS=Oryza sativa subsp. japonica GN=ACBP2 PE=1 SV=1	1.70
Q2QZT1	Os11g0673100 protein OS=Oryza sativa subsp. japonica GN=Os11g0673100 PE=2 SV=1	1.70
Q6K439	Probable plastid-lipid-associated protein 2, chloroplastic OS=Oryza sativa subsp. japonica GN=PAP2 PE=2 SV=1	1.70
Q5W6C5	Carboxypeptidase OS=Oryza sativa subsp. japonica GN=Os05g0268500 PE=3 SV=1	1.70
Q60DX1	Thiamine pyrophosphokinase 3 OS=Oryza sativa subsp. japonica GN=TPK3 PE=2 SV=1	1.69
Q84PD0	DNAJ-like protein OS=Oryza sativa subsp. japonica GN=LOC_Os03g44620 PE=2 SV=1	1.69
Q5ZDH9	Os01g0139200 protein OS=Oryza sativa subsp. japonica GN=Os01g0139200 PE=4 SV=1	1.69
Q8H7P3	Expressed protein OS=Oryza sativa subsp. japonica GN=OJ1217B09.3 PE=2 SV=1	1.68
Q6Z6A7	Annexin OS=Oryza sativa subsp. japonica GN=Os02g0753800 PE=3 SV=1	1.68
Q5KQJ3	Os05g0181901 protein OS=Oryza sativa subsp. japonica GN=Os05g0182000 PE=4 SV=1	1.68

Q94DM2	Peroxidase OS=Oryza sativa subsp. japonica GN=prx22 PE=2 SV=1	1.67
Q69QQ2	Os09g0482200 protein OS=Oryza sativa subsp. japonica GN=Os09g0482500 PE=3 SV=1	1.67
Q6ZBH5	3-hydroxy-3-methylglutaryl coenzyme A synthase OS=Oryza sativa subsp. japonica GN=Os08g0544900 PE=2 SV=1	1.67
Q5Z9H8	Peptidyl-prolyl cis-trans isomerase OS=Oryza sativa subsp. japonica GN=P0655A07.5-2 PE=2 SV=1	1.66
P48642	Glutathione reductase, cytosolic OS=Oryza sativa subsp. japonica GN=GRC2 PE=2 SV=2	1.66
Q688X8	'putative heat shock protein, hsp40 OS=Oryza sativa subsp. japonica GN=Os05g0562300 PE=2 SV=1	1.66
Q75IM9	Isovaleryl-CoA dehydrogenase, mitochondrial OS=Oryza sativa subsp. japonica GN=Os05g0125500 PE=2 SV=2	1.66
Q75LJ3	Electron transfer flavoprotein subunit alpha, mitochondrial OS=Oryza sativa subsp. japonica GN=ETFA PE=2 SV=1	1.66
Q75HX0	Actin OS=Oryza sativa subsp. japonica GN=Os05g0438800 PE=2 SV=1	1.65
Q5ZEL4	Os01g0159400 protein OS=Oryza sativa subsp. japonica GN=Os01g0159400 PE=2 SV=1	1.65
Q0DQM3	Os03g0580200 protein (Fragment) OS=Oryza sativa subsp. japonica GN=Os03g0580200 PE=4 SV=1	1.65
Q6ITC5	Cell death-related protein OS=Oryza sativa subsp. japonica GN=A2 PE=2 SV=1	1.64
Q10PW2	Tubulin alpha chain OS=Oryza sativa subsp. japonica GN=TubA PE=2 SV=1	1.64
Q67WV5	Os06g0643000 protein OS=Oryza sativa subsp. japonica GN=Os06g0643000 PE=4 SV=1	1.64
Q67VZ1	Annexin OS=Oryza sativa subsp. japonica GN=Os06g0221200 PE=2 SV=1	1.64
Q10C96	Os03g0790500 protein OS=Oryza sativa subsp. japonica GN=LOC_Os03g57640 PE=2 SV=1	1.64
Q6Z481	Glycosyltransferase OS=Oryza sativa subsp. japonica GN=Os07g0503300 PE=2 SV=1	1.64
Q53LQ0	Protein disulfide isomerase-like 1-1 OS=Oryza sativa subsp. japonica GN=PDIL1-1 PE=2 SV=1	1.64
Q6Z8N9	Os08g0512400 protein OS=Oryza sativa subsp. japonica GN=P0711H09.10-2 PE=4 SV=1	1.63
Q6ZFJ9	60 kDa chaperonin beta subunit OS=Oryza sativa subsp. japonica GN=Os02g0102900 PE=2 SV=1	1.63
Q7XSC5	OSJNBa0027O01.6 protein OS=Oryza sativa subsp. japonica GN=Os04g0181100 PE=2 SV=2	1.63
A0A0P0WM	Os05g0432700 protein (Fragment) OS=Oryza sativa subsp. japonica GN=Os05g0432700 PE=4 SV=1	1.63
Q10T26	Os03g0103200 protein OS=Oryza sativa subsp. japonica GN=LOC_Os03g01310 PE=2 SV=1	1.63
Q8GVH2	Os07g0638100 protein OS=Oryza sativa subsp. japonica GN=OJ1340_C08.105 PE=4 SV=1	1.63
Q8H6G8	Probable inorganic phosphate transporter 1-8 OS=Oryza sativa subsp. japonica GN=PHT1-8 PE=2 SV=1	1.63
P49100	Cytochrome b5 OS=Oryza sativa subsp. japonica GN=Os05g0108800 PE=2 SV=2	1.63

Q7XUX4	OSJNBa0027P08.20 protein OS=Oryza sativa subsp. japonica GN=Os04g0446300 PE=2 SV=2	1.63
Q10MK9	AMP-binding enzyme family protein, expressed OS=Oryza sativa subsp. japonica GN=Os03g0305100 PE=4 SV=1	1.63
Q69XR7	Acyl-coenzyme A oxidase OS=Oryza sativa subsp. japonica GN=Os06g0354500 PE=2 SV=1	1.62
Q6K6I2	Os02g0473200 protein OS=Oryza sativa subsp. japonica GN=P0487H05.16-1 PE=3 SV=1	1.62
Q7XCK6	Chitinase 8 OS=Oryza sativa subsp. japonica GN=Cht8 PE=2 SV=1	1.62
Q0J4P2	Heat shock protein 81-1 OS=Oryza sativa subsp. japonica GN=HSP81-1 PE=3 SV=2	1.62
Q0JJQ7	Malic enzyme (Fragment) OS=Oryza sativa subsp. japonica GN=Os01g0723400 PE=3 SV=1	1.62
A0A0P0YAL:	Phenylalanine ammonia-lyase OS=Oryza sativa subsp. japonica GN=Os12g0520200 PE=3 SV=1	1.62
Q0DI31	Cytochrome c OS=Oryza sativa subsp. japonica GN=CC-1 PE=1 SV=1	1.62
Q9AT30	Chitinase 2, putative, expressed OS=Oryza sativa subsp. japonica GN=Rcb4 PE=2 SV=1	1.61
A3AQC6	Os04g0117800 protein OS=Oryza sativa subsp. japonica GN=Os04g0117900 PE=2 SV=1	1.61
A0A0P0UY0	Os01g0141100 protein OS=Oryza sativa subsp. japonica GN=Os01g0141100 PE=4 SV=1	1.61
Q5NA06	Coatomer subunit zeta-3 OS=Oryza sativa subsp. japonica GN=Os01g0838800 PE=2 SV=1	1.60
Q5Z414	Os06g0730800 protein OS=Oryza sativa subsp. japonica GN=Os06g0730800 PE=2 SV=1	1.60
Q5JMS4	Peroxidase OS=Oryza sativa subsp. japonica GN=Os01g0962700 PE=2 SV=1	1.60
Q6Z0R0	Os08g0455800 protein OS=Oryza sativa subsp. japonica GN=Os08g0455800 PE=2 SV=1	1.60
Q941F5	Os11g0592200 protein OS=Oryza sativa subsp. japonica GN=PR4 PE=2 SV=1	1.60
P09229	Cysteine proteinase inhibitor 1 OS=Oryza sativa subsp. japonica GN=Os01g0803200 PE=1 SV=2	1.60
Q7X6T8	Os07g0217600 protein OS=Oryza sativa subsp. japonica GN=OJ1080_F08.127 PE=2 SV=1	1.60
Q10PD0	Purple acid phosphatase OS=Oryza sativa subsp. japonica GN=LOC_Os03g13540 PE=2 SV=1	1.60
Q6Z244	Os08g0480200 protein OS=Oryza sativa subsp. japonica GN=Os08g0480200 PE=2 SV=1	1.60
Q06398	Probable glutathione S-transferase GSTU6 OS=Oryza sativa subsp. japonica GN=GSTU6 PE=2 SV=2	1.60
Q8H3A4	ABC transporter permease protein-like protein OS=Oryza sativa subsp. japonica GN=P0616D06.112-1 PE=2 SV=1	1.60
Q6I683	Os05g0363200 protein OS=Oryza sativa subsp. japonica GN=UXS-5 PE=2 SV=1	1.60
Q6ZHS1	Glycosyltransferase OS=Oryza sativa subsp. japonica GN=Os02g0188000 PE=3 SV=1	1.59
Q84SZ7	AAA1 OS=Oryza sativa subsp. japonica GN=AAA1 PE=2 SV=1	1.59

Q8S7M7	Plant intracellular Ras-group-related LRR protein 5 OS=Oryza sativa subsp. japonica GN=IRL5 PE=2 SV=1	1.59
Q5SN58	Os01g0664500 protein OS=Oryza sativa subsp. japonica GN=P0003E08.12-1 PE=2 SV=1	1.58
Q6H795	Chaperone protein ClpD1, chloroplastic OS=Oryza sativa subsp. japonica GN=CLPD1 PE=2 SV=1	1.58
Q6ESJ0	Glutathione peroxidase OS=Oryza sativa subsp. japonica GN=Os02g0664000 PE=2 SV=1	1.58
Q0J8A4	Glyceraldehyde-3-phosphate dehydrogenase 1, cytosolic OS=Oryza sativa subsp. japonica GN=GAPC1 PE=1 SV=1	1.58
Q6YX79	GHMP kinase-like protein OS=Oryza sativa subsp. japonica GN=Os02g0141300 PE=2 SV=1	1.58
Q7XQU7	Probable protein phosphatase 2C 41 OS=Oryza sativa subsp. japonica GN=Os04g0452000 PE=2 SV=2	1.58
Q7XLZ6	OSJNBa0086O06.13 protein OS=Oryza sativa subsp. japonica GN=Os04g0589900 PE=2 SV=2	1.58
Q8H8C7	Chitin elicitor-binding protein OS=Oryza sativa subsp. japonica GN=CEBIP PE=1 SV=1	1.57
Q5Z7I5	Os06g0548200 protein OS=Oryza sativa subsp. japonica GN=Os06g0548200 PE=3 SV=1	1.57
Q5QLQ5	Os01g0667200 protein OS=Oryza sativa subsp. japonica GN=Os01g0667200 PE=2 SV=1	1.57
Q6Z7B0	Heat shock 70 kDa protein BIP1 OS=Oryza sativa subsp. japonica GN=BIP1 PE=1 SV=1	1.57
Q6I605	Os05g0557200 protein OS=Oryza sativa subsp. japonica GN=Os05g0557200 PE=2 SV=1	1.57
Q5JKZ9	Os01g0952600 protein OS=Oryza sativa subsp. japonica GN=Os01g0952600 PE=3 SV=1	1.57
Q5NA77	C2 domain-containing protein-like OS=Oryza sativa subsp. japonica GN=Os01g0242600 PE=2 SV=1	1.57
Q9LDX7	Os01g0235300 protein OS=Oryza sativa subsp. japonica GN=Os01g0235300 PE=2 SV=1	1.57
Q650Z3	Os09g0572900 protein OS=Oryza sativa subsp. japonica GN=Os09g0572900 PE=3 SV=1	1.57
Q942L2	Protein disulfide isomerase-like 2-2 OS=Oryza sativa subsp. japonica GN=PDIL2-2 PE=2 SV=1	1.57
C7J6W5	Os09g0482660 protein (Fragment) OS=Oryza sativa subsp. japonica GN=Os09g0482660 PE=4 SV=1	1.57
Q53NG8	Aldehyde dehydrogenase OS=Oryza sativa subsp. japonica GN=Os11g0186200 PE=2 SV=1	1.56
A0A0P0W7[illegible]	Glycosyltransferase OS=Oryza sativa subsp. japonica GN=Os04g0206700 PE=3 SV=1	1.56
A0A0P0VG7	Os02g0207900 protein OS=Oryza sativa subsp. japonica GN=Os02g0207900 PE=4 SV=1	1.56
Q94DL7	Os01g0963600 protein OS=Oryza sativa subsp. japonica GN=Os01g0963600 PE=2 SV=1	1.56
Q6L4Z4	Os05g0462400 protein OS=Oryza sativa subsp. japonica GN=Os05g0462400 PE=4 SV=1	1.56
Q6ZKI2	Os08g0139000 protein OS=Oryza sativa subsp. japonica GN=OJ1119_D01.17-1 PE=2 SV=1	1.56

Q0J2B5	Aminopeptidase M1-C OS=Oryza sativa subsp. japonica GN=Os09g0362500 PE=2 SV=2	1.56
Q52RG7	Sphingosine-1-phosphate lyase OS=Oryza sativa subsp. japonica GN=SPL PE=2 SV=3	1.55
Q6K6X2	Os02g0610400 protein OS=Oryza sativa subsp. japonica GN=Os02g0610400 PE=2 SV=1	1.55
Q2R1S1	Harpin binding protein 1, putative, expressed OS=Oryza sativa subsp. japonica GN=LOC_Os11g38260 PE=2 SV=1	1.55
Q7XUP7	Peptide methionine sulfoxide reductase A2-1 OS=Oryza sativa subsp. japonica GN=MSRA2-1 PE=2 SV=2	1.55
C7J895	Os11g0226933 protein (Fragment) OS=Oryza sativa subsp. japonica GN=Os11g0226933 PE=4 SV=1	1.55
Q5KQK6	Os05g0179800 protein OS=Oryza sativa subsp. japonica GN=Os05g0179800 PE=4 SV=1	1.55
Q5VRZ6	Os06g0112400 protein OS=Oryza sativa subsp. japonica GN=Os06g0112400 PE=2 SV=1	1.55
Q0ILB9	Glycerol kinase-like protein OS=Oryza sativa subsp. japonica GN=OGK1 PE=2 SV=1	1.55
Q2QNQ6	Expressed protein OS=Oryza sativa subsp. japonica GN=Os12g0557400 PE=2 SV=1	1.55
Q8S718	Glutathione S-transferase GSTU6, putative, expressed OS=Oryza sativa subsp. japonica GN=Os10g0530400 PE=2 SV=1	1.55
Q8RU26	Os01g0687400 protein OS=Oryza sativa subsp. japonica GN=Os01g0687400 PE=2 SV=1	1.54
A0A0P0W3Z	Os03g0750100 protein OS=Oryza sativa subsp. japonica GN=Os03g0750100 PE=4 SV=1	1.54
Q0JIL1	Probable nucleoredoxin 2 OS=Oryza sativa subsp. japonica GN=Os01g0794400 PE=2 SV=1	1.54
Q8H4K6	Reticulon-like protein OS=Oryza sativa subsp. japonica GN=OJ1351_C05.119 PE=2 SV=1	1.54
Q75L18	Os05g0112800 protein OS=Oryza sativa subsp. japonica GN=Os05g0112800 PE=2 SV=1	1.54
Q6YZX6	Putative aconitate hydratase, cytoplasmic OS=Oryza sativa subsp. japonica GN=Os08g0191100 PE=3 SV=1	1.54
Q6YW09	Os08g0250700 protein OS=Oryza sativa subsp. japonica GN=Os08g0250700 PE=2 SV=1	1.54
Q9FDZ1	Cytochrome P450 OS=Oryza sativa subsp. japonica GN=CYP72A18 PE=2 SV=1	1.54
Q6K5Q1	Peptidylprolyl isomerase OS=Oryza sativa subsp. japonica GN=Os02g0491400 PE=4 SV=1	1.54
Q9SXV0	Cytochrome c oxidase subunit 6b OS=Oryza sativa subsp. japonica GN=COX6b-1 PE=2 SV=1	1.53
Q7XWU3	Probable cinnamyl alcohol dehydrogenase 6 OS=Oryza sativa subsp. japonica GN=CAD6 PE=2 SV=2	1.53
Q6H7R1	Os02g0643000 protein OS=Oryza sativa subsp. japonica GN=Os02g0643000 PE=2 SV=1	1.53
Q84P62	OSJNBa0087O24.10 protein OS=Oryza sativa subsp. japonica GN=Os04g0665800 PE=2 SV=1	1.53
Q10MN2	Peroxisomal membrane protein 11-3 OS=Oryza sativa subsp. japonica GN=PEX11-3 PE=2 SV=1	1.53

Q6AUV1	Xanthine dehydrogenase OS=Oryza sativa subsp. japonica GN=XDH PE=2 SV=1	1.53
Q2QWZ9	Transmembrane 9 superfamily member OS=Oryza sativa subsp. japonica GN=LOC_Os12g07670 PE=2 SV=1	1.52
Q0DLG8	Os05g0103100 protein OS=Oryza sativa subsp. japonica GN=Os05g0103100 PE=4 SV=1	1.52
Q75IC7	Secretory carrier-associated membrane protein 4 OS=Oryza sativa subsp. japonica GN=SCAMP4 PE=2 SV=1	1.52
Q6Z6L4	Aminopeptidase M1-A OS=Oryza sativa subsp. japonica GN=Os02g0218200 PE=2 SV=1	1.52
Q657Y8	Protein RER1 OS=Oryza sativa subsp. japonica GN=Os01g0106200 PE=2 SV=1	1.52
Q75M70	Expressed protein OS=Oryza sativa subsp. japonica GN=Os03g0381500 PE=2 SV=1	1.52
Q10BU2	Germin-like protein 3-7 OS=Oryza sativa subsp. japonica GN=GER7 PE=2 SV=1	1.52
Q0E4K1	Catalase isozyme A OS=Oryza sativa subsp. japonica GN=CATA PE=2 SV=1	1.52
Q6ZBK6	Os08g0519400 protein OS=Oryza sativa subsp. japonica GN=Os08g0519400 PE=4 SV=1	1.51
Q6AUF2	Os05g0565400 protein OS=Oryza sativa subsp. japonica GN=Os05g0565400 PE=2 SV=1	1.51
Q8H3C9	IAA-amino acid hydrolase ILR1-like 7 OS=Oryza sativa subsp. japonica GN=ILL7 PE=2 SV=1	1.51
Q7XSU8	Peroxidase OS=Oryza sativa subsp. japonica GN=Os04g0688300 PE=2 SV=2	1.51
Q7XD86	Os10g0486900 protein OS=Oryza sativa subsp. japonica GN=Os10g0486900 PE=2 SV=2	1.51
A0A0P0VBP	Os01g0895600 protein OS=Oryza sativa subsp. japonica GN=Os01g0895600 PE=3 SV=1	1.51
Q0ILZ4	DEAD-box ATP-dependent RNA helicase 9 OS=Oryza sativa subsp. japonica GN=Os12g0611200 PE=2 SV=1	1.51
Q10SR3	70 kDa heat shock protein OS=Oryza sativa subsp. japonica GN=Os03g0113700 PE=2 SV=1	1.51
Q94DM8	Ubiquitin-fold modifier 1 OS=Oryza sativa subsp. japonica GN=Os01g0962400 PE=3 SV=1	1.50
Q9XGP7	Tricin synthase 1 OS=Oryza sativa subsp. japonica GN=ROMT-15 PE=1 SV=1	1.50
Q5W6F1	Cinnamate-4-hydroxylase OS=Oryza sativa subsp. japonica GN=OsC4HL PE=2 SV=1	1.50
Q6Z8U4	Os08g0492100 protein OS=Oryza sativa subsp. japonica GN=Os08g0492100 PE=2 SV=1	1.50

NIP_ S2 vs C0_ Proteins decreased in abundance

Accession	Description	FC	
O64437	Inositol-3-phosphate synthase 1 OS=Oryza sativa subsp. japonica GN=RINO1 PE=1 SV=2	0.39	-2.53
Q5NAM3	Branched-chain amino acid aminotransferase-like OS=Oryza sativa subsp. japonica GN=Os01g0238500 PE=4 SV=1	0.41	-2.44
Q0IPL3	Os12g0189300 protein OS=Oryza sativa subsp. japonica GN=Os12g0189300 PE=4 SV=1	0.44	-2.28
Q7XI92	Os07g0580900 protein OS=Oryza sativa subsp. japonica GN=Os07g0580900 PE=1 SV=1	0.44	-2.26
Q5VME5	Glycosyltransferase OS=Oryza sativa subsp. japonica GN=Os06g0289900 PE=2 SV=1	0.45	-2.24
Q6K826	Os02g0781400 protein OS=Oryza sativa subsp. japonica GN=OJ1369_G08.10-1 PE=2 SV=1	0.45	-2.23
Q6ATB2	Probable GTP diphosphokinase CRSH2, chloroplastic OS=Oryza sativa subsp. japonica GN=CRSH2 PE=2 SV=1	0.46	-2.18
Q84JG9	NAD dependent epimerase/dehydratase family protein, expressed OS=Oryza sativa subsp. japonica GN=OJ1754_E06.1 PE=4 SV=1	0.46	-2.18
Q6ET88	Os02g0668100 protein OS=Oryza sativa subsp. japonica GN=Os02g0668100 PE=1 SV=1	0.46	-2.18
Q0DWE8	Os02g0817900 protein OS=Oryza sativa subsp. japonica GN=Os02g0817900 PE=3 SV=1	0.46	-2.16
Q10J01	Expressed protein OS=Oryza sativa subsp. japonica GN=Os03g0439700 PE=4 SV=1	0.46	-2.16
Q9FTN6	Os01g0106300 protein OS=Oryza sativa subsp. japonica GN=Os01g0106300 PE=2 SV=1	0.47	-2.11
Q10M50	Magnesium-chelatase subunit ChlH, chloroplastic OS=Oryza sativa subsp. japonica GN=CHLH PE=1 SV=1	0.48	-2.09
Q0DZN5	Os02g0608600 protein (Fragment) OS=Oryza sativa subsp. japonica GN=Os02g0608600 PE=4 SV=1	0.49	-2.06
Q8W3D9	Protochlorophyllide reductase B, chloroplastic OS=Oryza sativa subsp. japonica GN=PORB PE=2 SV=1	0.49	-2.05
Q6KA61	Os02g0285800 protein OS=Oryza sativa subsp. japonica GN=Os02g0285800 PE=4 SV=1	0.50	-2.01
Q6YY42	Os02g0589000 protein OS=Oryza sativa subsp. japonica GN=Os02g0589000 PE=4 SV=1	0.50	-1.99
B7EBJ6	Os08g0130400 protein OS=Oryza sativa subsp. japonica GN=Os08g0130400 PE=2 SV=1	0.51	-1.96
Q8LLP6	Expressed protein OS=Oryza sativa subsp. japonica GN=OSJNBa0031O09.02 PE=2 SV=1	0.51	-1.95
Q5QLS1	Arabinogalactan protein-like OS=Oryza sativa subsp. japonica GN=Os01g0668100 PE=2 SV=1	0.51	-1.94
Q6ZI95	Purple acid phosphatase OS=Oryza sativa subsp. japonica GN=Os08g0531000 PE=2 SV=1	0.52	-1.93

Q33AG7	CMV 1a interacting protein 1, putative, expressed OS=Oryza sativa subsp. japonica GN=Os10g0181600 PE=2 SV=2	0.52	-1.92
Q6H6D2	Porphobilinogen deaminase, chloroplastic OS=Oryza sativa subsp. japonica GN=HEMC PE=2 SV=1	0.52	-1.92
Q0DSS9	Os03g0290300 protein (Fragment) OS=Oryza sativa subsp. japonica GN=Os03g0290300 PE=4 SV=1	0.52	-1.92
Q10LR9	Uroporphyrinogen decarboxylase 2, chloroplastic OS=Oryza sativa subsp. japonica GN=Os03g0337600 PE=3 SV=1	0.53	-1.88
A0A0N7KEB	Os01g0919900 protein OS=Oryza sativa subsp. japonica GN=Os01g0919900 PE=4 SV=1	0.53	-1.87
Q6ZLK8	Os07g0134000 protein OS=Oryza sativa subsp. japonica GN=OJ1118_D07.26-1 PE=2 SV=1	0.54	-1.85
Q6Z1Y9	Os08g0101700 protein OS=Oryza sativa subsp. japonica GN=Os08g0101700 PE=2 SV=1	0.54	-1.85
Q0D5I5	Os07g0558300 protein OS=Oryza sativa subsp. japonica GN=Os07g0558300 PE=2 SV=1	0.54	-1.84
Q53RM0	Magnesium-chelatase subunit ChlI, chloroplastic OS=Oryza sativa subsp. japonica GN=CHLI PE=1 SV=1	0.55	-1.83
Q7XUY5	OSJNBb0048E02.12 protein OS=Oryza sativa subsp. japonica GN=Os04g0465600 PE=2 SV=1	0.55	-1.83
Q6ZGW6	Delta-12 fatty acid desaturase OS=Oryza sativa subsp. japonica GN=FAD2 PE=2 SV=1	0.56	-1.79
A0A0P0Y57	Os11g0673200 protein OS=Oryza sativa subsp. japonica GN=Os11g0673200 PE=4 SV=1	0.56	-1.79
A0A0N7KSP	Os11g0237700 protein OS=Oryza sativa subsp. japonica GN=Os11g0237700 PE=4 SV=1	0.56	-1.78
Q5ZC82	Cytokinin riboside 5'-monophosphate phosphoribohydrolase LOG OS=Oryza sativa subsp. japonica GN=LOG PE=1 SV=1	0.56	-1.77
A0A0P0Y8U	Os12g0263000 protein (Fragment) OS=Oryza sativa subsp. japonica GN=Os12g0263000 PE=4 SV=1	0.57	-1.77
Q8LHN4	Os07g0631900 protein OS=Oryza sativa subsp. japonica GN=P0519E12.113 PE=4 SV=1	0.57	-1.77
A0A0P0X2H	Os07g0158300 protein (Fragment) OS=Oryza sativa subsp. japonica GN=Os07g0158300 PE=4 SV=1	0.57	-1.76
P0C587	Glutamyl-tRNA reductase, chloroplastic OS=Oryza sativa subsp. japonica GN=Os10g0502400 PE=2 SV=1	0.57	-1.76
Q10SD2	Expressed protein OS=Oryza sativa subsp. japonica GN=Os03g0126300 PE=2 SV=1	0.57	-1.75
Q7XN02	OSJNBb0038F03.9 protein OS=Oryza sativa subsp. japonica GN=OSJNBb0038F03.9 PE=4 SV=1	0.57	-1.75
Q7F1H3	Blue copper-binding protein-like OS=Oryza sativa subsp. japonica GN=OJ1118_G09.114 PE=2 SV=1	0.57	-1.75
Q0JJS8	Fe-S cluster assembly factor HCF101, chloroplastic OS=Oryza sativa subsp. japonica GN=HCF101 PE=3 SV=3	0.58	-1.74

Q75IY5	Expressed protein OS=Oryza sativa subsp. japonica GN=LOC_Os03g30092 PE=2 SV=1	0.58	-1.74
Q9AXB0	Uroporphyrinogen decarboxylase 1, chloroplastic OS=Oryza sativa subsp. japonica GN=Os01g0622300 PE=2 SV=1	0.58	-1.73
Q0E243	Os02g0273100 protein OS=Oryza sativa subsp. japonica GN=Os02g0273100 PE=2 SV=1	0.58	-1.72
Q5Z8V9	Delta-aminolevulinic acid dehydratase, chloroplastic OS=Oryza sativa subsp. japonica GN=HEMB PE=2 SV=1	0.58	-1.71
Q10LH0	Divinyl chlorophyllide a 8-vinyl-reductase, chloroplastic OS=Oryza sativa subsp. japonica GN=DVR PE=3 SV=1	0.58	-1.71
Q75LC0	Os03g0844900 protein OS=Oryza sativa subsp. japonica GN=OSJNBa0032G11.21 PE=2 SV=1	0.59	-1.71
A0A0P0XZ68	Os11g0153600 protein OS=Oryza sativa subsp. japonica GN=Os11g0153600 PE=4 SV=1	0.59	-1.69
B7FA34	Os05g0548900 protein OS=Oryza sativa subsp. japonica GN=Os05g0548900 PE=2 SV=1	0.59	-1.69
Q69S79	Os02g0575500 protein OS=Oryza sativa subsp. japonica GN=P0703B01.21-1 PE=4 SV=1	0.60	-1.67
Q2QND9	Expressed protein OS=Oryza sativa subsp. japonica GN=Os12g0569200 PE=4 SV=1	0.60	-1.67
Q84P94	OSJNBa0043A12.15 protein OS=Oryza sativa subsp. japonica GN=Os04g0668800 PE=2 SV=1	0.60	-1.67
B7EIQ8	Os02g0125700 protein OS=Oryza sativa subsp. japonica GN=Os02g0125700 PE=2 SV=1	0.60	-1.67
Q6K209	Os02g0629800 protein OS=Oryza sativa subsp. japonica GN=Os02g0629800 PE=3 SV=1	0.60	-1.66
B7EYZ0	Os05g0456300 protein OS=Oryza sativa subsp. japonica GN=Os05g0456300 PE=2 SV=1	0.60	-1.66
Q2QW43	Expressed protein OS=Oryza sativa subsp. japonica GN=Os12g0209000 PE=4 SV=1	0.60	-1.65
Q2QYE1	Probable apyrase 3 OS=Oryza sativa subsp. japonica GN=APY3 PE=2 SV=2	0.61	-1.65
Q8LR33	Os01g0662700 protein OS=Oryza sativa subsp. japonica GN=Os01g0662700 PE=3 SV=1	0.61	-1.65
Q84QV5	Os08g0525700 protein OS=Oryza sativa subsp. japonica GN=OJ1191_A10.120 PE=4 SV=1	0.61	-1.65
Q84QW4	Os08g0524400 protein OS=Oryza sativa subsp. japonica GN=OJ1191_A10.104 PE=4 SV=1	0.61	-1.64
Q9FTN5	Os01g0106400 protein OS=Oryza sativa subsp. japonica GN=Os01g0106400 PE=2 SV=1	0.61	-1.64
Q84S01	Os08g0152700 protein OS=Oryza sativa subsp. japonica GN=OJ1349_D05.118 PE=2 SV=1	0.61	-1.64
Q69MM2	HMG type nucleosome/chromatin assembly factor OS=Oryza sativa subsp. japonica GN=Os09g0551600 PE=2 SV=1	0.61	-1.63
Q6ZCP8	Os08g0167500 protein OS=Oryza sativa subsp. japonica GN=Os08g0167500 PE=2 SV=1	0.61	-1.63

Q6ATS0	Magnesium-chelatase subunit ChlD, chloroplastic OS=Oryza sativa subsp. japonica GN=CHLD PE=1 SV=1	0.61	-1.63
Q7XTF0	OSJNBa0072F16.18 protein OS=Oryza sativa subsp. japonica GN=Os04g0462300 PE=4 SV=2	0.61	-1.63
Q6ZH05	Os02g0687900 protein OS=Oryza sativa subsp. japonica GN=Os02g0687900 PE=2 SV=1	0.61	-1.63
Q8W2X4	Expressed protein OS=Oryza sativa subsp. japonica GN=OSJNBb0060I05.14 PE=2 SV=1	0.62	-1.62
Q6YZE2	Glutamate-1-semialdehyde 2,1-aminomutase, chloroplastic OS=Oryza sativa subsp. japonica GN=GSA PE=2 SV=1	0.62	-1.62
Q6Z2T6	Geranylgeranyl diphosphate reductase, chloroplastic OS=Oryza sativa subsp. japonica GN=CHLP PE=2 SV=1	0.62	-1.62
Q10LT2	Expressed protein OS=Oryza sativa subsp. japonica GN=LOC_Os03g21780 PE=2 SV=1	0.62	-1.61
Q0IS49	Terpene cyclase/mutase family member OS=Oryza sativa subsp. japonica GN=Os11g0562100 PE=3 SV=1	0.62	-1.60
Q7Y168	Expressed protein OS=Oryza sativa subsp. japonica GN=Os03g0375200 PE=4 SV=1	0.62	-1.60
Q8GTK0	Starch synthase, chloroplastic/amyloplastic OS=Oryza sativa subsp. japonica GN=P0710F09.134 PE=3 SV=1	0.63	-1.60
Q6AVL0	Os03g0602600 protein OS=Oryza sativa subsp. japonica GN=OJ1519_A12.11 PE=4 SV=1	0.63	-1.60
A0A0P0X4N[illegible]	Os07g0243150 protein (Fragment) OS=Oryza sativa subsp. japonica GN=Os07g0243150 PE=4 SV=1	0.63	-1.59
O22567	1-deoxy-D-xylulose-5-phosphate synthase 1, chloroplastic OS=Oryza sativa subsp. japonica GN=CLA1 PE=2 SV=2	0.63	-1.59
Q69LG7	Aspartate kinase-homoserine dehydrogenase OS=Oryza sativa subsp. japonica GN=Os09g0294000 PE=2 SV=1	0.63	-1.59
Q9AX68	Os01g0611000 protein OS=Oryza sativa subsp. japonica GN=P0410E03.33-1 PE=2 SV=1	0.63	-1.59
Q0DAQ8	Os06g0638200 protein (Fragment) OS=Oryza sativa subsp. japonica GN=Os06g0638200 PE=4 SV=1	0.63	-1.58
A0A0P0WS8	Os06g0146300 protein (Fragment) OS=Oryza sativa subsp. japonica GN=Os06g0146300 PE=4 SV=1	0.63	-1.58
P0C464	30S ribosomal protein S11, chloroplastic OS=Oryza sativa subsp. japonica GN=rps11 PE=3 SV=1	0.63	-1.58
Q5ZAJ0	Respiratory burst oxidase homolog protein B OS=Oryza sativa subsp. japonica GN=RBOHB PE=1 SV=1	0.63	-1.58
Q5TKG2	Os05g0594500 protein OS=Oryza sativa subsp. japonica GN=Os05g0594500 PE=4 SV=1	0.64	-1.57
Q6ZFE5	Os08g0483200 protein OS=Oryza sativa subsp. japonica GN=OJ1111_H02.19-1 PE=2 SV=1	0.64	-1.56

Q7XPL2	Oxygen-dependent coproporphyrinogen-III oxidase, chloroplastic OS=Oryza sativa subsp. japonica GN=CPX PE=2 SV=2	0.64	-1.56
A0A0N7KJF4	Os04g0538166 protein (Fragment) OS=Oryza sativa subsp. japonica GN=Os04g0538166 PE=4 SV=1	0.64	-1.56
Q2QP54	Elongation factor Ts, mitochondrial OS=Oryza sativa subsp. japonica GN=EFTS PE=3 SV=1	0.64	-1.56
Q336V3	Cytochrome P450 family protein, expressed OS=Oryza sativa subsp. japonica GN=LOC_Os10g39930 PE=2 SV=1	0.64	-1.55
Q7X7Y5	OSJNBb0062H02.3 protein OS=Oryza sativa subsp. japonica GN=Os04g0321700 PE=2 SV=2	0.64	-1.55
Q336T5	Expansin-B3 OS=Oryza sativa subsp. japonica GN=EXPB3 PE=2 SV=2	0.64	-1.55
Q0J0M2	Acyl-[acyl-carrier-protein] hydrolase (Fragment) OS=Oryza sativa subsp. japonica GN=Os09g0505300 PE=3 SV=1	0.64	-1.55
Q6K4S7	Os02g0285300 protein OS=Oryza sativa subsp. japonica GN=Os02g0285300 PE=2 SV=1	0.65	-1.55
Q8H4Z0	Os07g0184800 protein OS=Oryza sativa subsp. japonica GN=OJ1046_F10.127 PE=2 SV=1	0.65	-1.55
Q0E3V2	Os02g0152900 protein OS=Oryza sativa subsp. japonica GN=Os02g0152900 PE=2 SV=1	0.65	-1.55
Q6Z314	Os02g0766000 protein OS=Oryza sativa subsp. japonica GN=OJ1004_A11.16-1 PE=2 SV=1	0.65	-1.55
Q0JE32	Probable aldo-keto reductase 1 OS=Oryza sativa subsp. japonica GN=Os04g0337500 PE=2 SV=1	0.65	-1.55
Q0DIP2	Os05g0373400 protein OS=Oryza sativa subsp. japonica GN=Os05g0373400 PE=2 SV=2	0.65	-1.55
Q8W250	1-deoxy-D-xylulose 5-phosphate reductoisomerase, chloroplastic OS=Oryza sativa subsp. japonica GN=DXR PE=2 SV=2	0.65	-1.54
Q5Z9S8	ABC transporter G family member 42 OS=Oryza sativa subsp. japonica GN=ABCG42 PE=2 SV=1	0.65	-1.53
Q5Z4M6	Os06g0308000 protein OS=Oryza sativa subsp. japonica GN=Os06g0308000 PE=2 SV=1	0.65	-1.53
Q0E2A9	Os02g0255700 protein OS=Oryza sativa subsp. japonica GN=Os02g0255700 PE=4 SV=1	0.65	-1.53
Q7F9I1	Chaperone protein ClpC1, chloroplastic OS=Oryza sativa subsp. japonica GN=CLPC1 PE=2 SV=2	0.66	-1.52
Q7F9Y6	OSJNBa0086O06.22 protein OS=Oryza sativa subsp. japonica GN=Os04g0591000 PE=2 SV=1	0.66	-1.52
Q7XS47	OSJNBa0035M09.17 protein OS=Oryza sativa subsp. japonica GN=Os04g0607000 PE=2 SV=2	0.66	-1.52
Q0IRY4	Os11g0586300 protein (Fragment) OS=Oryza sativa subsp. japonica GN=Os11g0586300 PE=4 SV=1	0.66	-1.51
P0CD22	NAD(P)H-quinone oxidoreductase subunit 2 A, chloroplastic OS=Oryza sativa subsp. japonica GN=ndhB1 PE=2 SV=1	0.66	-1.51

B9FBM2	Os03g0176600 protein OS=Oryza sativa subsp. japonica GN=Os03g0176700 PE=4 SV=1	0.66	-1.51
Q10DD6	Homeodomain protein JUBEL1, putative, expressed OS=Oryza sativa subsp. japonica GN=Os03g0732100 PE=4 SV=1	0.66	-1.51
Q5VPQ6	Os06g0119600 protein OS=Oryza sativa subsp. japonica GN=Os06g0119600 PE=2 SV=1	0.66	-1.51
Q8S091	Thioredoxin F, chloroplastic OS=Oryza sativa subsp. japonica GN=Os01g0913000 PE=2 SV=1	0.66	-1.50
Q5SNH7	Os01g0191100 protein OS=Oryza sativa subsp. japonica GN=Os01g0191100 PE=2 SV=1	0.67	-1.50
Q6YVH6	Hydrolase, alpha/beta fold family-like OS=Oryza sativa subsp. japonica GN=Os02g0705100 PE=2 SV=1	0.67	-1.50

NIP_ S4 vs C0_ Proteins increased in abundance

Accession	Description	FC
Q7XBA6	Non-specific lipid-transfer protein OS=Oryza sativa subsp. japonica GN=Os11g0115400 PE=2 SV=1	4.67
Q5VRY1	18.0 kDa class II heat shock protein OS=Oryza sativa subsp. japonica GN=HSP18.0 PE=2 SV=1	4.59
P27777	16.9 kDa class I heat shock protein 1 OS=Oryza sativa subsp. japonica GN=HSP16.9A PE=1 SV=1	4.53
Q84Q77	17.9 kDa class I heat shock protein OS=Oryza sativa subsp. japonica GN=HSP17.9A PE=1 SV=1	4.31
Q6K7E9	18.6 kDa class III heat shock protein OS=Oryza sativa subsp. japonica GN=HSP18.6 PE=2 SV=1	4.10
P0C5A4	Late embryogenesis abundant protein 19 OS=Oryza sativa subsp. japonica GN=LEA19 PE=2 SV=1	4.03
Q84Q72	18.1 kDa class I heat shock protein OS=Oryza sativa subsp. japonica GN=HSP18.1 PE=2 SV=1	4.00
Q652V8	16.0 kDa heat shock protein, peroxisomal OS=Oryza sativa subsp. japonica GN=HSP16.0 PE=2 SV=1	3.91
Q8H4P7	Os07g0147500 protein OS=Oryza sativa subsp. japonica GN=OJ1470_H06.117 PE=2 SV=1	3.83
Q0J8R9	Os04g0690800 protein OS=Oryza sativa subsp. japonica GN=Os04g0690800 PE=4 SV=1	3.74
Q7X8R5	Thioredoxin M2, chloroplastic OS=Oryza sativa subsp. japonica GN=Os04g0530600 PE=2 SV=2	3.72
Q84J50	17.7 kDa class I heat shock protein OS=Oryza sativa subsp. japonica GN=HSP17.7 PE=2 SV=1	3.46
Q10MK4	Mitochondrial import inner membrane translocase subunit Tim17/Tim22/Tim23 family protein, putative, expressed OS=Oryza sativa subsp. japonica GN=Os03g0305600 PE=2 SV=1	3.42
Q84TB6	Actin-depolymerizing factor 3 OS=Oryza sativa subsp. japonica GN=ADF3 PE=1 SV=1	3.39
Q10NA1	Heat shock cognate 70 kDa protein, putative, expressed OS=Oryza sativa subsp. japonica GN=Os03g0277300 PE=3 SV=1	3.35
Q2QLS3	Alpha-amylase/trypsin inhibitor, putative, expressed OS=Oryza sativa subsp. japonica GN=Os12g0630500 PE=2 SV=1	3.07
Q8S702	Glutathione S-transferase GSTU6, putative, expressed OS=Oryza sativa subsp. japonica GN=LOC_Os10g38470 PE=2 SV=1	3.06
Q9XFE4	Peptidylprolyl isomerase OS=Oryza sativa subsp. japonica GN=Os04g0352400 PE=2 SV=2	3.04
Q9FWU4	Os10g0491000 protein OS=Oryza sativa subsp. japonica GN=LOC_Os10g34930 PE=2 SV=1	3.02
Q2R1V2	Barwin, putative, expressed OS=Oryza sativa subsp. japonica GN=LOC_Os11g37950 PE=2 SV=1	2.92
Q851F9	Probable zinc metalloprotease EGY3, chloroplastic OS=Oryza sativa subsp. japonica GN=EGY3 PE=2 SV=1	2.85
A0A0P0XC\	Os08g0205800 protein (Fragment) OS=Oryza sativa subsp. japonica GN=Os08g0205800 PE=4 SV=1	2.83
Q10MP7	Os03g0300400 protein OS=Oryza sativa subsp. japonica GN=Os03g0300400 PE=2 SV=1	2.76

A0A0P0W6	Os04g0107900 protein (Fragment) OS=Oryza sativa subsp. japonica GN=Os04g0107900 PE=4 SV=1	2.74
Q655T1	Phosphoglycerate kinase OS=Oryza sativa subsp. japonica GN=Os06g0668200 PE=2 SV=1	2.72
Q943K7	70 kDa heat shock protein OS=Oryza sativa subsp. japonica GN=Os01g0840100 PE=2 SV=1	2.65
Q0DHF7	Os05g0468800 protein OS=Oryza sativa subsp. japonica GN=Os05g0468800 PE=4 SV=1	2.53
Q0IT26	Os11g0425600 protein OS=Oryza sativa subsp. japonica GN=Os11g0425600 PE=4 SV=1	2.51
Q6ESR4	Dehydrin DHN1 OS=Oryza sativa subsp. japonica GN=DHN1 PE=2 SV=1	2.48
Q0DPU1	Os03g0663500 protein (Fragment) OS=Oryza sativa subsp. japonica GN=Os03g0663500 PE=4 SV=1	2.47
A0A0P0XVI	Alpha-galactosidase OS=Oryza sativa subsp. japonica GN=Os10g0492900 PE=3 SV=1	2.46
A0A0P0WA	Os04g0423600 protein OS=Oryza sativa subsp. japonica GN=Os04g0423600 PE=4 SV=1	2.46
Q94CS9	Probable aquaporin TIP1-2 OS=Oryza sativa subsp. japonica GN=TIP1-2 PE=2 SV=1	2.45
A0A0P0V4	Os01g0571166 protein OS=Oryza sativa subsp. japonica GN=Os01g0571166 PE=4 SV=1	2.43
Q10PW8	DnaK protein, expressed OS=Oryza sativa subsp. japonica GN=Os03g0218500 PE=2 SV=1	2.43
Q306J3	Dirigent protein OS=Oryza sativa subsp. japonica GN=JAC1 PE=2 SV=1	2.42
Q6Z7V2	24.1 kDa heat shock protein, mitochondrial OS=Oryza sativa subsp. japonica GN=HSP24.1 PE=2 SV=1	2.40
Q0IZF1	Os09g0572700 protein OS=Oryza sativa subsp. japonica GN=Os09g0572700 PE=2 SV=1	2.39
A0A0P0XGI	Os08g0425800 protein OS=Oryza sativa subsp. japonica GN=Os08g0425800 PE=4 SV=1	2.35
Q93WY5	Os09g0467200 protein OS=Oryza sativa subsp. japonica GN=Os09g0467200 PE=2 SV=1	2.35
Q7F2P0	Os01g0382000 protein OS=Oryza sativa subsp. japonica GN=Os01g0382000 PE=3 SV=1	2.35
Q0IQK9	Non-specific lipid-transfer protein 1 OS=Oryza sativa subsp. japonica GN=LTP PE=1 SV=1	2.35
B9FCZ7	Os04g0663700 protein OS=Oryza sativa subsp. japonica GN=Os04g0663700 PE=4 SV=1	2.33
Q6L509	Os05g0460000 protein OS=Oryza sativa subsp. japonica GN=Os05g0460000 PE=2 SV=1	2.28
Q7XUW5	23.2 kDa heat shock protein OS=Oryza sativa subsp. japonica GN=HSP23.2 PE=2 SV=2	2.27
Q2QNN5	Lipoxygenase OS=Oryza sativa subsp. japonica GN=Os12g0559200 PE=3 SV=2	2.26
A0A0P0W1	Os03g0661600 protein (Fragment) OS=Oryza sativa subsp. japonica GN=Os03g0661600 PE=4 SV=1	2.26
Q851K1	Germin-like protein 3-6 OS=Oryza sativa subsp. japonica GN=Os03g0694000 PE=2 SV=1	2.25

Q5VP66	Os01g0644000 protein OS=Oryza sativa subsp. japonica GN=Os01g0644000 PE=2 SV=1	2.23
Q337E2	Expressed protein OS=Oryza sativa subsp. japonica GN=Os10g0505900 PE=4 SV=1	2.21
Q0JIK5	Os01g0795200 protein OS=Oryza sativa subsp. japonica GN=Os01g0795200 PE=3 SV=1	2.21
Q7F1U0	Peroxidase OS=Oryza sativa subsp. japonica GN=OJ1167_G06.125 PE=2 SV=1	2.20
Q6Z563	Os08g0412800 protein OS=Oryza sativa subsp. japonica GN=Os08g0412800 PE=2 SV=1	2.19
Q7G649	Expressed protein OS=Oryza sativa subsp. japonica GN=LOC_Os10g18340 PE=2 SV=1	2.19
Q653Y0	Os06g0681200 protein OS=Oryza sativa subsp. japonica GN=Os06g0681200 PE=2 SV=1	2.18
Q6F2Y7	Chaperone protein ClpB1 OS=Oryza sativa subsp. japonica GN=CLPB1 PE=2 SV=1	2.16
Q42993	Chitinase 1 OS=Oryza sativa subsp. japonica GN=Cht1 PE=2 SV=1	2.15
Q6AUF2	Os05g0565400 protein OS=Oryza sativa subsp. japonica GN=Os05g0565400 PE=2 SV=1	2.14
A0A0N7KP:	Os07g0683600 protein OS=Oryza sativa subsp. japonica GN=Os07g0683600 PE=4 SV=1	2.13
Q6ZCR3	Germin-like protein 8-12 OS=Oryza sativa subsp. japonica GN=Os08g0231400 PE=2 SV=1	2.12
Q338P6	Os10g0389200 protein OS=Oryza sativa subsp. japonica GN=Os10g0389200 PE=2 SV=1	2.12
Q688T8	Glucose-1-phosphate adenylyltransferase large subunit 3, chloroplastic/amyloplastic OS=Oryza sativa subsp. japonica GN=AGPL3 PE=1 SV=1	2.12
Q6Z4I3	Thioredoxin H2-1 OS=Oryza sativa subsp. japonica GN=Os07g0190800 PE=2 SV=1	2.09
Q53K52	Protein PEP-RELATED DEVELOPMENT ARRESTED 1 homolog, chloroplastic OS=Oryza sativa subsp. japonica GN=Os11g0425300 PE=2 SV=1	2.09
Q60ER3	Os05g0393400 protein OS=Oryza sativa subsp. japonica GN=Os05g0393400 PE=2 SV=1	2.08
Q7F164	Os01g0940700 protein OS=Oryza sativa subsp. japonica GN=P0432C03.10-1 PE=2 SV=1	2.06
B7E9D7	Os01g0124650 protein OS=Oryza sativa subsp. japonica GN=Os01g0124650 PE=2 SV=1	2.03
Q2QLS9	Os12g0629700 protein OS=Oryza sativa subsp. japonica GN=LOC_Os12g43430 PE=2 SV=1	2.02
Q7XPV4	OSJNBa0088H09.2 protein OS=Oryza sativa subsp. japonica GN=Os04g0683700 PE=4 SV=1	2.02
Q84YK8	Probable lipoxygenase 8, chloroplastic OS=Oryza sativa subsp. japonica GN=CM-LOX2 PE=2 SV=1	1.99
A0A0P0W\	Os06g0323100 protein (Fragment) OS=Oryza sativa subsp. japonica GN=Os06g0323100 PE=4 SV=1	1.98
Q8H367	Os07g0413800 protein OS=Oryza sativa subsp. japonica GN=Os07g0413800 PE=2 SV=1	1.98

Q0J0C4	Os09g0517000 protein OS=Oryza sativa subsp. japonica GN=Os09g0517000 PE=2 SV=1	1.98
Q2R5M2	Carboxypeptidase OS=Oryza sativa subsp. japonica GN=LOC_Os11g24510 PE=3 SV=1	1.98
Q10N98	33 kDa secretory protein, putative, expressed OS=Oryza sativa subsp. japonica GN=Os03g0277600 PE=2 SV=1	1.97
Q0DI48	Thioredoxin-like fold domain-containing protein MRL7L homolog, chloroplastic OS=Oryza sativa subsp. japonica GN=MRL7L PE=2 SV=1	1.96
Q8L4V6	Glutathione S-transferase GSTU6, putative, expressed OS=Oryza sativa subsp. japonica GN=LOC_Os10g38780 PE=2 SV=1	1.95
Q8S1C7	Cytochrome P450 (CYP72C)-like OS=Oryza sativa subsp. japonica GN=CYP72A32 PE=2 SV=1	1.94
Q5QMT0	Beta-glucosidase 1 OS=Oryza sativa subsp. japonica GN=BGLU1 PE=2 SV=1	1.92
Q6YXZ3	Os02g0139100 protein OS=Oryza sativa subsp. japonica GN=Os02g0139100 PE=2 SV=1	1.92
Q5ZBR8	Os01g0795000 protein OS=Oryza sativa subsp. japonica GN=Os01g0795000 PE=4 SV=1	1.92
Q7XIX0	Peroxidase OS=Oryza sativa subsp. japonica GN=OJ1167_G06.129 PE=3 SV=1	1.91
A0A0P0W3	Os03g0750100 protein OS=Oryza sativa subsp. japonica GN=Os03g0750100 PE=4 SV=1	1.91
Q5ZAV6	Os01g0783600 protein OS=Oryza sativa subsp. japonica GN=Os01g0783600 PE=2 SV=1	1.90
P31110	Thaumatin-like protein OS=Oryza sativa subsp. japonica GN=Os12g0628600 PE=1 SV=1	1.90
A5HEI2	Bowman-Birk type proteinase inhibitor A OS=Oryza sativa subsp. japonica GN=pinA PE=2 SV=1	1.90
Q5VRH4	Homogentisate 1,2-dioxygenase OS=Oryza sativa subsp. japonica GN=HGO PE=2 SV=1	1.88
Q94E74	Os01g0511100 protein OS=Oryza sativa subsp. japonica GN=Os01g0511100 PE=2 SV=1	1.88
Q67WJ2	ATP-dependent zinc metalloprotease FTSH 6, chloroplastic OS=Oryza sativa subsp. japonica GN=FTSH6 PE=3 SV=1	1.88
Q8H3A4	ABC transporter permease protein-like protein OS=Oryza sativa subsp. japonica GN=P0616D06.112-1 PE=2 SV=1	1.87
Q6K623	Os02g0612900 protein OS=Oryza sativa subsp. japonica GN=Os02g0612900 PE=2 SV=1	1.87
Q6H5Y1	Os02g0228300 protein OS=Oryza sativa subsp. japonica GN=Os02g0228300 PE=4 SV=1	1.87
Q2QQS1	KE2 family protein, expressed OS=Oryza sativa subsp. japonica GN=Os12g0485800 PE=2 SV=1	1.87
Q94LP4	(RAP Annotation release2) 2OG-Fe(II) oxygenase domain containing protein OS=Oryza sativa subsp. japonica GN=OSJNBa0042H09.28 PE=2 SV=1	1.86
Q8LNZ3	UDP-glucose 4-epimerase 1 OS=Oryza sativa subsp. japonica GN=UGE-1 PE=2 SV=1	1.86
Q10SR3	70 kDa heat shock protein OS=Oryza sativa subsp. japonica GN=Os03g0113700 PE=2 SV=1	1.84

Q9SXF8	Aquaporin PIP 1-3 OS=Oryza sativa subsp. japonica GN=PIP1-3 PE=2 SV=2	1.84
Q75T45	Os12g0555000 protein OS=Oryza sativa subsp. japonica GN=RSOsPR10 PE=2 SV=1	1.84
Q5ZCB1	Os01g0124000 protein OS=Oryza sativa subsp. japonica GN=Os01g0124000 PE=2 SV=1	1.84
Q6ZFM7	Os07g0582400 protein OS=Oryza sativa subsp. japonica GN=Os07g0582400 PE=3 SV=1	1.82
Q75M70	Expressed protein OS=Oryza sativa subsp. japonica GN=Os03g0381500 PE=2 SV=1	1.82
Q7XCS3	Cys/Met metabolism PLP-dependent enzyme family protein, expressed OS=Oryza sativa subsp. japonica GN=Os10g0517500 PE=2 SV=1	1.81
A0A0P0VQ	Os02g0783625 protein OS=Oryza sativa subsp. japonica GN=Os02g0783625 PE=4 SV=1	1.81
Q69JX7	Drought-induced S-like ribonuclease OS=Oryza sativa subsp. japonica GN=Os09g0537700 PE=2 SV=1	1.81
Q6ZCF3	Os08g0205400 protein OS=Oryza sativa subsp. japonica GN=Os08g0205400 PE=2 SV=1	1.81
Q10LP5	Sucrose synthase 4 OS=Oryza sativa subsp. japonica GN=SUS4 PE=2 SV=1	1.80
Q5JJV6	Os01g0965900 protein OS=Oryza sativa subsp. japonica GN=Os01g0965900 PE=2 SV=1	1.80
Q5SMV5	Carboxypeptidase OS=Oryza sativa subsp. japonica GN=Os06g0186400 PE=2 SV=1	1.79
Q6K4D9	Os09g0363700 protein OS=Oryza sativa subsp. japonica GN=Os09g0363700 PE=2 SV=1	1.79
A0A0P0VVI	Os03g0248200 protein (Fragment) OS=Oryza sativa subsp. japonica GN=Os03g0248200 PE=3 SV=1	1.78
Q5VRM0	Acyl-CoA-binding domain-containing protein 2 OS=Oryza sativa subsp. japonica GN=ACBP2 PE=1 SV=1	1.78
Q0D840	Thioredoxin H1 OS=Oryza sativa subsp. japonica GN=TRXH PE=1 SV=1	1.77
Q0DAI4	Os06g0651000 protein OS=Oryza sativa subsp. japonica GN=Os06g0651000 PE=4 SV=1	1.75
Q6ZFJ9	60 kDa chaperonin beta subunit OS=Oryza sativa subsp. japonica GN=Os02g0102900 PE=2 SV=1	1.75
Q75LD9	Os03g0843300 protein OS=Oryza sativa subsp. japonica GN=OSJNBa0032G11.5 PE=2 SV=1	1.74
Q10Q92	CUE domain containing protein, expressed OS=Oryza sativa subsp. japonica GN=Os03g0205000 PE=2 SV=1	1.73
Q6ITC5	Cell death-related protein OS=Oryza sativa subsp. japonica GN=A2 PE=2 SV=1	1.73
Q65XA0	Probable glutathione S-transferase DHAR1, cytosolic OS=Oryza sativa subsp. japonica GN=DHAR1 PE=1 SV=1	1.73
Q6F391	Expressed protein OS=Oryza sativa subsp. japonica GN=OSJNBb0021G19.8 PE=2 SV=1	1.72
Q6Z493	Protein DETOXIFICATION OS=Oryza sativa subsp. japonica GN=Os07g0502200 PE=3 SV=1	1.71

Q75M67	Expressed protein OS=Oryza sativa subsp. japonica GN=Os03g0381300 PE=4 SV=1	1.71
Q7Y092	Antitermination NusB domain-containing protein, putative, expressed OS=Oryza sativa subsp. japonica GN=OSJNBa0075A22.21 PE=4 SV=1	1.71
P38419	Lipoxygenase 7, chloroplastic OS=Oryza sativa subsp. japonica GN=CM-LOX1 PE=2 SV=2	1.68
Q5QLQ5	Os01g0667200 protein OS=Oryza sativa subsp. japonica GN=Os01g0667200 PE=2 SV=1	1.68
Q6Z7K5	Metal tolerance protein 3 OS=Oryza sativa subsp. japonica GN=MTP3 PE=2 SV=1	1.68
Q2QNV2	Fiber protein Fb19, putative, expressed OS=Oryza sativa subsp. japonica GN=LOC_Os12g36640 PE=2 SV=1	1.67
Q2QXL3	ACT domain-containing protein, putative, expressed OS=Oryza sativa subsp. japonica GN=Os12g0152700 PE=2 SV=1	1.67
C7J056	Os03g0859600 protein (Fragment) OS=Oryza sativa subsp. japonica GN=Os03g0859600 PE=3 SV=1	1.67
A0A0P0WN	Os05g0432700 protein (Fragment) OS=Oryza sativa subsp. japonica GN=Os05g0432700 PE=4 SV=1	1.67
Q5KQJ3	Os05g0181901 protein OS=Oryza sativa subsp. japonica GN=Os05g0182000 PE=4 SV=1	1.67
Q10PD0	Purple acid phosphatase OS=Oryza sativa subsp. japonica GN=LOC_Os03g13540 PE=2 SV=1	1.66
Q5NA77	C2 domain-containing protein-like OS=Oryza sativa subsp. japonica GN=Os01g0242600 PE=2 SV=1	1.66
Q941F5	Os11g0592200 protein OS=Oryza sativa subsp. japonica GN=PR4 PE=2 SV=1	1.66
Q8W2X5	Flavanone 3-dioxygenase 2 OS=Oryza sativa subsp. japonica GN=F3H-2 PE=1 SV=1	1.64
Q10QP0	Os03g0189400 protein OS=Oryza sativa subsp. japonica GN=LOC_Os03g08999 PE=2 SV=1	1.64
Q6YVU4	Os07g0539300 protein OS=Oryza sativa subsp. japonica GN=P0696F12.36-1 PE=2 SV=1	1.63
C7J745	Os09g0491852 protein OS=Oryza sativa subsp. japonica GN=Os09g0491852 PE=4 SV=1	1.63
Q8H6G8	Probable inorganic phosphate transporter 1-8 OS=Oryza sativa subsp. japonica GN=PHT1-8 PE=2 SV=1	1.62
Q6Z312	bZIP transcription factor 23 OS=Oryza sativa subsp. japonica GN=BZIP23 PE=2 SV=1	1.62
A0A0P0VC	Os01g0949750 protein (Fragment) OS=Oryza sativa subsp. japonica GN=Os01g0949750 PE=4 SV=1	1.62
O49827	Chitinase OS=Oryza sativa subsp. japonica GN=Os01g0860500 PE=2 SV=1	1.61
Q7XI43	Metal-transporting P-type ATPase-like protein OS=Oryza sativa subsp. japonica GN=P0524E08.111 PE=2 SV=1	1.61
Q0JIL1	Probable nucleoredoxin 2 OS=Oryza sativa subsp. japonica GN=Os01g0794400 PE=2 SV=1	1.61
Q94DD0	Os01g0859200 protein OS=Oryza sativa subsp. japonica GN=Os01g0859200 PE=2 SV=1	1.61

Q9FRA7	REF/SRPP-like protein Os05g0151300/LOC_Os05g05940 OS=Oryza sativa subsp. japonica GN=Os05g0151300 PE=2 SV=2	1.60
Q6AVZ3	Peroxidase OS=Oryza sativa subsp. japonica GN=Os05g0135500 PE=2 SV=1	1.60
Q7XJ02	Probable L-ascorbate peroxidase 7, chloroplastic OS=Oryza sativa subsp. japonica GN=APX7 PE=2 SV=1	1.60
Q6YZI5	Os08g0558900 protein OS=Oryza sativa subsp. japonica GN=Os08g0558900 PE=2 SV=1	1.59
Q10MJ3	Os03g0306900 protein OS=Oryza sativa subsp. japonica GN=Os03g0306900 PE=2 SV=1	1.59
O04226	Delta-1-pyrroline-5-carboxylate synthase 1 OS=Oryza sativa subsp. japonica GN=P5CS1 PE=2 SV=2	1.58
Q6K3R5	Sodium/calcium exchanger NCL2 OS=Oryza sativa subsp. japonica GN=NCL2 PE=2 SV=2	1.58
Q6AUV3	Os03g0430000 protein OS=Oryza sativa subsp. japonica GN=OSJNBa0091B22.9 PE=2 SV=1	1.58
Q9FP25	Os01g0303000 protein OS=Oryza sativa subsp. japonica GN=P0035H10.18 PE=2 SV=1	1.57
Q6J657	Cell death associated protein OS=Oryza sativa subsp. japonica GN=Os05g0410200 PE=2 SV=1	1.57
Q5ZAV7	Os01g0783500 protein OS=Oryza sativa subsp. japonica GN=Os01g0783500 PE=2 SV=1	1.57
Q6ZBK6	Os08g0519400 protein OS=Oryza sativa subsp. japonica GN=Os08g0519400 PE=4 SV=1	1.56
Q10L32	Peptide methionine sulfoxide reductase B5 OS=Oryza sativa subsp. japonica GN=MSRB5 PE=2 SV=1	1.56
Q0DCI1	Pyrophosphate--fructose 6-phosphate 1-phosphotransferase subunit alpha OS=Oryza sativa subsp. japonica GN=Os06g0326400 PE=2 SV=1	1.55
Q0D5S1	Os07g0539900 protein OS=Oryza sativa subsp. japonica GN=Os07g0539900 PE=2 SV=1	1.55
Q6Z244	Os08g0480200 protein OS=Oryza sativa subsp. japonica GN=Os08g0480200 PE=2 SV=1	1.54
Q69P84	Aldehyde dehydrogenase OS=Oryza sativa subsp. japonica GN=OJ1344_B01.27-1 PE=2 SV=1	1.54
Q2QVJ8	NADP-dependent oxidoreductase P2, putative, expressed OS=Oryza sativa subsp. japonica GN=Os12g0226700 PE=4 SV=1	1.54
B9FCW0	Os04g0652700 protein OS=Oryza sativa subsp. japonica GN=Os04g0652700 PE=4 SV=1	1.53
Q5QL78	Os01g0524700 protein OS=Oryza sativa subsp. japonica GN=Os01g0524700 PE=2 SV=1	1.53
Q5Z7I5	Os06g0548200 protein OS=Oryza sativa subsp. japonica GN=Os06g0548200 PE=3 SV=1	1.53
Q8S1G9	Os01g0796400 protein OS=Oryza sativa subsp. japonica GN=P0699H05.27-1 PE=2 SV=1	1.53
Q0J8A4	Glyceraldehyde-3-phosphate dehydrogenase 1, cytosolic OS=Oryza sativa subsp. japonica GN=GAPC1 PE=1 SV=1	1.53
Q6H7E4	Thioredoxin M1, chloroplastic OS=Oryza sativa subsp. japonica GN=Os02g0639900 PE=2 SV=1	1.53

Q651X9	Os09g0477900 protein OS=Oryza sativa subsp. japonica GN=Os09g0477900 PE=3 SV=1	1.53
Q75GT3	Chaperone protein ClpB2, chloroplastic OS=Oryza sativa subsp. japonica GN=CLPB2 PE=2 SV=1	1.53
P48642	Glutathione reductase, cytosolic OS=Oryza sativa subsp. japonica GN=GRC2 PE=2 SV=2	1.53
Q5Z5T3	Os06g0567900 protein OS=Oryza sativa subsp. japonica GN=Os06g0567900 PE=3 SV=1	1.52
Q9FW24	Os05g0102900 protein OS=Oryza sativa subsp. japonica GN=Os05g0102900 PE=2 SV=2	1.52
Q6AVR6	Os03g0840200 protein OS=Oryza sativa subsp. japonica GN=Os03g0840200 PE=2 SV=1	1.52
Q7X7N2	Arginase 1, mitochondrial OS=Oryza sativa subsp. japonica GN=ARG1 PE=2 SV=1	1.52
Q948T6	Lactoylglutathione lyase OS=Oryza sativa subsp. japonica GN=GLYI-11 PE=1 SV=2	1.52
Q9LGB2	Os01g0132000 protein OS=Oryza sativa subsp. japonica GN=Os01g0132000 PE=2 SV=1	1.51
A0A0P0WL	Os05g0364600 protein (Fragment) OS=Oryza sativa subsp. japonica GN=Os05g0364600 PE=4 SV=1	1.51
Q5VRJ8	Peroxisomal membrane protein 11-5 OS=Oryza sativa subsp. japonica GN=PEX11-5 PE=2 SV=1	1.51
Q7XSN6	Germin-like protein 4-1 OS=Oryza sativa subsp. japonica GN=Os04g0617900 PE=2 SV=2	1.51
Q75LJ3	Electron transfer flavoprotein subunit alpha, mitochondrial OS=Oryza sativa subsp. japonica GN=ETFA PE=2 SV=1	1.51
Q5Z9H8	Peptidyl-prolyl cis-trans isomerase OS=Oryza sativa subsp. japonica GN=P0655A07.5-2 PE=2 SV=1	1.51
Q688X8	'putative heat shock protein, hsp40 OS=Oryza sativa subsp. japonica GN=Os05g0562300 PE=2 SV=1	1.51
Q6ZLJ9	Os07g0209100 protein OS=Oryza sativa subsp. japonica GN=Os07g0209100 PE=4 SV=1	1.51
Q7Y0E8	Probable nucleoredoxin 1-1 OS=Oryza sativa subsp. japonica GN=Os03g0405500 PE=2 SV=1	1.50
A0A0P0UZ:	Os01g0190000 protein OS=Oryza sativa subsp. japonica GN=Os01g0190000 PE=4 SV=1	1.50
Q6ETD9	Calmodulin-binding protein-like OS=Oryza sativa subsp. japonica GN=Os02g0105500 PE=2 SV=1	1.50
Q6K6Q1	Phenylalanine ammonia-lyase OS=Oryza sativa subsp. japonica GN=Os02g0626400 PE=2 SV=1	1.50
B9FRA2	Os06g0127500 protein OS=Oryza sativa subsp. japonica GN=Os06g0127500 PE=4 SV=1	1.50
A0A0P0W6	Os03g0859600 protein (Fragment) OS=Oryza sativa subsp. japonica GN=Os03g0859600 PE=3 SV=1	1.50
Q7XQ93	OSJNBa0018M05.15 protein OS=Oryza sativa subsp. japonica GN=Os04g0674700 PE=2 SV=2	1.50
Q652L6	Monodehydroascorbate reductase 3, cytosolic OS=Oryza sativa subsp. japonica GN=MDAR3 PE=1 SV=1	1.50
Q10LW8	Hydroxyacylglutathione hydrolase, putative, expressed OS=Oryza sativa subsp. japonica GN=Os03g0332400 PE=2 SV=1	1.50

NIP_ S4 vs C0_ Proteins decreased in abundance

Accession	Description	FC	
O64437	Inositol-3-phosphate synthase 1 OS=Oryza sativa subsp. japonica GN=RINO1 PE=1 SV=2	0.36	-2.77
Q6K826	Os02g0781400 protein OS=Oryza sativa subsp. japonica GN=OJ1369_G08.10-1 PE=2 SV=1	0.41	-2.42
Q8RZQ8	Bidirectional sugar transporter SWEET1a OS=Oryza sativa subsp. japonica GN=SWEET1A PE=2 SV=1	0.42	-2.39
Q7XKF3	Protochlorophyllide reductase A, chloroplastic OS=Oryza sativa subsp. japonica GN=PORA PE=2 SV=1	0.43	-2.34
Q5NAM3	Branched-chain amino acid aminotransferase-like OS=Oryza sativa subsp. japonica GN=Os01g0238500 PE=4 SV=1	0.49	-2.05
Q7XI92	Os07g0580900 protein OS=Oryza sativa subsp. japonica GN=Os07g0580900 PE=1 SV=1	0.49	-2.04
Q69TG9	Glycine-rich protein-like OS=Oryza sativa subsp. japonica GN=Os06g0216700 PE=2 SV=1	0.50	-2.01
Q6ATB2	Probable GTP diphosphokinase CRSH2, chloroplastic OS=Oryza sativa subsp. japonica GN=CRSH2 PE=2 SV=1	0.51	-1.96
Q0D5I5	Os07g0558300 protein OS=Oryza sativa subsp. japonica GN=Os07g0558300 PE=2 SV=1	0.51	-1.94
Q6ZLK8	Os07g0134000 protein OS=Oryza sativa subsp. japonica GN=OJ1118_D07.26-1 PE=2 SV=1	0.52	-1.94
Q6ET88	Os02g0668100 protein OS=Oryza sativa subsp. japonica GN=Os02g0668100 PE=1 SV=1	0.52	-1.94
Q2QND9	Expressed protein OS=Oryza sativa subsp. japonica GN=Os12g0569200 PE=4 SV=1	0.52	-1.92
A0A0P0X7!	Os07g0538700 protein OS=Oryza sativa subsp. japonica GN=Os07g0538700 PE=4 SV=1	0.52	-1.91
Q6H6D2	Porphobilinogen deaminase, chloroplastic OS=Oryza sativa subsp. japonica GN=HEMC PE=2 SV=1	0.52	-1.91
Q6YY42	Os02g0589000 protein OS=Oryza sativa subsp. japonica GN=Os02g0589000 PE=4 SV=1	0.55	-1.82
Q0IPL3	Os12g0189300 protein OS=Oryza sativa subsp. japonica GN=Os12g0189300 PE=4 SV=1	0.57	-1.77
Q10LR9	Uroporphyrinogen decarboxylase 2, chloroplastic OS=Oryza sativa subsp. japonica GN=Os03g0337600 PE=3 SV=1	0.57	-1.76
Q10M50	Magnesium-chelatase subunit ChlH, chloroplastic OS=Oryza sativa subsp. japonica GN=CHLH PE=1 SV=1	0.58	-1.71
Q6KA61	Os02g0285800 protein OS=Oryza sativa subsp. japonica GN=Os02g0285800 PE=4 SV=1	0.59	-1.70
Q33AG7	CMV 1a interacting protein 1, putative, expressed OS=Oryza sativa subsp. japonica GN=Os10g0181600 PE=2 SV=2	0.59	-1.69
Q10J01	Expressed protein OS=Oryza sativa subsp. japonica GN=Os03g0439700 PE=4 SV=1	0.59	-1.69
Q10SD2	Expressed protein OS=Oryza sativa subsp. japonica GN=Os03g0126300 PE=2 SV=1	0.60	-1.68
Q0D7H8	Os07g0245100 protein (Fragment) OS=Oryza sativa subsp. japonica GN=Os07g0245100 PE=4 SV=1	0.60	-1.66

Q8W2X4	Expressed protein OS=Oryza sativa subsp. japonica GN=OSJNBb0060I05.14 PE=2 SV=1	0.60	-1.66
Q84QW4	Os08g0524400 protein OS=Oryza sativa subsp. japonica GN=OJ1191_A10.104 PE=4 SV=1	0.60	-1.66
Q10LH0	Divinyl chlorophyllide a 8-vinyl-reductase, chloroplastic OS=Oryza sativa subsp. japonica GN=DVR PE=3 SV=1	0.61	-1.64
Q6ZA95	Os08g0433300 protein OS=Oryza sativa subsp. japonica GN=Os08g0433300 PE=4 SV=1	0.62	-1.62
B7FA34	Os05g0548900 protein OS=Oryza sativa subsp. japonica GN=Os05g0548900 PE=2 SV=1	0.62	-1.61
Q5Z8V9	Delta-aminolevulinic acid dehydratase, chloroplastic OS=Oryza sativa subsp. japonica GN=HEMB PE=2 SV=1	0.63	-1.60
O22567	1-deoxy-D-xylulose-5-phosphate synthase 1, chloroplastic OS=Oryza sativa subsp. japonica GN=CLA1 PE=2 SV=2	0.63	-1.59
Q53RM0	Magnesium-chelatase subunit ChlI, chloroplastic OS=Oryza sativa subsp. japonica GN=CHLI PE=1 SV=1	0.63	-1.59
Q9AXB0	Uroporphyrinogen decarboxylase 1, chloroplastic OS=Oryza sativa subsp. japonica GN=Os01g0622300 PE=2 SV=1	0.63	-1.59
Q7F9I1	Chaperone protein ClpC1, chloroplastic OS=Oryza sativa subsp. japonica GN=CLPC1 PE=2 SV=2	0.63	-1.58
Q0J0M2	Acyl-[acyl-carrier-protein] hydrolase (Fragment) OS=Oryza sativa subsp. japonica GN=Os09g0505300 PE=3 SV=1	0.63	-1.58
Q84S01	Os08g0152700 protein OS=Oryza sativa subsp. japonica GN=OJ1349_D05.118 PE=2 SV=1	0.64	-1.57
A0A0P0WC	Os03g0593200 protein (Fragment) OS=Oryza sativa subsp. japonica GN=Os03g0593200 PE=4 SV=1	0.64	-1.57
Q7Y168	Expressed protein OS=Oryza sativa subsp. japonica GN=Os03g0375200 PE=4 SV=1	0.64	-1.57
Q7XN02	OSJNBb0038F03.9 protein OS=Oryza sativa subsp. japonica GN=OSJNBb0038F03.9 PE=4 SV=1	0.64	-1.56
Q0DAQ8	Os06g0638200 protein (Fragment) OS=Oryza sativa subsp. japonica GN=Os06g0638200 PE=4 SV=1	0.64	-1.55
Q6Z2T6	Geranylgeranyl diphosphate reductase, chloroplastic OS=Oryza sativa subsp. japonica GN=CHLP PE=2 SV=1	0.65	-1.55
Q5VPQ6	Os06g0119600 protein OS=Oryza sativa subsp. japonica GN=Os06g0119600 PE=2 SV=1	0.65	-1.55
B7EIQ8	Os02g0125700 protein OS=Oryza sativa subsp. japonica GN=Os02g0125700 PE=2 SV=1	0.65	-1.54
Q7XL03	Chaperone protein ClpD2, chloroplastic OS=Oryza sativa subsp. japonica GN=CLPD2 PE=2 SV=2	0.65	-1.53
Q7F9Y6	OSJNBa0086O06.22 protein OS=Oryza sativa subsp. japonica GN=Os04g0591000 PE=2 SV=1	0.66	-1.52
Q69S79	Os02g0575500 protein OS=Oryza sativa subsp. japonica GN=P0703B01.21-1 PE=4 SV=1	0.66	-1.52
Q5SNH7	Os01g0191100 protein OS=Oryza sativa subsp. japonica GN=Os01g0191100 PE=2 SV=1	0.66	-1.52
Q5TKG2	Os05g0594500 protein OS=Oryza sativa subsp. japonica GN=Os05g0594500 PE=4 SV=1	0.67	-1.50
